# Photoswitchable Probes of Oxytocin and Vasopressin

**DOI:** 10.1021/acs.jmedchem.3c01415

**Published:** 2023-10-19

**Authors:** Ulrike Wirth, Konstantin Raabe, Predrag Kalaba, Erik Keimpema, Markus Muttenthaler, Burkhard König

**Affiliations:** † Institute of Organic Chemistry, Department of Chemistry and Pharmacy, University of Regensburg, Universitätsstraße 31, 93053 Regensburg, Germany; ‡ Institute of Biological Chemistry, Department of Chemistry, University of Vienna, Währinger Straße 38, 1090 Vienna, Austria; § Medical University of Vienna, Center for Brain Research, Department of Molecular Neurosciences, Spitalgasse 4, 1090 Vienna, Austria; # Institute for Molecular Bioscience, The University of Queensland, St. Lucia, 4072, Brisbane, Australia

## Abstract

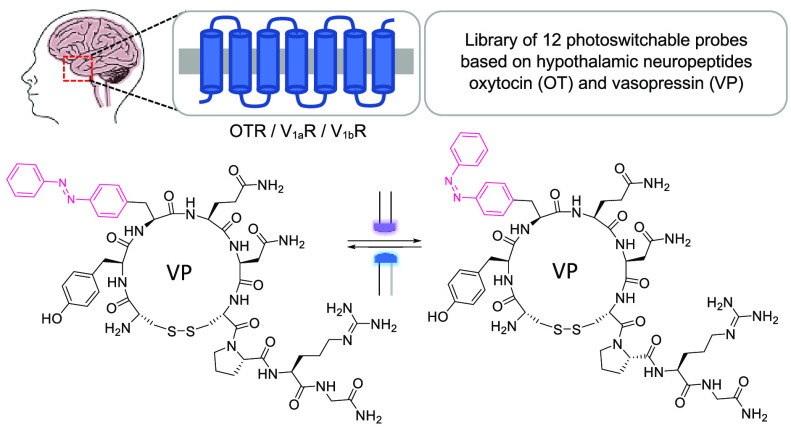

Oxytocin (OT) and vasopressin (VP) are related neuropeptides that regulate many biological processes. In humans, OT and VP act via four G protein-coupled receptors, OTR, V_1a_R, V_1b_R, and V_2_R (VPRs), which are associated with several disorders. To investigate the therapeutic potential of these receptors, particularly in the receptor-dense areas of the brain, molecular probes with a high temporal and spatial resolution are required. Such a spatiotemporal resolution can be achieved by incorporating photochromic moieties into OT and VP. Here, we report the design, synthesis, and (photo)pharmacological characterization of 12 OT- and VP-derived photoprobes using different modification strategies. Despite OT’s and VP’s sensitivity toward structural changes, we identified two photoprobes with good potency and photoswitch window for investigating the OTR and V_1b_R. These photoprobes should be of high value for producing cutting-edge photocontrollable peptide probes for the study of dynamic and kinetic receptor activation processes in specific regions of the brain.

## Introduction

Oxytocin (OT) and vasopressin (VP) are two closely related hypothalamic neuropeptides that play crucial roles in both the central and peripheral nervous systems, regulating a wide range of physiological processes.^1–4^ OT is commonly referred to as the “love hormone” due to its important central roles in social bonding and maternal behavior, but also its peripheral functions during parturition and breastfeeding.^5–9^ The less famous but not less important VP regulates peripherally water homeostasis^1,10–12^ and blood pressure^13–15^ and is centrally involved in social behavior that can be described as opposing or complementary to OT, including aggression and fear.^1,16,17^

**Figure 1:**
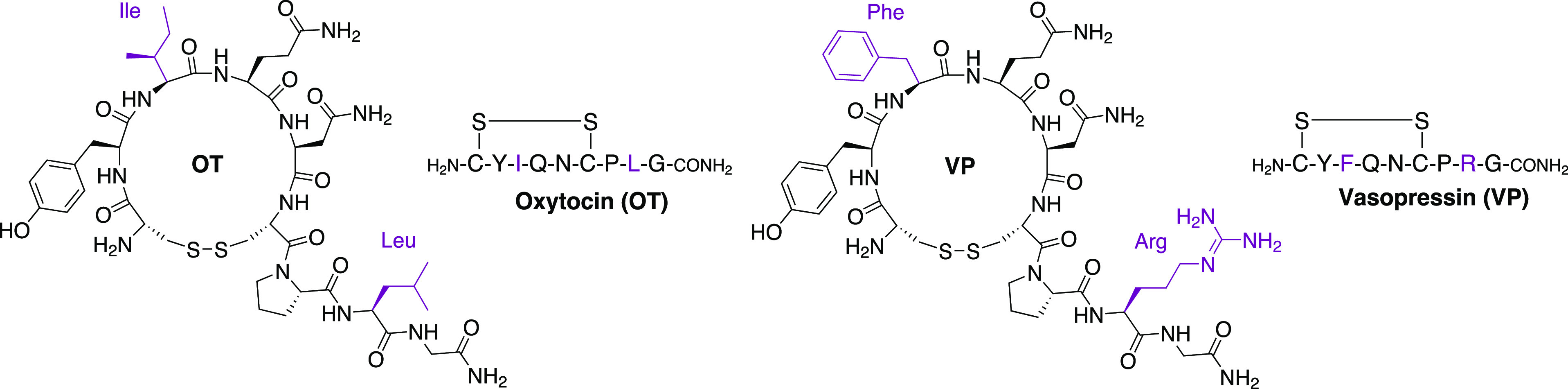
Structures of oxytocin (OT) and vasopressin (VP). The amino acid residues that differ between OT and VP are highlighted in purple.

OT and VP are nonapeptide paralogs that consist of a six-residue macrocycle stabilized by a disulfide bridge between positions 1 and 6 followed by a three-residue exocyclic amidated C-terminal tail. OT and VP differ by only two amino acids (Figure 1), VP containing Phe^3^ and Arg^8^, and OT containing Ile^3^ and Leu^8^.^10,18^ In mammals, these neuropeptides exert their effects through four receptors, namely, OTR, V_1a_R, V_1b_R, and V_2_R, all belonging to the class A of G protein-coupled receptors (GPCRs).^19,20^ Due to the high sequence and structural similarity between the receptors, OT and VP can also activate VPRs and OTR, respectively, and in vivo differences in signaling primarily stem from variances in the receptor expression in different tissues and organs.^21–23^ The lack of specificity of OT and VP limits their usefulness as pharmacological tools and specific drug action.^24^ Nevertheless, OT is clinically used to induce and progress labor and to prevent postpartum bleeding (OTR upregulated),^25,26^ whereas VP is used to medicate postoperative gastrointestinal bleeding or for the treatment of vasodilatory shock (V_1a_R upregulated).^27,28^ Beyond that, OT and VP and their receptors are being investigated in their involvement in autism, schizophrenia, and Prader–Willi syndrome, presenting potential targets and treatment options for improving social conditions and reducing social deficits related to such neurodevelopmental disorders.^2,29–32^

Considerable efforts have been made to develop receptor-selective agonists and antagonists for investigating receptor subtype-specific therapeutic intervention.^23,33^ Although this has propelled the field forward in the past three decades, these probes lack the necessary spatiotemporal resolution to dissect the neuronal circuits influenced by OT and VP. Photopharmacology presents a promising solution to this challenge.^33–35^ By harnessing light as an external and orthogonal stimulus, precise spatiotemporal control over ligand activity can be achieved. This approach has been successful with several small-molecule ligands^36–38^ and, to a lesser extent, with peptide ligands,^39,40^ for example, for the photoregulation of the glucagon-like-peptide-1-receptor,^41^ the natriuretic peptide receptor A,^42^ or for the clathrin-mediated endocytosis.^43^

To obtain light-responsive probes, a photochromic moiety must be incorporated into or attached to a ligand. Some of the most widely used photoswitches are azobenzenes, which enable rapid and reversible switching between their *E*- and *Z*-isomers, resulting in significant changes in a molecular geometry.^34,44,45^ Ideally, the switching mechanism toggles the ligands’ function between agonism and antagonism, or between an inactive and active state (either agonism or antagonism).^34,46^ The principles of photopharmacology have been applied to several GPCRs, including the dopamine receptors,^47,48^ the μ-opioid receptor,^49,50^ the histamine H_3_ receptor,^51^ and the β_2_-receptor.^52^ Notably, a photoswitchable V_2_R antagonist was recently developed based on small-molecule lixivaptan. This innovative approach enables control over the ligand’s residence time at the receptor using light, representing a new kinetic strategy in photopharmacology.^53^

In this study, we present the design, synthesis, photochemical characterization, and biological evaluation of the first photoswitchable derivatives of OT and VP. Various design strategies were employed, resulting in a series of photoprobes based on the structures of OT and VP. The synthesized probes were photochemically characterized by UV/vis spectroscopy and reversed-phase (RP)-HPLC analysis and pharmacologically tested at the OTR and VPRs to evaluate the altered and photoswitchable receptor activation profiles of these new light-controllable probes.

## Results and Discussion

### Design

**Figure 2:**
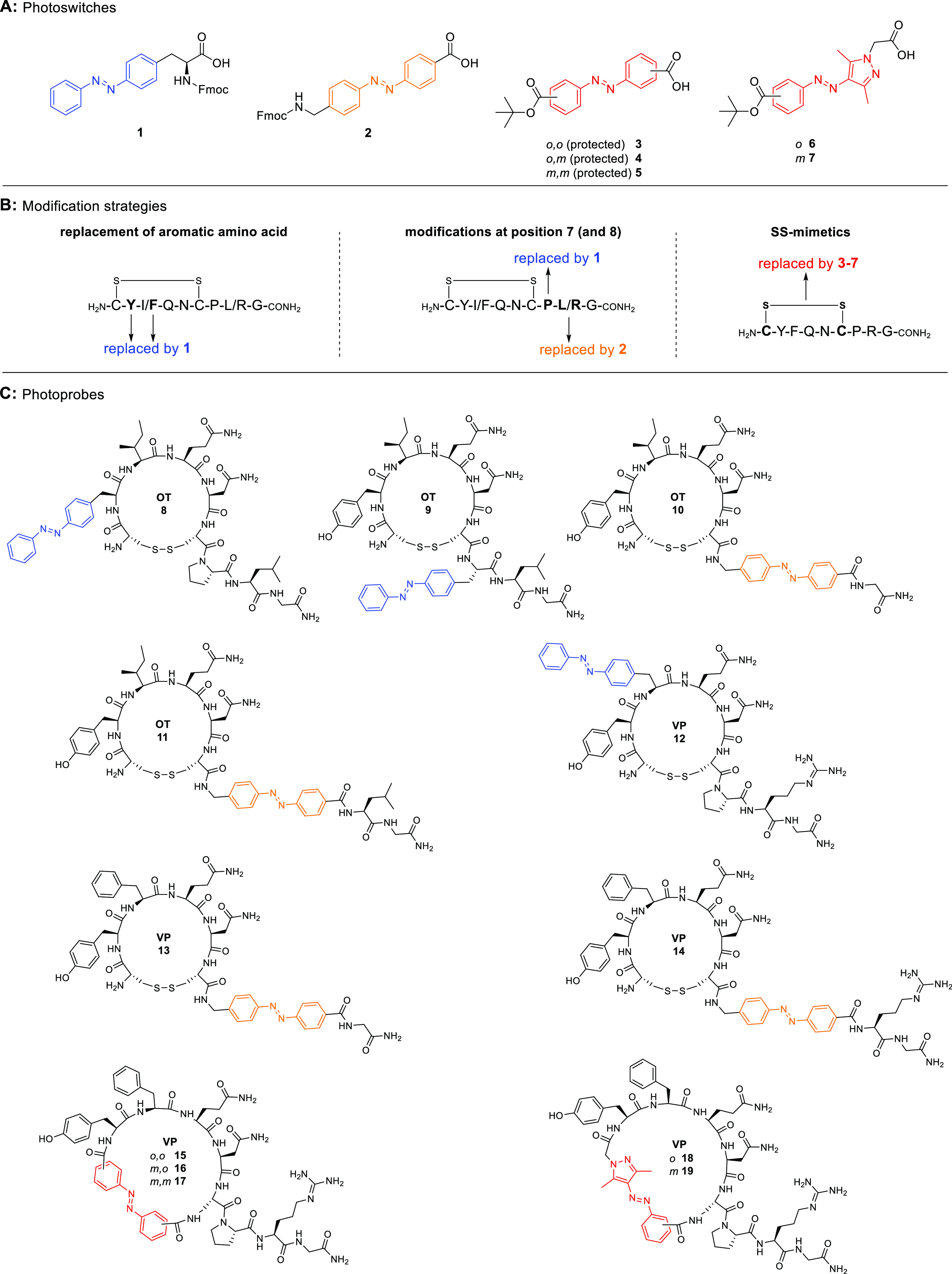
Overview of photoswitches and photoprobe design strategies. (A) Selected photochromic moieties for incorporation into OT and VP. (B) Different approaches for OT and VP modifications. (C) Structures of the synthesized photoswitchable OT (**8**–**11**) and VP (**12**–**19**) derivatives.

OT and VP are highly sensitive to structural modifications, often resulting in significant decreases in affinity and activity even with subtle changes, rendering it a challenging molecular class to work with.^24^ To overcome this challenge, we explored different modification strategies and photoswitch designs ranging from conservative to more drastic modifications. The designs included the replacement of an aromatic amino acid by photoswitchable amino acid, modifications in the exocyclic tail, and the most drastic approach, the replacement of the disulfide bond with a photoswitchable moiety (Figure 2).

In the first approach, we replaced the aromatic amino acid at position 2 in OT (Tyr^2^) and position 3 in VP (Phe^3^) with photoswitch **1**, resulting in peptides **8** and **12**, respectively. Photoswitch **1** contains the photoresponsive moiety in the side chain of the amino acid, which is expected to cause minimal disruption to the core structures of OT and VP.

In the second approach, we focused on modifying the C-terminal tail of OT and VP. We substituted Pro^7^ with photoswitch **1**, resulting in peptide **9** with a photoswitchable side chain. Additionally, we incorporated photoswitch **2** (*para*/*para*-substituted to maintain a linear tail conformation) into the peptide backbone by replacing Pro^7^ to allow for a more pronounced geometric change at the C-terminal tail, resulting in peptides **11** and **14**. To compensate for the larger size of the photoswitch compared to Pro^7^, we also removed residue 8 (Ile^8^ in OT, Arg^8^ in VP) in the probe design, leading to peptides **10** and **13**.

In the third approach, we targeted the disulfide bond and replaced it with a photoswitch. For this strategy, we focused specifically on VP since even small changes in ring size in OT result in a loss of affinity and activity at OTR.^54,55^ VP and VPRs are more tolerant to ring size or disulfide bond modifications, as demonstrated by the discovery of linear antagonists for the VPRs.^56–58^ We used azobenzenes **3**–**5** and arylazopyrazoles **6** and **7** with different substitution patterns to create a small library of “disulfide-mimetics” and ring sizes for VP. To minimize the increase in the ring size, we opted for *ortho* and *meta* substitutions. We also employed arylazopyrazoles due to their enhanced photophysical properties, including higher photostationary states (PSS) and longer thermal half-lives than azobenzenes.^59^

### Synthesis

**Scheme 1:**
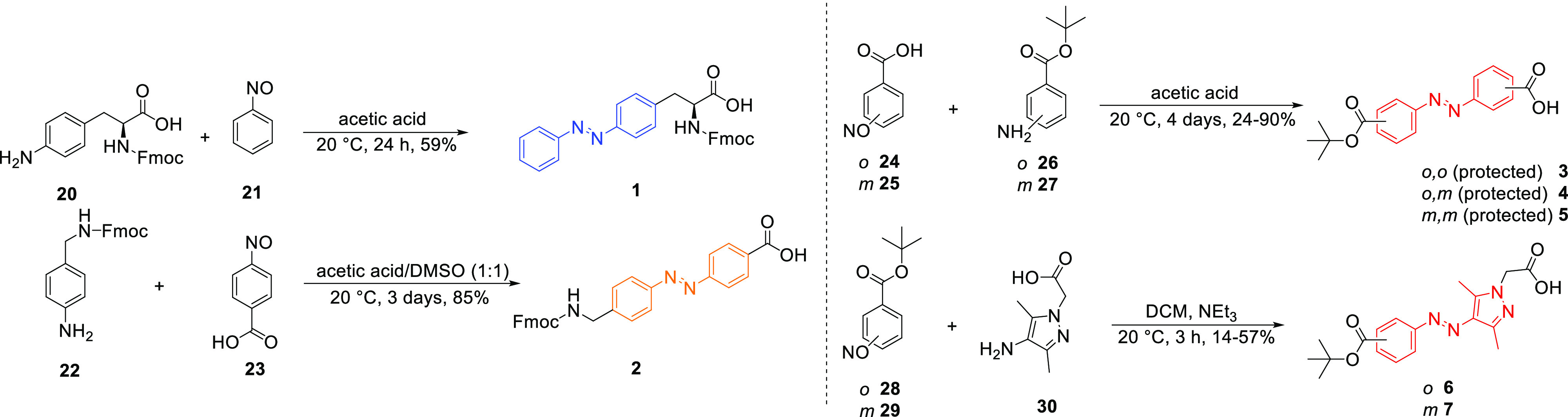
Synthetic Schemes to Produce Photoswitches **1–7**

Photoswitches **1** and **2** were synthesized using a Mills reaction (Scheme 1). Starting from amino-phenylalanine **20** and nitrosobenzene **21**, the photoswitchable derivative of Fmoc-protected phenylalanine **1** was synthesized to replace either Tyr^2^ or Pro^7^ in OT or Phe^3^ in VP.^60^ Photoswitch **2**, to replace Pro^7^ and to mimic the backbone, was also synthesized via a Mills reaction from **22** and **23**.^61^ Photoswitches **3**–**7**, containing a free and a protected carboxylic acid moiety, were synthesized to replace the disulfide bond. The required nitroso-compounds **24**, **25**, **28**, and **29** were produced from the corresponding amines in an oxidation reaction with oxone.^62^ The Mills reaction that afforded the azobenzenes **3**–**5** was done in acetic acid over 4 days. For preparing the arylazopyrazoles **6** and **7**, the Mills reaction was performed under basic conditions with triethylamine in dichloromethane (DCM).

**Scheme 2:**
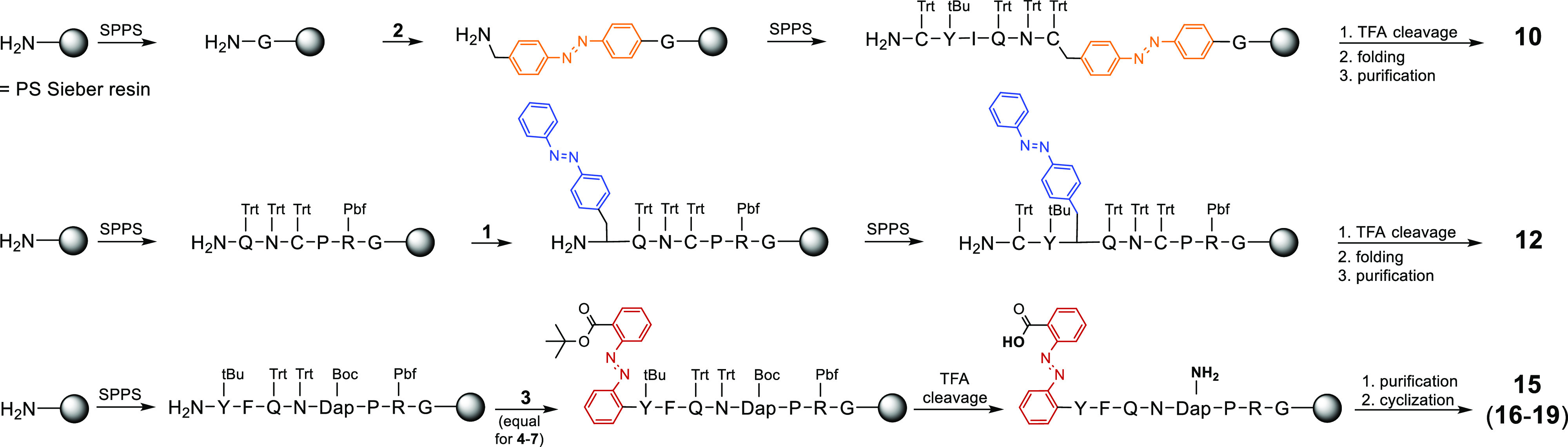
Synthetic Strategies to Generate the Designed OT/VP Photoprobes Reagents and conditions: SPPS: amino acid coupling: Fmoc-amino acid/HBTU/HOBt/DIPEA (5/5/4.9/10 equiv), DMF/NMP (8:2), 35 °C, 2 × 45 min (double coupling), Fmoc deprotection: 20% piperidine in DMF/NMP (8:2), 20 °C, 2 × 10 min; photoswitch **1** or **2**/HBTU/HOBt/DIPEA (3/3/2.95/6 equiv), DMF/NMP (8:2), 35 °C, 24 h (single coupling), Fmoc deprotection: 20% piperidine in DMF/NMP (8:2), 20 °C, 2 × 10 min; cleavage from resin and side chain deprotection: TFA/DCM/TIPS/H_2_O (50/46/2/2, v/v), 20 °C, 4–5 h; folding: MeCN/phosphate buffer (pH = 7.4) (7/3) + 10% DMSO, 20 °C, o/n; photoswitches **3** or **4**/DIC (3/3 equiv), DMF/NMP (8:2), 35 °C, 24 h (single coupling), photoswitches **5**–**7**/PyBOP/HOBt/DIPEA (3/3/3/6 equiv), DMF/NMP (8:2), 35 °C, 24 h (single coupling); purification on preparative HPLC; cyclization: PyBOP/HOBt/DIPEA (5/5/10 equiv), DMF/NMP (8:2), 20 °C, 24 h. Dap = diaminopropionic acid. Protecting groups: Trt = trityl, ^*t*^Bu = *tert*-butyl, Pbf = 2,2,4,6,7-pentamethyldihydrobenzofuran-5-sulfonyl, Boc = tert-butyloxycarbonyl. (For routes toward peptides **8**, **9**, **11**, **13**, and **14**, see Supporting Information, Scheme S1).

The peptide precursors were assembled via manual Fmoc-solid-phase peptide synthesis (Fmoc-SPPS) using a Sieber amide resin (selected routes, Scheme 2). The natural amino acids and photoswitches **1** and **2** were coupled using HBTU/HOBt/DIPEA in DMF/NMP (8:2 v/v). For photoswitches **5**–**7**, PyBOP/HOBt/DIPEA was used. However, the coupling of photoswitches **3** and **4** with *ortho*-substitution using PyBOP was not successful due to the immediate decomposition of the active ester. Therefore, *N*,*N*′-diisopropylcarbodiimide (DIC) was used, as it forms a different activated species (*O*-acylisourea intermediate). All natural amino acids were double-coupled with a 5-fold excess of HBTU/HOBt/DIPEA in DMF/NMP (8:2 v/v) for 45 min at 35 °C, while a single coupling using a 3-fold excess of HBTU/HOBt/DIPEA in DMF/NMP (8:2 v/v) was used for **1**–**7**, with an overnight reaction at 35 °C. After the final coupling step, the peptides were globally deprotected using TFA/DCM/triisopropylsilane (TIPS)/H_2_O (50/46/2/2, v/v) and cleaved from the resin at 20 °C for 4–5 h, followed by the addition of water and lyophilization.

Subsequently, the precursor peptides of **8**–**14** containing cysteine residues were folded using DMSO at 20 °C overnight as the oxidizing agent, followed by lyophilization and purification by preparative HPLC. The linear precursors of peptides **15**–**19**, which did not contain cysteine residues but instead had photoswitches, were purified by preparative HPLC and then cyclized in solution using PyBOP/HOBt/DIPEA at a peptide concentration of 5 mM in DMF/NMP (8:2 v/v). The cyclized peptides were purified by preparative C_18_-RP-HPLC.

The overall yields of **8**–**14** containing a disulfide bridge ranged between 2–5%, while the yields of the linear precursors of peptides **15**–**17** containing an azobenzene ranged between 8–17%. The precursors to peptides **18** and **19** with arylazopyrazole had the best yields 32 and 68%, respectively. The cyclization yields of **15**–**19** ranged between 23–60%. The lower yields can be explained due to partial reduction of the azo bond, likely by TIPS, which is used as a scavenger but can also act as a mild reducing agent. Removal of TIPS was not possible without detection of the trityl protecting groups by mass spectrometry. The higher yields for the arylazopyrazoles were due to the electron-rich pyrazole moiety, which prevented the reduction of the azo bond.^63^

### Photophysical Characterization

All photoprobes were investigated regarding their photophysical properties in aqueous buffer (pH 7.5), mimicking the conditions of the biological functional assays. UV/vis spectra displayed the characteristic absorptions of *E*- and *Z*-azobenzene.^44^ Upon irradiation with 340 nm light for 10 s and monitoring via UV/vis spectroscopy, all compounds could be switched to the *Z*-isomer, reaching the photostationary state (PSS). Compounds **8**–**17** containing azobenzene could be switched back to the *E*-isomer by irradiation at 420 nm for 60 s. Compounds **18** and **19** containing arylazopyrazole could be reverted to the *E* isomer by using 528 nm light. The switching process was repeated over 10 cycles, demonstrating high fatigue resistance of the compounds. The PSS, when switched to the *Z*-isomer, was consistently good, with a PSS of 81–95%. Switching back to the *E*-isomer also worked well for most compounds, except for peptides **8**, **9**, and **12**, which contained the photoswitchable phenylalanine and had a PSS below 80%, and peptide **15**, which contained the *ortho*, *ortho-*substituted azobenzene, and had a PSS of 69% (Table 1). Interestingly, peptide *E***-15** was slightly more polar than *Z***-15**, whereas the *Z*-isomers of all other peptides were slightly more polar (assessed by analytical C_18_-RP-HPLC). The thermal half-life, which describes the conversion rate from the metastable *Z*-isomer to the *E*-isomer, ranged from 12 h to 54 days at 25 °C (Table 1). The thermal half-lives of **10** and **12** were also measured at 37 °C, representing the temperature during the biological assays. As expected, the thermal half-lives decreased, however, the ratio of *E* and *Z* isomers remained stable during the incubation period of the biological assays (1 h).

**Table 1:** Summary of the Photophysical Properties of the Switchable Photoprobes **8**–**19**^a^

compound	λ_max_ (*E*) [nm]	λ_max_ (*Z*) [nm]	λ_iso_ [nm]	*t*_1/2_ [days]^b^	PSS (*E* → *Z*)^d^ (%)	PSS (*Z* → *E*)^d^ (%)
**8**	328	430	277, 395	5.5	94	72
**9**	330	428	281, 391	3.0	93	77
**10**	332	430	285, 393	1.7 (1.0^c^)	86	81
**11**	333	430	285, 396	1.2	87	85
**12**	330	430	281, 390	3.0 (1.4^c^)	95	74
**13**	332	425	285, 405	0.5	85	79
**14**	332	430	285, 400	1.4	81	86
**15**	320	440	277, 380	2.7	88	69
**16**	320	430	277, 390	3.1	89	83
**17**	323	425	274, 375	28.6	93	88
**18**	340	427	293, 403	6.6	94	82
**19**	333	431	285, 402	54.5	93	87

a Isomerization was obtained by irradiation with 340 nm (*Z*-isomer) and 420 nm for **8**–**17** and 528 nm for **18** and **19** (*E*-isomer), respectively. Concentration: 20 μM in HEPES buffer (25 mM HEPES, 2.5 mM CaCl_2_, 1 mM MgCl_2_, pH = 7.4) + 0.1% DMSO.

b Preirradiation with 340 nm, measured at 25 °C.

c Measured at 37 °C.

d Prior irradiation with 340 nm (*Z*-isomer) and 420 nm for **8**–**17** and 528 nm for **18** and **19** (*E*-isomer), respectively, for 2 min to ensure the ratio at the PSS. PSS determination was done by analytical C_18_-RP-HPLC at the appropriate isosbestic points.

**Figure 3:**
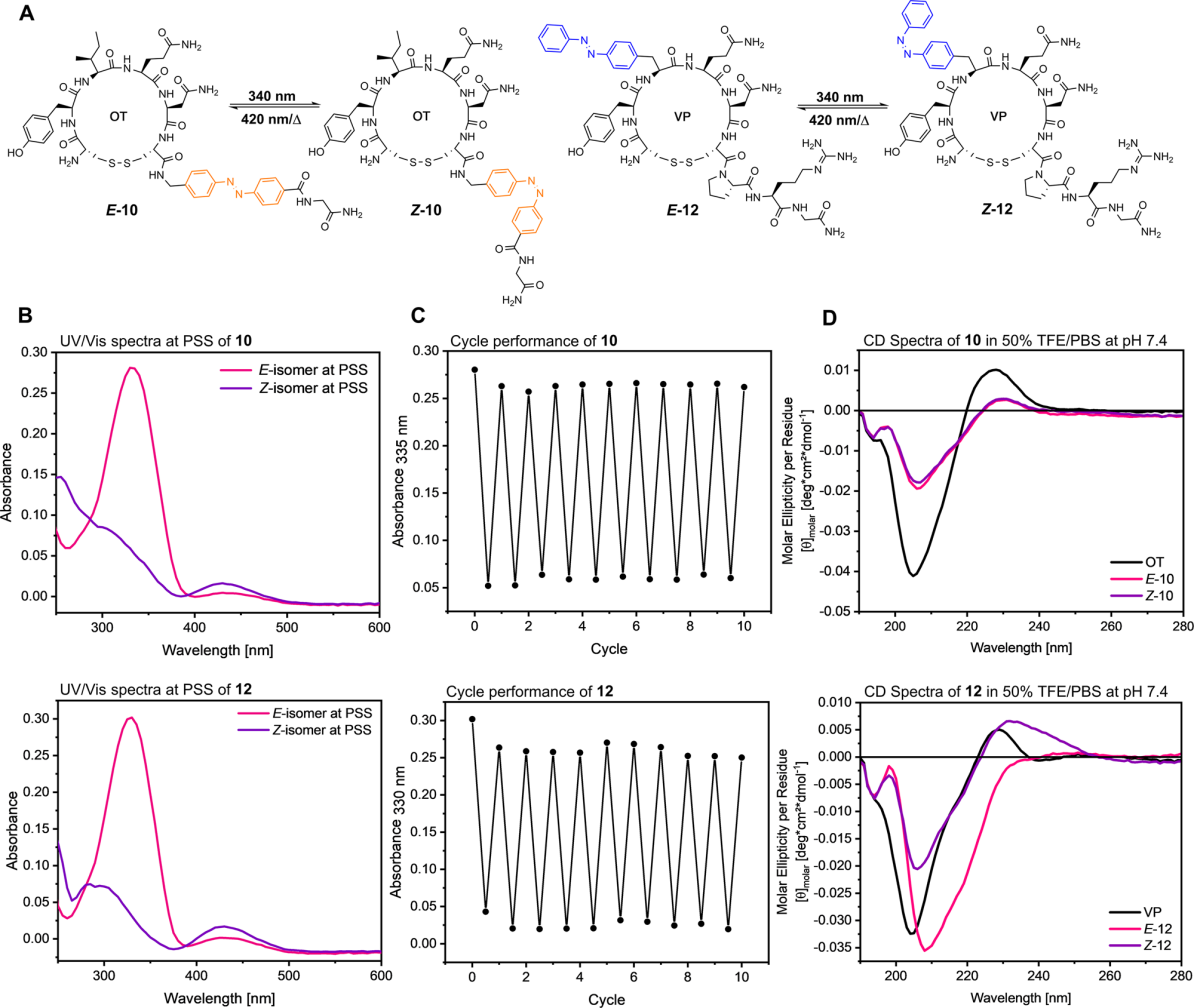
Representative chemical structures and photocharacterization of photoprobes **10** and **12**. (A) Chemical structures of light-induced *E*/*Z*-photoisomerization. (B) UV/vis spectra of *E*/*Z*-isomers at PSS. (C) Cycle performance. Results are of photoprobes **10** and **12** (20 μM) in HEPES buffer (25 mM HEPES, 2.5 mM CaCl_2_, 1 mM MgCl_2_, pH = 7.4) + 0.1% DMSO. Photoprobes **8**, **9**, **11** and **13**–**19** displayed similar photophysical properties (Figures S13–S24). (D) Circular dichroism (CD) spectra of the photoisomers of **10** and **12** and their respective parent peptides OT and VP (300 μM in 50% TFE/PBS, pH = 7.4).

Compounds **10** and **12** were further studied by circular dichroism (CD) spectroscopy to investigate potential changes in their secondary structures after photoswitching. Compounds **10** and **12** were chosen due to their promising bioactivity data, as described in the next section. 300 μM solutions of the compounds and their respective parent peptides were prepared in phosphate-buffered saline (PBS, pH 7.4) with varying amounts (0, 25, and 50%) of trifluoroethanol (TFE) to mimic the hydrophobic receptor binding pocket within the cell membrane. While no remarkable differences were observed for the *E*/*Z* isomers of **10**, presumably due to the location of the photoswitch in the flexible three-residue exocyclic tail, compound **12**, with the modification in the more constrained macrocyclic part, displayed observable structural changes after irradiation (Figures 3D and S47). This effect was especially pronounced in a more hydrophobic environment (50% TFE, Figure 3D), indicating a change in the secondary structure for *E-***12** in the binding pocket, supporting diverging activity between these two isomers.

### Biological Characterization

The photoswitchable OT/VP derivatives **8**–**19** were pharmacologically characterized at the human OTR, V_1a_R, or V_1b_R, using HEK293 cells overexpressing the respective receptor subtype and a commercially available IP-One accumulation assay (Cisbio) for G_q_-coupled receptors.^64^ V_2_R was not evaluated as it is primarily expressed in the kidneys and absent in the brain. Endogenous ligands OT (OTR) and VP (V_1a_R, V_1b_R) were used as reference compounds. The *Z*- and *E*-isomers of each photoswitchable peptide were measured separately after prior irradiation of the dilution series at the appropriate wavelength (Table 1). Initially, all compounds were measured at two concentrations (100 nM and 10 μM), followed by a full dose–response curve for any active compounds. In the following paragraphs, “*E*-isomer” refers to the ratio at the thermal equilibrium and “*Z*-isomer” to the ratio at the PSS after irradiation with 340 nm for 1 min.

Table 2 summarizes the EC_50_ and *E*_max_ values of the three modification strategies, including OT (**8**–**11**) and VP derivatives (**12**–**14**) at the human OTR, V_1a_R, or V_1b_R. We first report the impact of the photoswitch incorporation compared to OT and VP, followed by the differences between the *Z*- and *E*-isomers.

**Table 2:** OTR, V_1a_R, and V_1b_R Potencies (EC_50_) and Efficacies (*E*_max_) of OT and VP and **8**–**14**^a^

	OTR				V_1a_R			V_1b_R		
	EC_50_ ± SEM [nM]	*E*_max_ ± SEM [%]	*E*/*Z* ratio EC_50_		EC_50_ ± SEM [nM]	*E*_max_ ± SEM [%]	*E*/*Z* ratio EC_50_	EC_50_ ± SEM [nM]	*E*_max_ ± SEM [%]	*E*/*Z* ratio EC_50_
OT	2.46 ± 0.58	100		VP	0.64 ± 0.09	100		3.33 ± 0.48	100	
*E*-**8**	4501 ± 1014	45 ± 3	1.3	*E*-**12**	722 ± 53	104 ± 1	2.0	**107 ± 13**	**95 ± 1**	**5.3**
*Z*-**8**	3496 ± 1887	35 ± 1		*Z*-**12**	1439 ± 108	99 ± 1		**20.2 ± 6**	**95 ± 1**	
*E*-**9**	346 ± 30	89 ± 1	2.5	*E*-**13**	>10 μM			11,260 ± 1829	45 ± 3	1.6
*Z*-**9**	136 ± 6	90 ± 1		*Z*-**13**	>10 μM			7228 ± 1575	52 ± 5	
*E*-**10**	**94.6 ± 19**	**80 ± 1**	**3.1**	*E*-**14**	>10 μM			4967 ± 754	48 ± 2	2.1
*Z*-**10**	**30.7 ± 4**	**92 ± 0**		*Z*-**14**	>10 μM			2373 ± 607	67 ± 2	
*E*-**11**	263 ± 21	75 ± 1	2.0							
*Z*-**11**	134 ± 17	86 ± 1								

a Determined in an IP-One accumulation assay performed with stable HEK293 cell lines expressing hOTR, hV_1a_R, and hV_1b_R. Efficacies were determined relative to the effect of OT (OTR) and VP (VPRs). Data represent mean values from at least three independent experiments performed in triplicate [standard error of the mean (SEM)].

In the first strategy, we focused on the aromatic residues at positions 2 and 3 of the macrocycle. OT-based peptide **8**, with the photoswitch **1** incorporated at Tyr^2^, displayed a substantial decrease in potency compared to OT (>1400-fold) with an EC_50_ in the 3–4 μM range and partial agonism (*E*_max_ 35–45%). The cryo-electron microscopy structure of OTR in complex with OT confirmed that Tyr^2^ is located deep in the binding pocket,^65^ and the decrease in activity is thus likely due to the larger size of the azobenzene replacing Tyr^2^ or due to the removal of the hydroxyl group that can act as an H-donor.^66^ Displacement of Phe^3^ in VP with photoswitch **1** (peptide **12**) also had reduced potency at V_1a_R (>1000-fold compared to VP), but at V_1b_R it remained potent (*Z***-12**, EC_50_ 20.2 nM, full agonist, 6-fold less potent than VP), supporting the note that V_1b_R is more tolerant to modifications than V_1a_R.

The second modification strategy focused on the three-residue C-terminal tail. The OT-OTR structure shows that the C-terminal tail points outside the binding pocket toward the extracellular loops, suggesting more structural space for modifications. OT-derived peptides **9**, **10**, and **11** were modified at Pro^7^ by either substitution with photoswitch **1** (peptide **9**) or linear backbone incorporation with photoswitch **2** (peptides **10** and **11**). This modification strategy was indeed better tolerated, resulting in only a 12-fold potency decrease for *Z***-10** and a 55-fold decrease for *Z***-9** and *Z***-11** compared to OT at the OTR. The *E*_max_ values ranged between 75–92%, supporting agonism for all these ligands in the IP-One assay. Linear photoswitch **2** was also introduced into the backbone of the three-residue tail of VP (peptides **13** and **14**). This, however, resulted in a complete loss of potency at V_1a_R at concentrations up to 10 μM (Figure S51) and a 700–2000-fold reduced potency at V_1b_R. The reason for diminished activity at VPRs is likely due to the removal of the positively charged Arg^8^ (**13**) or its shift toward the C-terminus (**14**); Arg^8^ is important for the formation of ionic interactions with the receptors.^67,68^

The third modification strategy focused on the disulfide bond of VP. The series of disulfide-mimetics **15**–**19** resulted in a complete loss of activity at both V_1a_R and V_1b_R (Figures S52 and S53), indicating that the azobenzene and arylazopyrazole motifs used in this study are too big and rigid to effectively mimic the disulfide bond.

**Figure 4:**
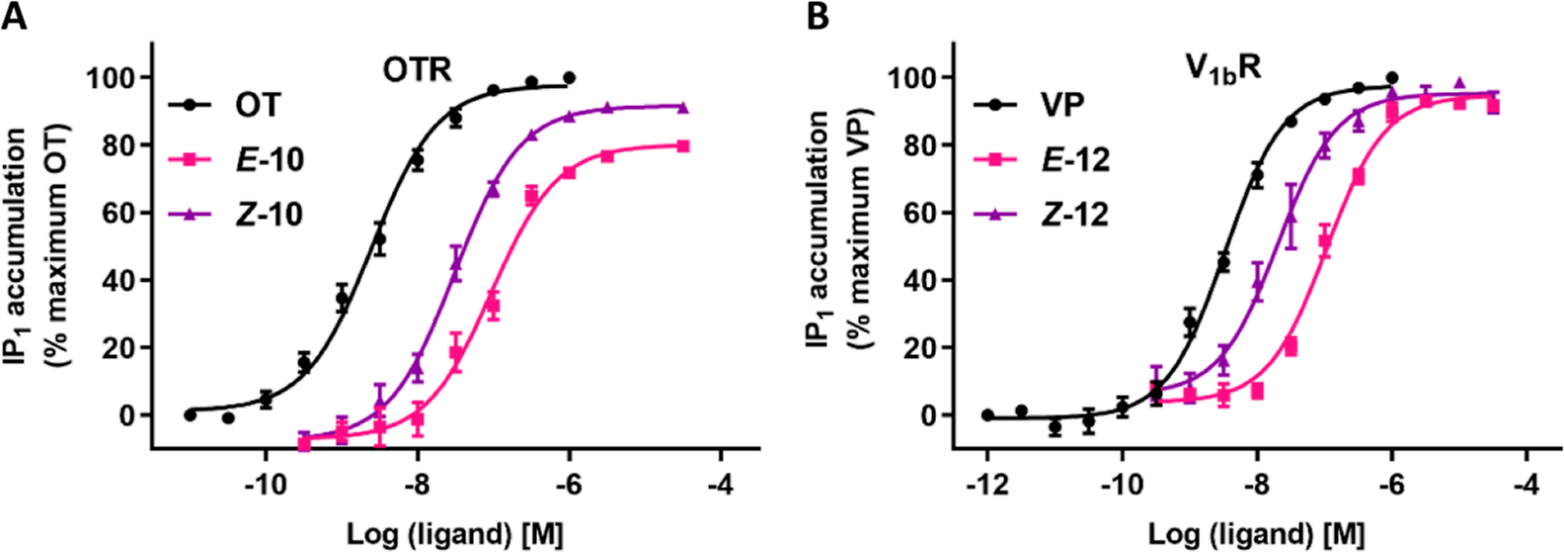
Full-dose–response curves of OT-derived lead photoprobe **10** at OTR (A) and VP-derived lead photoprobe **12** at V_1b_R (B). For full-dose–response curves of **8**, **9**, and **11**–**14**, please see Figure S50. Data represent mean values ± SEM from at least three independent experiments performed in triplicate.

The most promising candidates with a good activation ability were peptides **9**, **10**, and **11** at OTR and **12** at V_1b_R. Peptides **9**, **10**, and **11** were derived from the second modification strategy (substituting Pro^7^), and peptide **12** from the first modification strategy (substituting Phe^3^). Substituting Pro^7^ with the azobenzene photoswitch **1** in the side chain position (**9**) resulted in a full agonist with an EC_50_ of 136 nM for *Z***-9** and 346 nM for *E***-9** at OTR, exhibiting a 2.5-fold potency difference between the two isomers. Peptides **10** and **11**, which incorporated the photoswitch within the backbone instead of Pro^7^, were both potent agonists. Peptide **10**, where Leu^8^ was removed, displayed a 3.1-fold potency difference between the two isomers, with an EC_50_ of 94.6 nM for *E***-10** and 30.7 nM for *Z***-10** (Figure 4). Peptide **11**, the longer derivative with Leu^8^, displayed a 2-fold potency difference, with EC_50_ values of 263 nM for *E***-11** and 134 nM for *Z***-11**. Peptide **10** was, therefore, the most promising candidate from the OT-derived series at OTR (Figure 4A). Peptide **12**, featuring the azobenzene moiety in the side chain instead of Phe^3^, was the most promising candidate from the VP-derived series at V_1b_R, displaying a 5.3-fold potency difference, with EC_50_ values of 107 nM for *E***-12** and 20.2 nM for *Z***-12** (Figure 4B). *Z***-12** also displayed an ∼70-fold selectivity for V_1b_R over V_1a_R (Table 2) and a >500-fold selectivity over OTR (Figure S54), making it the most interesting photoprobe of this series.

Because the structural changes could result in an agonist-to-antagonist switch, we tested all inactive compounds for their ability to antagonize V_1a_R, representing the most important VPR. This was done via coaddition and prior mixing of VP (EC_60_ of 1 nM) and the potential antagonists **13**–**19**. The V_1a_R antagonist atosiban was used as a positive control.^21^ IP1 accumulation was measured at concentrations of 10 and 100 μM for peptides **13**–**19**.

**Figure 5:**
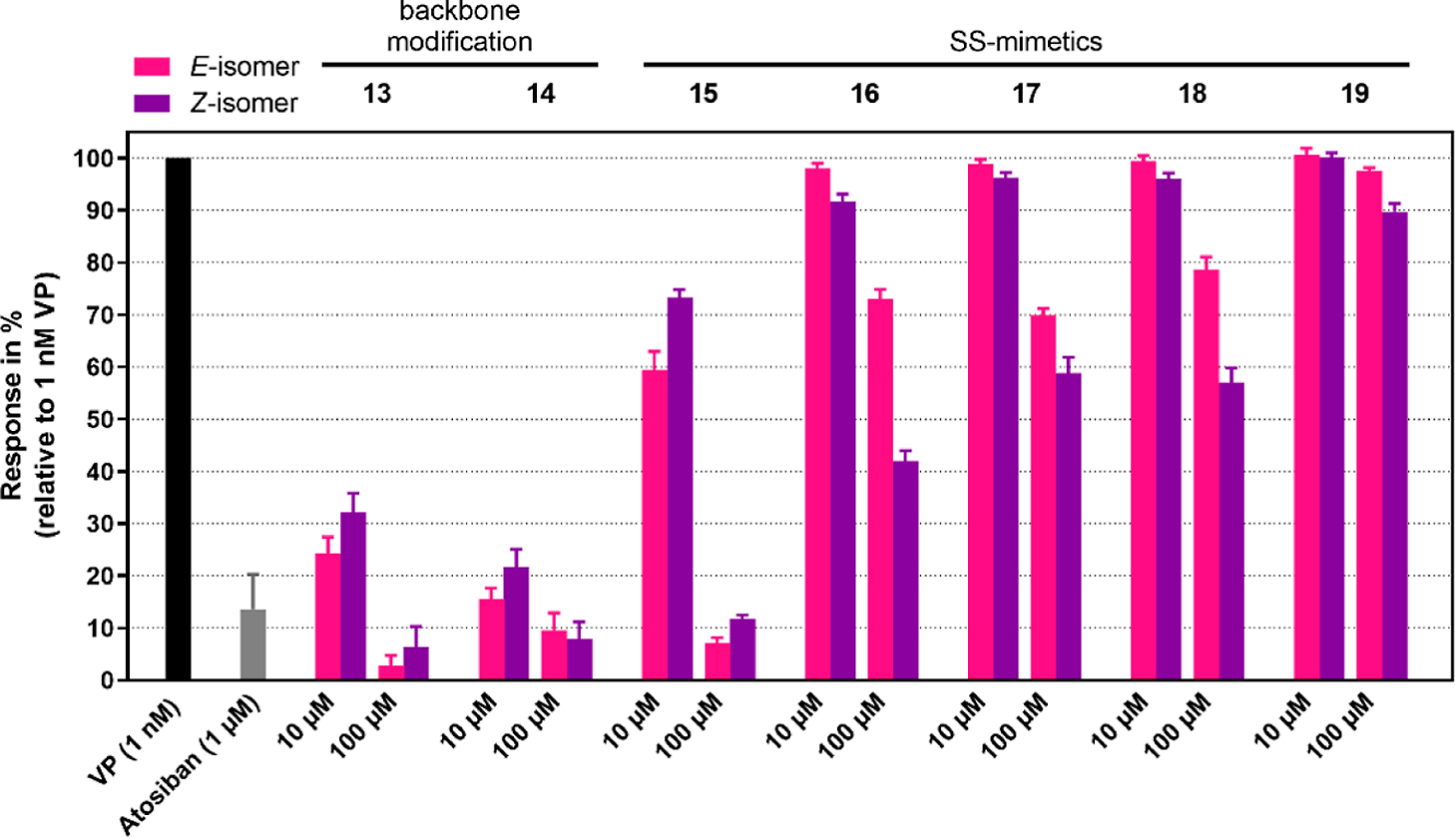
Functional screen for antagonistic activities at V_1a_R. VP displacement of positive control atosiban and **13**–**19** was determined in an IP-One accumulation assay with stable HEK293 cell lines overexpressing hV_1a_R by coaddition (premixed) of VP (1 nM) and atosiban (1 μM) or potential antagonists **13**–**19** (10 and 100 μM). Data represent mean ± SEM from at least three independent experiments, each performed in triplicate.

Peptides **13** and **14** exhibited some level of antagonistic activity, with VP displacements of 76 and 85%, respectively, at 10 μM. However, their antagonistic activity was relatively weak compared to atosiban, which achieved 86% VP displacement at 1 μM. The disulfide-mimetics (**16**–**19**) were mostly inactive at these concentrations (Figure 5). Only disulfide-mimetic **15**, with the *ortho*/*ortho* azobenzene (smallest ring structure), displayed some antagonistic properties at 10 μM.

### In Vitro Immunostaining and Imaging by Confocal Scanning Microscopy

The most promising photoprobe, **12**, with a 5.3-fold photoswitch activity window at V_1b_R, was selected for more comprehensive in vitro experiments to validate its application scope for biological and neurological studies. As a model system, we used HEK293 cells overexpressing GFP-tagged V_1b_R, where receptor activation via pCREB (phosphorylated cAMP response element binding protein) and V_1b_R-GFP internalization can be followed through immunostaining and confocal scanning microscopy. Photoprobes *E-***12** and *Z-***12** were incubated for 1 h with these HEK293 cells, followed by immunostaining with primary and secondary antibodies for GFP and pCREB and imaging by confocal scanning microscopy.

**Figure 6:**
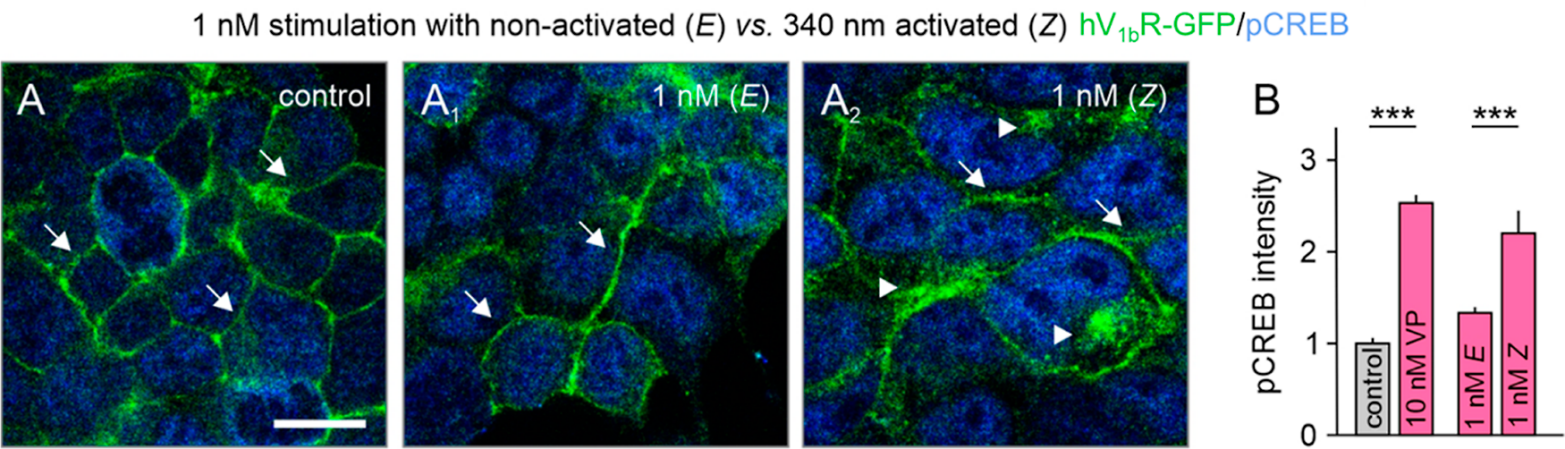
Evaluation of V_1b_R photoprobe **12** in HEK293 cells overexpressing V_1b_R-GFP using immunostainings and confocal scanning microscopy. (A–A_2_) Stimulation of HEK293 cells overexpressing GFP-conjugated human V_1b_R. Nonactivated *E*-**12** (1 nM) did not induce V_1b_R-GFP internalization (arrows, green GFP signals remain at cell membranes, similar to control). By contrast, 340 nm activated *Z*-**12** induced V_1b_R-GFP internalization at 1 nM (arrowheads: green GFP signals move into cytosol). (B) Photoprobe *Z*-**12** significantly induced CREB phosphorylation (blue) at 1 nM, similar to the positive control VP at 10 nM. *P* < 0.001. Scale bar = 15 μm.

Photoprobe *E***-12** (EC_50_ = 107 nM) did not cause any visible receptor activation or internalization at 1 nM (Figure 6A,A_1_), similar to the control (medium). By contrast, photoactivated *Z***-12** (EC_50_ = 20.2 nM) activated V_1b_R-GFP at 1 nM, resulting in β-arrestin recruitment and V_1b_R-GFP internalization (Figure 6A_2_). Moreover, *Z***-12** significantly (*P* < 0.001) activated pCREB at levels comparable to VP (10 nM, positive control), showing clear differences between inactive *E***-12** and negative control (Figure 6A,A_2_,B). These results support the notion that the 5-fold photoswitch window for the two isomers of compound **12** is sufficient to selectively activate V_1b_R, rendering it a promising new photopharmacological tool for the spatiotemporal investigation of this receptor.

Given the involvement of OT, VP, and their receptors in central disorders, there is considerable interest in photocontrollable ligands to better dissect the OT/VP-dependent neuronal circuits of the brain with high spatiotemporal precision as subtype receptor density is especially high in the brain tissue but also the selective activation of receptors in the mammary gland or the uterine gland are conceivable areas of application for our photoprobes.^23,33^ In vivo applications in the brain remain challenging, but these photoprobes can be highly valuable for further in vitro and ex vivo applications, e.g., in organotypic brain slices.^69^ To address this gap, we explored different photoswitchable moieties and OT and VP modification strategies, resulting in the first photoswitchable OT and VP derivatives. Compounds **10** and **12** are promising photoprobe leads for OTR and V_1b_R, exhibiting a multifold activity difference between the less active *E* and more active *Z* isomers and only slightly reduced potency compared to the endogenous ligands. OT-derived compound **10** had a 3-fold difference in potency between the two isoforms at the OTR, and VP-derived compound **12** had a 5-fold difference at the V_1b_R. Although these compounds do not represent a full on/off-switch, they enable selective light-induced activation of the target receptor at specific concentrations. For example, *Z***-12** activated V_1b_R at 1 nM, similarly to VP, whereas *E***-12** was inactive at the same concentration. These differences in photoactivation profiles render these compounds intriguing leads for subsequent ex vivo assays or further optimization to extend that photoactivation window or achieve full switch on/off capabilities. Moreover, the structure–activity relationships derived from this work provide insights and guidance for new modifications to improve photopharmacological capabilities. Future alterations, for example, could involve changing the photochromic moiety to arylazopyrazoles, exploring different substituents and varying the substitution patterns on the phenyl rings. This study’s findings are expected to pave the way for generating more cutting-edge photocontrollable peptides that will facilitate the study of the dynamic and kinetic processes of OT and VP receptors as well as other peptide target receptors.

## Conclusions

We successfully developed the first photoswitchable OT- and VP-derivatives by incorporating photochromic moieties into different sites of their structures. All compounds exhibited good photochemical properties, including reversible switching, high fatigue resistance, good PSS, and thermal stability suitable for biological investigations. Not all design strategies worked, highlighting the sensitivity of OT and VP to structural modifications. Nevertheless, we identified peptide **10** as a promising photoprobe lead with a nanomolar potency at OTR and a 3.1-fold difference between the *E* and *Z* isomers. VP-derived peptide **12** also shows high potential, with only 6-fold reduced potency compared to VP and a 5.3-fold difference between the *E* and *Z* isomers at V_1b_R. The application potential of photoprobe **12** was validated in vitro using confocal scanning microscopy, demonstrating that V_1b_R can be selectively activated by photoswitched *Z***-12** but not *E***-12**. The findings provide valuable new insights into receptor tolerance for structural modifications and serve as a foundation for designing a new generation of photoswitchable OT and VP derivatives.

## Experimental Section

### General

Starting materials and commercial reagents were purchased from Acros, Alfa Aesar, Fisher, Fluka, Fluorochem, Merck (Sigma-Aldrich), TCI and VWR and were used without further purification. Solvents were used in p.a. quality or dried according to common procedures if necessary. All reactions with oxygen- or moisture-sensitive reagents were carried out in glassware that was dried before use by heating under vacuum. Dry nitrogen or argon was used for an inert gas atmosphere. All NMR spectra were measured at room temperature (20 °C) using a Bruker Avance 400 (400 MHz for ^1^H and 101 MHz for ^13^C) or a Bruker Avance 600 (600 MHz for ^1^H and 151 MHz for ^13^C) NMR spectrometer. All chemical shifts are reported in δ-scale as parts per million [ppm] (multiplicity, coupling constant *J*, number of protons) relative to the solvent residual peaks. Coupling constants *J* are given in hertz [Hz]. Abbreviations used for signal multiplicity: ^1^H NMR: s = singlet, d = doublet, dd = doublet of doublets, ddd = doublet of doublets of doublets, dt = doublet of triplets, t = triplet, td = triplet of doublets, q = quartet, and m = multiplet. All mass spectra were recorded on an Agilent Q-TOF 6540 UHD, Finnigan MAT SSQ 710 A, Jeol AccuTOF GCX or ThermoQuest Finnigan TSQ 7000 spectrometer. Absorption spectra were recorded on an UV/vis Agilent Cary 50 spectrometer. Thermal half-lives were measured on a 96-well plate in a Thermo Scientific Multiskan Spectrum. Analytical TLC was performed on silica gel-coated alumina plates (MN precoated TLC-sheets ALUGRAM Xtra SIL G/UV254). Visualization was done using UV light (254 or 366 nm) or staining with ninhydrin solution. Column chromatography was performed on a Biotage Isolera One automated flash purification system with a UV/vis detector. Analytical RP-HPLC was carried out on an Agilent 1220 Infinity LC System (column: P/No 00F-4251-B0, Phenomenex Luna 3 μm C_18(2)_ 100 Å, LC column 150 mm × 2.0 mm). Purification by preparative HPLC was conducted on a preparative HPLC Agilent 1260 Infinity LC System [column: P/No 00G-4253-P0-AX, Phenomenex Luna 10 μm C_18(2)_ 100 Å, LC column 250 mm × 21.2 mm]. The eluent systems were used as specified. After the purification process, solvents were removed by lyophilization. Switching experiments were done with a 340 nm LED (SSC VIOSYS CUD4AF1B, 500 mA, 55 mW), 420 nm LED (ILH-XC01-S410-SC211-WIR200, 800 mA, 440 mW), and 528 nm LED (OSRAM Oslon SSL 80 green, 500 mA, 34 mW). Purity of all photoprobes was >95% as determined by analytical RP-HPLC at 220 nm in DMSO.

### General Procedure for the Synthesis of Azobenzenes **3–5**

Nitroso **24** or **25** (1.0 equiv) and amine **26** or **27** (1.0 equiv) were dissolved in AcOH and stirred at 20 °C for 4 days. The solvent was removed in vacuo, and the crude product was purified by column chromatography (MeCN/EtOH, 0–30%).

### (*E*)-2-((2-(*tert*-Butoxycarbonyl)phenyl)diazenyl)benzoic Acid (**3**)

Yield: 30%. ^1^H NMR (400 MHz, DMSO-*d*_6_): δ 7.82 (dd, *J* = 7.6, 1.5 Hz, 1H), 7.75 (dd, *J* = 7.5, 1.5 Hz, 1H), 7.70 (tdd, *J* = 7.5, 5.7, 1.6 Hz, 2H), 7.62 (tdd, *J* = 7.5, 3.6, 1.3 Hz, 2H), 7.48 (ddd, *J* = 7.9, 4.9, 1.2 Hz, 2H), 1.45 (s, 9H). ^13^C NMR (101 MHz, DMSO-*d*_6_): δ: 168.1, 166.1, 150.9, 150.9, 131.9, 131.9, 131.0, 130.9, 130.8, 129.4, 129.2, 118.3, 117.6, 81.9, 27.8. ESI-MS *m*/*z* (%) = 327.13 (M + H)^+^.

### (*E*)-2-((3-(*tert*-Butoxycarbonyl)phenyl)diazenyl)benzoic Acid (**4**)

Yield: 90%. ^1^H NMR (400 MHz, DMSO-*d*_6_): δ 8.31 (t, *J* = 1.9 Hz, 1H), 8.09 (ddt, *J* = 6.9, 4.2, 1.8 Hz, 2H), 7.82 (dd, *J* = 7.5, 1.5 Hz, 1H), 7.74 (t, *J* = 7.8 Hz, 1H), 7.68 (dd, *J* = 7.6, 1.6 Hz, 1H), 7.64–7.57 (m, 2H), 1.58 (s, 9H). ^13^C NMR (101 MHz, DMSO-*d*_6_): δ: 168.2, 164.3, 152.0, 150.7, 132.7, 131.8, 131.8, 130.6, 130.0, 129.4, 127.0, 122.8, 118.2, 81.5, 27.8. ESI-MS *m*/*z* (%) = 327.13 (M + H)^+^.

### (*E*)-3-((3-(*tert*-Butoxycarbonyl)phenyl)diazenyl)benzoic Acid (**5**)

Yield: 24%. ^1^H NMR (400 MHz, DMSO-*d*_6_): δ: 8.40 (d, *J* = 1.9 Hz, 1H), 8.36 (d, *J* = 1.9 Hz, 1H), 8.21–8.06 (m, 4H), 7.74 (td, *J* = 7.8, 2.0 Hz, 2H), 1.58 (s, 9H). ^13^C NMR (101 MHz, DMSO-*d*_6_): δ: 166.7, 164.2, 151.7, 132.7, 132.5, 132.2, 131.9, 130.0, 130.0, 127.4, 126.9, 122.7, 122.5, 81.5, 27.8. ESI-MS *m*/*z* (%) = 327.14 (M + H)^+^.

### General Procedure for the Synthesis of Arylazopyrazoles **6** and **7**

Compound **28** or **29** (1.0 equiv) in DCM was added to (4-amino-3,5-dimethyl-1*H*-pyrazol-1-yl)acetic acid dihydrochloride hydrate (**30**) (1.0 equiv) dissolved in DCM. NEt_3_ was then added dropwise until the solution turned yellow. The mixture was stirred for 3 h at 20 °C. The solvent was removed in vacuo, and the crude product was purified by column chromatography (MeCN/EtOH, 0–30%).

### (*E*)-2-(4-((2-(*tert*-Butoxycarbonyl)phenyl)diazenyl)-3,5-dimethyl-1*H*-pyrazol-1-yl)acetic Acid (**6**)

Yield: 14%. ^1^H NMR (400 MHz, DMSO-*d*_6_): δ 7.59 (ddd, *J* = 15.3, 7.4, 1.5 Hz, 2H), 7.51 (dd, *J* = 8.1, 1.3 Hz, 1H), 7.45 (td, *J* = 7.4, 1.4 Hz, 1H), 4.95 (s, 2H), 2.48 (s, 3H), 2.33 (s, 3H), 1.46 (s, 9H). ^13^C NMR (101 MHz, DMSO-*d*_6_): δ: 169.2, 166.6, 151.6, 141.4, 141.2, 135.2, 131.3, 130.6, 128.8, 128.8, 117.7, 81.4, 50.6, 27.8, 13.7, 9.5. ESI-MS *m*/*z* (%) = 359.17 (M + H)^+^.

### (*E*)-2-(4-((3-(*tert*-Butoxycarbonyl)phenyl)diazenyl)-3,5-dimethyl-1*H*-pyrazol-1-yl)acetic Acid (**7**)

Yield: 57%. ^1^H NMR (400 MHz, DMSO-*d*_6_): δ 8.19 (t, *J* = 1.8 Hz, 1H), 7.95 (ddt, *J* = 9.7, 7.8, 1.2 Hz, 2H), 7.63 (t, *J* = 7.8 Hz, 1H), 4.87 (s, 2H), 2.52 (s, 3H), 2.39 (s, 3H), 1.57 (s, 9H). ^**13**^C NMR (101 MHz, DMSO-*d*_6_): δ: 169.2, 164.5, 153.0, 141.2, 140.8, 134.6, 132.4, 129.6, 129.6, 125.4, 121.8, 81.1, 51.2, 27.8, 13.9, 9.5. ESI-MS *m*/*z* (%) = 359.17 (M + H)^+^.

### General Procedure for Peptide Synthesis

Peptides were synthesized by manual SPPS according to the Fmoc strategy using a Fmoc-PS-Sieber Resin. 5 mL Discardit-II syringes equipped with polyethylene frits were used as reaction vessels. DMF/NMP (8:2 v/v) was used as the solvent for the coupling reactions and the cleavage of Fmoc groups. For initial Fmoc deprotection of the resin and swelling, the resin was treated with 20% piperidine in a solvent at 20 °C for 2 × 20 min. Protected natural l-amino acids were used in 5-fold excess and preactivated with HBTU (4.9 equiv)/HOBt (5 equiv)/DIPEA (10 equiv) in polypropylene reaction vessels for 1 min prior to addition to the resin (volume of the solvent: ca. 2.2 mL/mmol Fmoc-amino acid). In the case of standard Fmoc-amino acids, “double” coupling (2 × 40 min) was performed at 35 °C. Photoswitches **1** and **2** were used in 3-fold excess, preactivated with HBTU (2.95 equiv)/HOBt (3 equiv)/DIPEA (6 equiv) (volume of solvent: ca. 1.6 mL/mmol of amino acid), and the reaction was performed at 35 °C overnight (“single” coupling). During coupling reactions, syringes were shaken using HLC BlockThermostate and ThermoMixer. After completion of coupling of Fmoc-amino acid, the resin was washed with solvents (4 × 5 mL) and treated with 20% piperidine in DMF/NMP (8:2 v/v) at 20 °C for 2 × 10 min followed by washing the resin with solvents (6 × 5 mL).

For the peptides containing cysteines **8**–**14**, the last step was the Fmoc deprotection of the last cysteine residue. For the linear precursors of peptides containing a photoswitch in the cyclic part **15**–**19**, after the final Fmoc deprotection of the final amino acid photoswitches **5**–**7** were preactivated with PyBOP (3 equiv)/HOBt (3 equiv)/DIPEA (6 equiv) and photoswitches **3** and **4** were preactivated with DIC (3 equiv), all photoswitches were used in 3-fold excess and were added to the resin and the reaction proceeded at 35 °C overnight.

Afterward, for all peptides, the resin was washed with the solvent (6×) and CH_2_Cl_2_ (4×), followed by TFA cleavage off the resin and global deprotection of the amino acid side chains with TFA/CH_2_Cl_2_/TIPS/H_2_O (50/46/2/2, v/v) at 20 °C for 4–5 h. The collected liquid phases were combined in a round-bottomed flask and water was added, followed by lyophilization and subsequent purification of the linear precursors of peptides **15**–**19** by preparative HPLC [column: Phenomenex Luna 10 μm C_18(2)_ 100 Å, 250 × 21 mm; flow: 22 mL/min, solvent A: H_2_O (0.05% TFA), solvent B: MeCN; and gradient A/B: 0–20 min: 97/3, 20–25 min: 2/98] and lyophilization.

For the peptides containing cysteines **8**–**14**, oxidative folding was carried out after lyophilization in MeCN/phosphate buffer (pH 7.4) and 10% DMSO at 20 °C overnight and subsequent purification by preparative HPLC [column: Phenomenex Luna 10 μm C_18(2)_ 100 Å, 250 × 21 mm; flow: 22 mL/min, solvent A: H_2_O (0.05% TFA), solvent B: MeCN; and gradient A/B: 0–20 min: 97/3, 20–25 min: 2/98]. The overall yield was calculated based on the resin used and its loading.

### [AzbS^2^]-OT (**8**)

Overall yield: 4%. HPLC (gradient: 0–15 min: MeCN/H_2_O + 0.05% TFA 10/90–98/2, 15–20 min: 98/2): *t*_R_: *Z*-isomer: 8.5 min, *E*-isomer: 9.4 min. HR-MS (ESI) calcd *m*/*z* for C_49_H_70_N_14_O_11_S_2_ (M + 2H)^2+^, 548.2468; observed, 548.2479.

### [AzbS^7^]-OT (**9**)

Overall yield: 3%. HPLC (gradient: 0–15 min: MeCN/H_2_O + 0.05% TFA 10/90–98/2, 15–20 min: 98/2): *t*_R_: *Z*-isomer: 8.9 min, *E*-isomer: 9.8 min. HR-MS (ESI) calcd *m*/*z* for C_53_H_72_N_14_O_12_S_2_ (M + H)^+^, 1161.4968; observed, 1161.4972.

### [AzbL^7/8^]-OT (**10**)

Overall yield: 2%. HPLC (gradient: 0–15 min: MeCN/H_2_O + 0.05% TFA 10/90–98/2, 15–20 min: 98/2): *t*_R_: *Z*-isomer: 7.6 min, *E*-isomer: 7.9 min. HR-MS (ESI) calcd *m*/*z* for C_46_H_59_N_13_O_11_S_2_ (M + 2H)^2+^, 517.7022; observed, 517.7028.

### [AzbL^7^]-OT (**11**)

Overall yield: 3%. HPLC (gradient: 0–15 min: MeCN/H_2_O + 0.05% TFA 10/90–98/2, 15–20 min: 98/2): *t*_R_: *Z*-isomer: 8.5 min, *E*-isomer: 8.9 min. HR-MS (ESI) calcd *m*/*z* for C_52_H_70_N_14_O_12_S_2_ (M + 2H)^2+^, 574.2442; observed, 574.2451.

### [AzbS^3^]-VP (**12**)

Overall yield: 5%. HPLC (gradient: 0–15 min: MeCN/H_2_O + 0.05% TFA 10/90–98/2, 15–20 min: 98/2): *t*_R_: *Z*-isomer: 7.3 min, *E*-isomer: 8.3 min. HR-MS (ESI) calcd *m*/*z* for C_52_H_69_N_17_O_12_S_2_ (M + 2H)^2+^, 594.7449; observed, 594.7461.

### [AzbL^7/8^]-VP (**13**)

Overall yield: 5%. HPLC (gradient: 0–15 min: MeCN/H_2_O + 0.05% TFA 10/90–98/2, 15–20 min: 98/2): *t*_R_: *Z*-isomer: 7.9 min, *E*-isomer: 8.0 min. HR-MS (ESI) calcd *m*/*z* for C_49_H_57_N_13_O_11_S_2_ (M + 2H)^2+^, 534.6944; observed, 534.6954.

### [AzbL^7^]-VP (**14**)

Overall yield: 4%. HPLC (gradient: 0–15 min: MeCN/H_2_O + 0.05% TFA 10/90–98/2, 15–20 min: 98/2): *t*_R_: *Z*-isomer: 7.4 min, *E*-isomer: 7.5 min. HR-MS (ESI) calcd *m*/*z* for C_55_H_69_N_17_O_12_S_2_ (M + 2H)^2+^: 612.7449; observed: 612.7458.

### Open[AzbC(*o*,*o*)^1^,Dap^6^]-VP (Linear Precursor to **15**)

Overall yield: 8%. HR-MS (ESI) calcd *m*/*z* for C_57_H_71_N_17_O_14_ (M + 2H)^2+^, 609.7756; observed, 609.7766.

### Open[AzbC(*m*,*o*)^1^,Dap^6^]-VP (Linear Precursor to **16**)

Overall yield: 17%. HR-MS (ESI) calcd *m*/*z* for C_57_H_71_N_17_O_14_ (M + 2H)^2+^, 609.7756; observed, 609.776.

### Open[AzbC(*m*,*m*)^1^,Dap^6^]-VP (Linear Precursor to **17**)

Overall yield: 14%. HR-MS (ESI) calcd *m*/*z* for C_57_H_71_N_17_O_14_ (M + 2H)^2+^, 609.7756; observed, 609.7763.

### Open[AzpC(*o*)^1^,Dap^6^]-VP (Linear Precursor to **18**)

Overall yield: 32%. HR-MS (ESI) calcd *m*/*z* for C_57_H_75_N_19_O_14_ (M + 2H)^2+^, 625.7943; observed, 625.7956.

### Open[AzpC(*m*)^1^,Dap^6^]-VP (Linear Precursor to **19**)

Overall yield: 68%. HR-MS (ESI) calcd *m*/*z* for C_57_H_75_N_19_O_14_ (M + 2H)^2+^, 625.7943; observed, 625.7952.

### General Procedure for the Cyclization of Peptides **15–19**

Linear precursors of peptides **15**–**19** (1 equiv), HOBt (5 equiv), and DIPEA (10 equiv) were dissolved in DMF/NMP 8:2 v/v (100 μL/μmol of peptide) in an Eppendorf tube. Under stirring, a solution of PyBOP (5 equiv) in DMF/NMP 8:2 v/v (100 μL/μmol of peptide) was added, and the mixture was stirred at 20 °C overnight. The crude product was purified by preparative HPLC [column: Phenomenex Luna 10 μm C_18(2)_ 100 Å, 250 × 21 mm; flow: 22 mL/min, solvent A: H_2_O (0.05% TFA), solvent B: MeCN; and gradient A/B: 0–20 min: 97/3, 20–25 min: 2/98].

### [AzbC(*o*,*o*)^1^,Dap^6^]-VP (**15**)

Cyclization yield: 23%. HPLC (gradient: 0–15 min: MeCN/H_2_O + 0.05% TFA 10/90–98/2, 15–20 min: 98/2): *t*_R_: *Z*-isomer: 8.5 min, *E*-isomer: 8.3 min. HR-MS (ESI) calcd *m*/*z* for C_57_H_69_N_17_O_13_ (M + 2H)^2+^, 600.7703; observed, 600.7711.

### [AzbC(*m*,*o*)^1^,Dap^6^]-VP (**16**)

Cyclization yield: 45%. HPLC (gradient: 0–15 min: MeCN/H_2_O + 0.05% TFA 10/90–98/2, 15–20 min: 98/2): *t*_R_: *Z*-isomer: 8.3 min, *E*-isomer: 8.7 min. HR-MS (ESI) calcd *m*/*z* for C_57_H_69_N_17_O_13_ (M + 2H)^2+^*m*/*z*, 600.7703; observed, 600.7717.

### [AzbC(*m*,*m*)^1^,Dap^6^]-VP (**17**)

Cyclization yield: 48%. HPLC (gradient: 0–15 min: MeCN/H_2_O + 0.05% TFA 10/90–98/2, 15–20 min: 98/2): *t*_R_: *Z*-isomer: 8.1 min, *E*-isomer: 8.6 min. HR-MS (ESI) calcd *m*/*z* for C_57_H_69_N_17_O_13_ (M + 2H)^2+^, 600.7703; observed, 600.7713.

### [AzpC(*o*)^1^,Dap^6^]-VP (**18**)

Cyclization yield: 60%. HPLC (gradient: 0–15 min: MeCN/H_2_O + 0.05% TFA 10/90–98/2, 15–20 min: 98/2): *t*_R_: *Z*-isomer: 7.7 min, *E*-isomer: 8.2 min. HR-MS (ESI) calcd *m*/*z* for C_57_H_73_N_19_O_13_ (M + 2H)^2+^, 616.7890; observed, 616.7902.

### [AzpC(*m*)^1^,Dap^6^]-VP (**19**)

Cyclization yield: 57%. HPLC (gradient: 0–15 min: MeCN/H_2_O + 0.05% TFA 10/90–98/2, 15–20 min: 98/2): *t*_R_: *Z*-isomer: 7.6 min, *E*-isomer: 8.2 min. HR-MS (ESI) calcd *m*/*z* for C_57_H_73_N_19_O_13_ (M + 2H)^2+^, 616.7890; observed, 616.7902.

### Pharmacology: Cell Culture

The stable HEK293 cell lines overexpressing either human (h) OTR, V_1a_R, or V_1b_R were stored at −80 °C in 700 μL of freezing medium (FBS with 10% DMSO). After thawing, the cells were transferred into a cell culture flask containing 10 mL of Dulbecco’s modified eagle medium (DMEM) with 10% FBS, 2 mM glutamine, and 1 mM sodium pyruvate and incubated at 37 °C for 24 h. The old medium was aspirated to remove the DMSO, and 10 mL of fresh medium was transferred into the flask. In addition to the growth medium, 160 μL of a 50 mg/mL G-418 solution was added. Because the stable cell lines carry a neomycin-Geneticin resistance gene alongside the gene for the receptor of interest, the G-418 antibiotic was included with the medium when working with the stable cell lines. The cells were incubated at 37 °C and 5% CO_2_ and grown to confluences of 70–100%. Cells were split after 3 to 4 days. The medium was removed, and cells were detached using 3 mL of trypsin–EDTA solution followed by 9 mL of fresh medium. The cells were pipetted up and down to detach all of them. After centrifugation (178*g*, 1000 rpm, 3 min), the cells were resuspended in 1 mL of medium, counted with the Neubauer counting chamber, and plated in 384-well plates.

### Preparation of Ligands

A 2-fold concentrated dilution series in stimulation buffer was prepared for each isomer. To get to the respective *Z*-isomer of each compound, the highest concentrated dilution was irradiated with 340 nm for 1 min in an Eppendorf tube. After that, the dilution series was prepared, and the Eppendorf tubes were covered with aluminum foil. The solutions of the *E* isomers were not irradiated prior to the dilution.

### IP-One Assay (CisBio HTRF IP-One Assay): Agonist Function

Measurement of the OTR, V_1a_R, and V_1b_R activation was done with the IP-One HTRF assay from CisBio. The experiments were done according to the manufacturer’s protocol. Cells were seeded into a white 384-well plate with F-bottom (10,000 cells/well) and incubated for 48 h at 37 °C and 5% CO_2_. The medium was removed, and 5 μL of stimulation buffer was added to each well, followed by 15 min incubation at 37 °C and 5% CO_2_. Subsequently, 5 μL of compound dilution prepared with stimulation buffer was added. The plate was incubated for 1 h at 37 °C and 5% CO_2_. After the incubation, 5 μL of IP1-d2 conjugate and 5 μL of anti-IP1-cryptate-TB conjugate dissolved in lysis buffer were added to each well and incubated for another 60 min at 20 °C. Fluorescence emission measurements at 615 and 665 nm were performed using a Spark Multimode plate reader (Tecan, Männedorf, Switzerland) at an excitation wavelength of 340 nm. Results were analyzed as a ratio of fluorescence intensities of 665 to 615 nm and normalized to OT and VP.

### Antagonist Function

To assess the antagonistic properties of compounds **13**–**19**, a 4-fold concentrated dilution series in stimulation buffer was prepared for each isomer. Separately, a 4-fold concentrated VP solution was prepared (4 nM, final concentration 1 nM). A 1:1 mixture of VP solution and the diluted ligand was made to end up with a 2-fold concentration of both compounds. The following steps were conducted as described above for the agonist mode. The FRET ratios (665/615 nm) were calculated and obtained values were normalized to the basal activity value (0%) and maximal effect of VP (100%). Atosiban was used as a positive control.

### In Vitro Immunostaining and Confocal Scanning Microscopy: Cell Culture and Stimulation

HEK293 cells stably expressing a C-terminal-conjugated GFP human V_1b_R were cultured in a full growth medium consisting of DMEM/GlutaMAX (Gibco, Thermo Fisher), containing 0.5 mg/mL G-418 (Gibco, Thermo Fisher), 1 mM sodium pyruvate (Gibco, Thermo Fisher), and 10% fetal bovine serum (Gibco, Thermo Fisher). For stimulation experiments, cells were plated on poly-d-lysine (Sigma-Aldrich, Merck)-coated glass coverslips at a density of 50,000 cells per well overnight in a full growth medium. The following day, the full growth medium was replaced with a serum-free medium to synchronize cells. The next day, HEK293 cells were stimulated with VP (10 nM) as a positive control, serum-free medium without G-418 as a negative control, and 1 nM nonactivated *E***-12** or diode (340 nm) activated *Z***-12** for 60 min. Solutions were prepared in a serum-free medium without G-418. Finally, coverslips were washed with 1× PBS (Sigma) and fixed with 4% paraformaldehyde solution in PBS for 30 min on ice.

### Immunostainings and Imaging

Coverslips were first incubated with 5% normal donkey serum (Jackson Immuno Research), 2% bovine serum albumin (Sigma-Aldrich, Merck), and 0.2% Triton X-100 (Sigma-Aldrich, Merck) to block nonspecific protein binding. Next, a primary antibody cocktail containing FITC-conjugated goat-anti-GFP (1:2000, Millipore) and mouse pCREB (1:500, Millipore) was added overnight incubation at 4 °C. After three PBS washing steps, wells were incubated with secondary antibody cocktails (1:500, Jackson Immuno Research) containing suitable conjugated fluorophores, as well as Hoechst 33,342 (1:5000, Sigma-Aldrich, Merck) as nuclear counterstain. After careful, extensive washing, the coverslips were mounted with glycerol gelatin (Sigma-Aldrich, Merck) and imaged on a Zeiss LSM880 confocal microscope. Images were analyzed with ZEN pro (Zeiss) ImageJ 1.52a and collated in CorelDraw 2019. To determine pCREB activation, the fluorescence was normalized to the negative control (cells treated with a serum-free medium without G-418 instead of **12**), as already minor receptor interactions can lead to pCREB activity in overexpression systems.

## Supporting Information Available

Purity of the compounds, photophysical characterization, additional pharmacological figures, and NMR spectra (PDF)
Molecular formula strings (CSV)

## Abbreviations

AA: amino acid
Arg: arginine
Asn: asparagine
cAMP: cyclic adenosine monophosphate
CD: circular dichroism
CREB: cAMP response element binding protein
Cys: cysteine
Dap: diaminopropionic acid
DMEM: Dulbecco’s modified Eagle medium
DIC: *N*,*N*′-diisopropylcarbodiimide
DIPEA: *N*,*N*-diisopropylethylamine
DMF: dimethylformamide
DMSO: dimethyl sulfoxide
EDTA: ethylenediaminetetraacetic acid
FBS: fetal bovine serum
Fmoc: fluorenylmethoxycarbonyl
FRET: fluorescence resonance energy transfer
GFP: green fluorescent protein
Gln: glutamine
Gly: glycine
GPCR: G protein-coupled receptor
HEPES: (4-(2-hydroxyethyl)-1-piperazineethanesulfonic acid)
HBTU: 2-(1*H*-benzotriazol-1-yl)-1,1,3,3-tetramethyluronium hexafluorophosphate
HEK: human embryonic kidney
hOTR: human oxytocin receptor
hV_1a_R: human vasopressin receptor 1a
hV_1b_R: human vasopressin receptor 1b
hVPRs: human vasopressin receptors (all)
HOBt: hydroxybenzotriazole
HPLC: high-performance liquid chromatography
Ile: isoleucine
IP: inositol monophosphate
Leu: leucine
*m*: *meta*
MeCN: acetonitrile
NMP: *N*-methyl-2-pyrrolidone
*o*: *ortho*
OT: oxytocin
OTR: oxytocin receptor
p.a.: per analysis
Pbf: 2,2,4,6,7-pentamethyldihydrobenzofuran-5-sulfonyl
PBS: phosphate buffered saline
pCREB: phosphorylated CREB
Phe: phenylalanine
Pro: proline
PS: polystyrene
PSS: photostationary state
PyBOP: benzotriazol-1-yloxytripyrrolidinophosphonium hexafluorophosphate
RP: reversed phase
SEM: standard error of the mean
SPPS: solid-phase peptide synthesis
*t*_1/2_: thermal half-life
^*t*^Bu: *tert*-butyl
TFA: trifluoroacetic acid
TFE: trifluoroethanol
TIPS: triisopropylsilane
Trt: trityl
Tyr: tyrosine
VP: vasopressin

## Nomenclature

Azb: azobenzene
Azp: arylazopyrazole
Dap: diaminopropionic acid
S: side chain (photoswitch **1**)
L: linear (photoswitch **2**)
C: cyclic (photoswitches **3**–**7**).
