# Supplementary material for: Photoswitchable Probes of Oxytocin and Vasopressin
Source: J Med Chem. 2023 Oct 19;66(21):14853–65. doi: 10.1021/acs.jmedchem.3c01415 (PMC10641831; doi:10.1021/acs.jmedchem.3c01415)
Supplement: Supplementary file 1 — jm3c01415_si_001.pdf [file jm3c01415_si_001.pdf]

SUPPLEMENTARY INFORMATION

Photoswitchable Probes of Oxytocin and Vasopressin

Ulrike Wirth,[1] Konstantin Raabe,[2] Predrag Kalaba,[2] Erik Keimpema,[3] Markus Muttenthaler,*[2,4] Burkhard König*[1]

[1] Institute of Organic Chemistry, Department of Chemistry and Pharmacy, University of Regensburg, Universitätsstraße 31, 93053 Regensburg, Germany

[2] Institute of Biological Chemistry, Department of Chemistry, University of Vienna, Währinger Straße 38, 1090 Vienna, Austria

[3] Center for Brain Research, Department of Molecular Neurosciences, Medical University of Vienna, Spitalgasse 4, 1090, Vienna, Austria

[4] Institute for Molecular Bioscience, The University of Queensland, St. Lucia, 4072, Brisbane, Australia

Table of Contents

1. Chemistry 3
   1.1. Synthetic Procedures 3
   1.2. Purity of Photoprobes 4
   1.3. Photophysical Properties 16
      1.3.1. UV/Vis Spectra and Cycle Performance 16
      1.3.2. Photostationary States 21
      1.3.3. Circular dichroism (CD) Spectroscopy 28
      1.3.4. Thermal Half-lives 29
2. Pharmacology 31
   2.1. Supplementary Figures 31
3. NMR Spectra 34
4. References 39

1. Chemistry

1.1. Synthetic Procedures

Compounds **1**[1] and **2**[2] were synthesized according to literature-known procedures.

<u>General procedure for the synthesis of the nitroso-compounds **24, 25, 28** and **29**[3]</u>

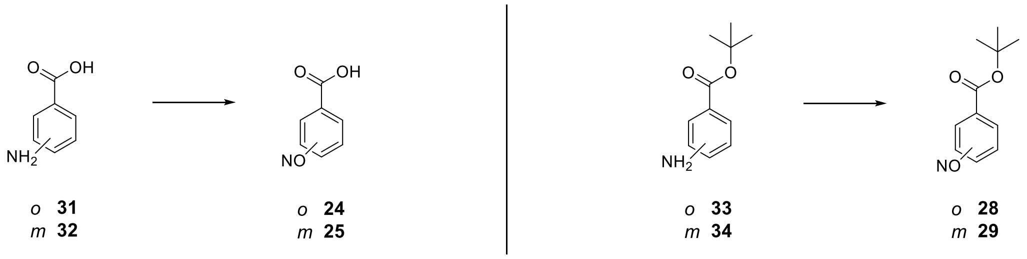

Amines **31**-**34** (1.0 eq.) were dissolved in CH_2Cl_2. Oxone (2.0 eq.) dissolved in water was added to this solution. The solution was vigorously stirred under nitrogen at 20 °C until TLC monitoring indicated complete consumption of the starting material. The carboxylic acids **24** and **25** formed a precipitate which was filtered off, washed with water, and dried. For esters **28** and **29,** the organic phase was washed with 1 M HCl, saturated $NaHCO_3$, and brine. The solvent was removed *in vacuo*. The products were used without further purification for the next step.

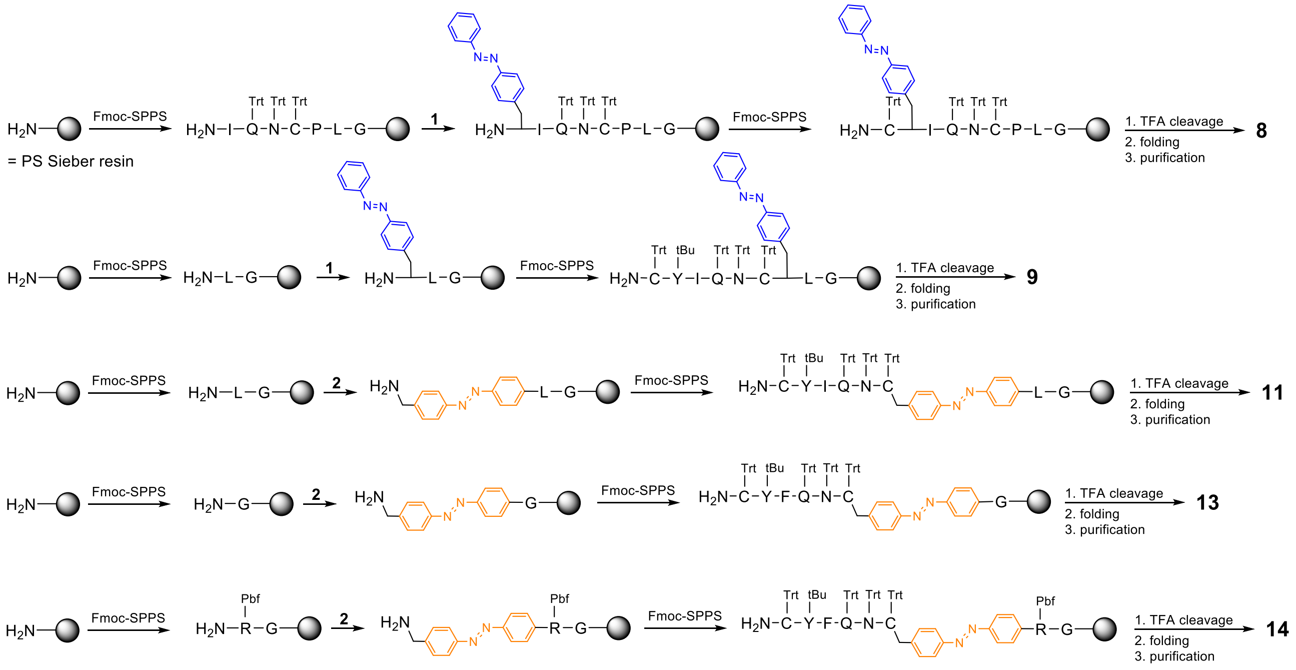

Scheme S1. Synthetic schemes describing the synthesis of peptides **8**, **9**, **11**, **13** and **14**. Protecting groups: Trt = trityl, ^{t}Bu = *tert*-butyl, Pbf = 2,2,4,6,7-pentamethyldihydrobenzofuran-5-sulfonyl.

1.2. Purity of Photoprobes

Purity was determined by analytical RP-HPLC at 220 nm in DMSO.

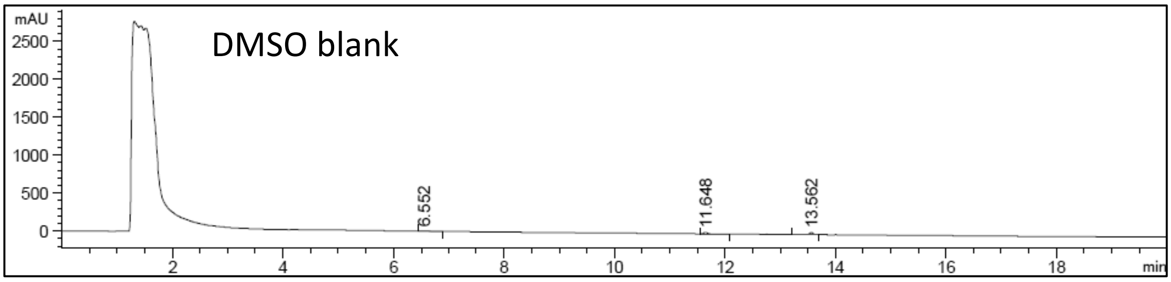

Purity: **99%** (*Z*-Isomer: 27% + *E*-isomer: 73%)

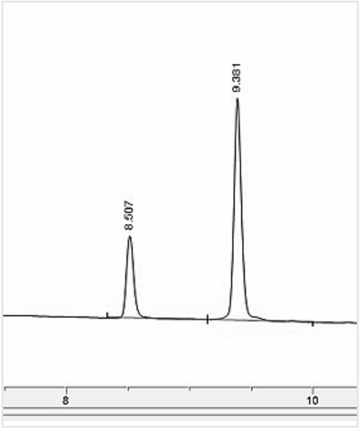

Signal 2: DAD1 B, Sig=220,4 Ref=off

Peak #	RetTime [min]	Type	Width [min]	Area [mAU*s]	Height [mAU]	Area %
1	8.507	BV R	0.0645	937.61279	217.43913	26.5484
2	9.381	VV R	0.0672	2580.50317	586.88135	73.0668
3	13.137	BB	0.1036	13.58862	2.04224	0.3848

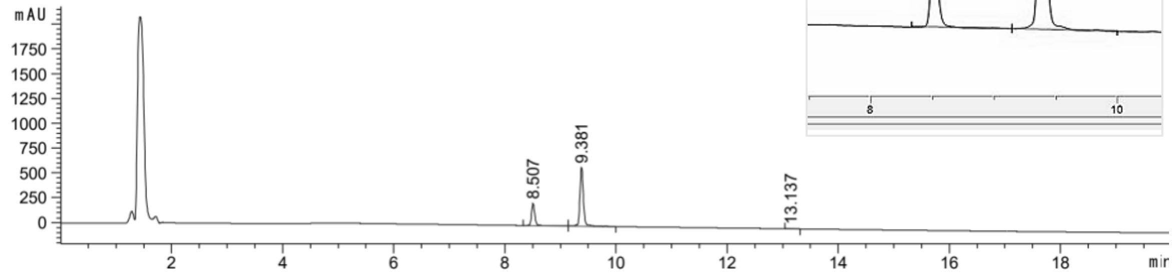

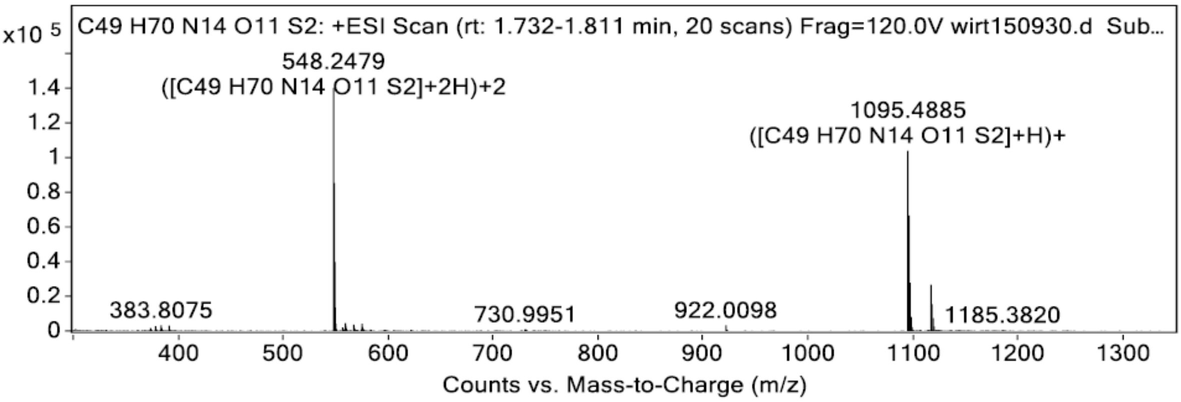

Figure S1. Analytical C_{18}-RP-HPLC trace and high-resolution mass spectrum of **8**.

Purity: **96%** (*Z*-Isomer: 22% + *E*-isomer: 74%)

Signal 2: DAD1 B, Sig=220,4 Ref=off

Peak #	RetTime [min]	Type	Width [min]	Area [mAU*s]	Height [mAU]	Area %
1	7.500	MM	0.0664	51.19547	12.85047	3.8045
2	8.880	BB	0.0607	302.78906	75.67075	22.5012
3	9.774	VB R	0.0633	991.67480	244.42427	73.6943

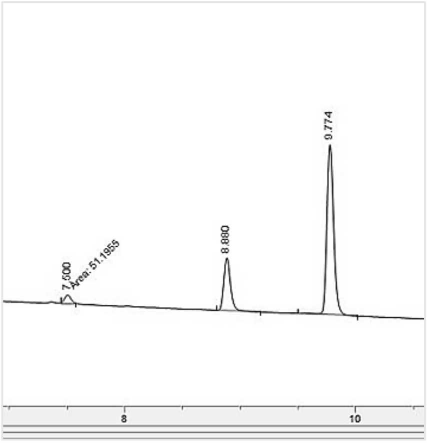

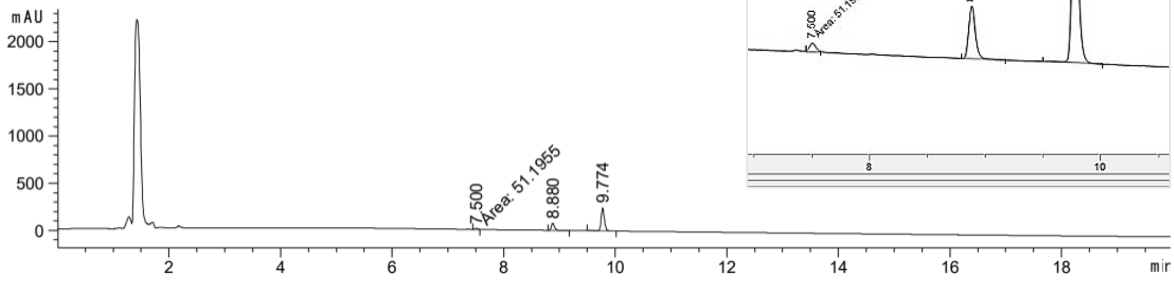

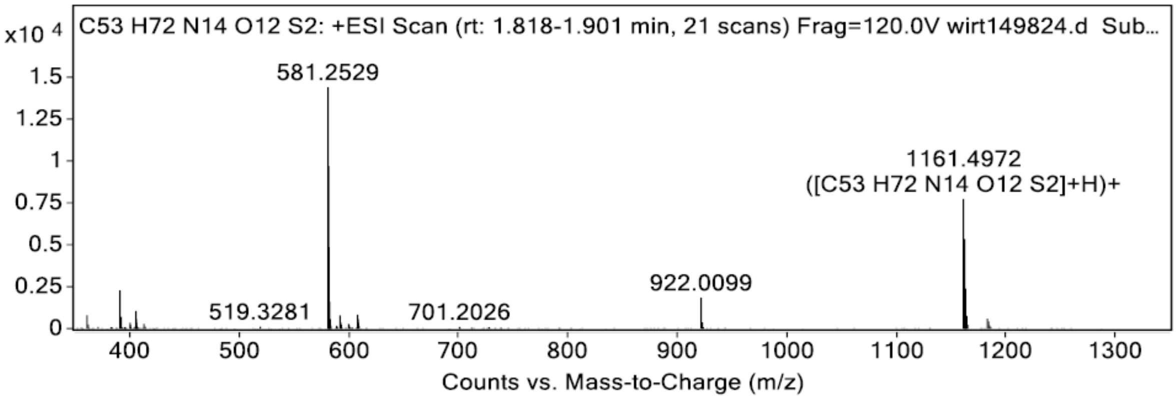

Figure S2. Analytical C_{18}-RP-HPLC trace and high-resolution mass spectrum of **9**.

Purity: **99%** (*Z*-Isomer: 26% + *E*-isomer: 74%)

Signal 2: DAD1 B, Sig=220,4 Ref=off

Peak #	RetTime [min]	Type	Width [min]	Area [mAU*s]	Height [mAU]	Area %
1	5.894	BB	0.0792	6.42033	1.26486	0.4999
2	7.582	VV R	0.0666	333.65219	76.87822	25.9803
3	7.888	VB	0.0623	944.17615	237.92987	73.5197

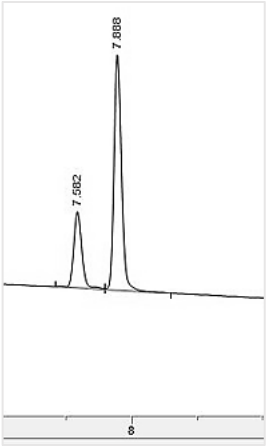

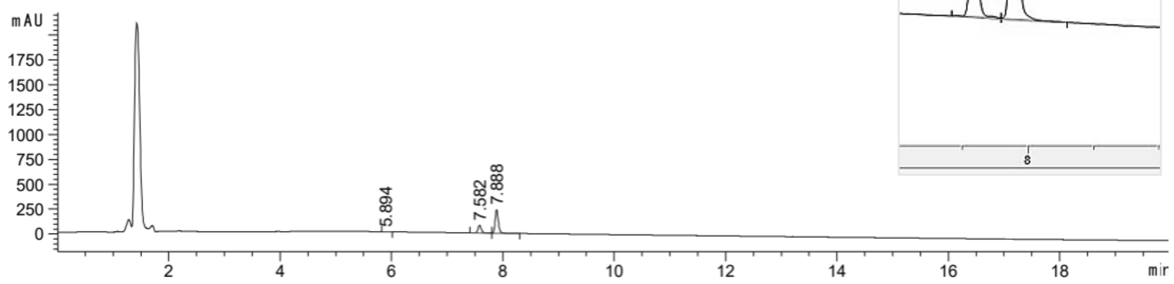

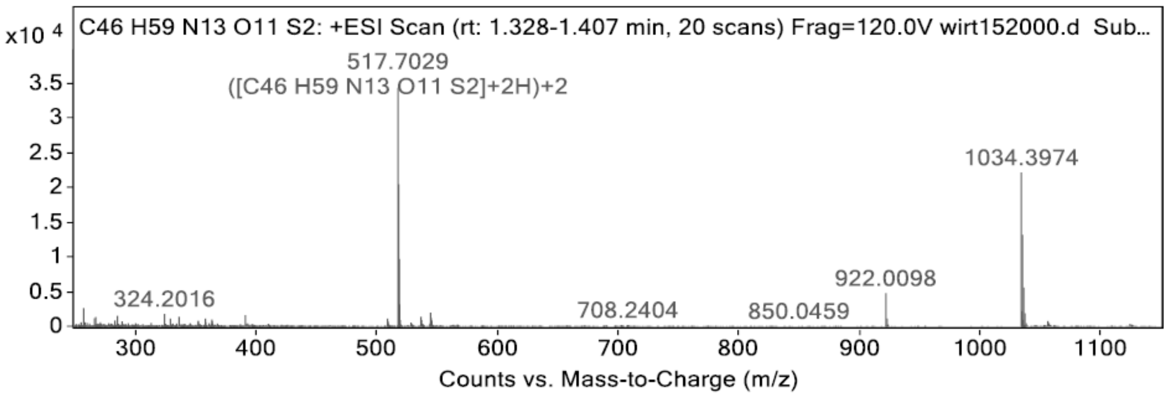

Figure S3. Analytical C_{18}-RP-HPLC trace and high-resolution mass spectrum of **10**.

Purity: **99%** (*Z*-Isomer: 16% + *E*-isomer: 83%)

Signal 2: DAD1 B, Sig=220,4 Ref=off

Peak #	RetTime [min]	Type	Width [min]	Area [mAU*s]	Height [mAU]	Area %
1	8.520	BV	0.0618	207.13811	50.56302	15.6977
2	8.872	VB	0.0621	1099.98376	266.77216	83.3608
3	13.152	BB	0.0829	12.42412	2.37917	0.9415

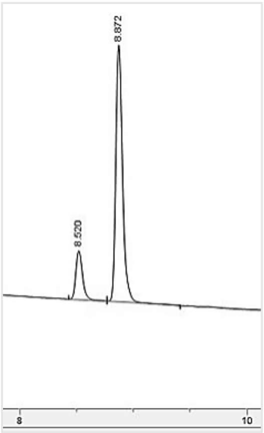

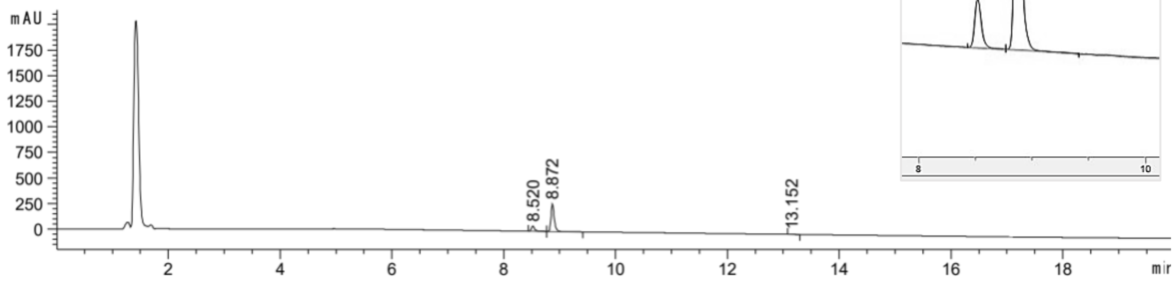

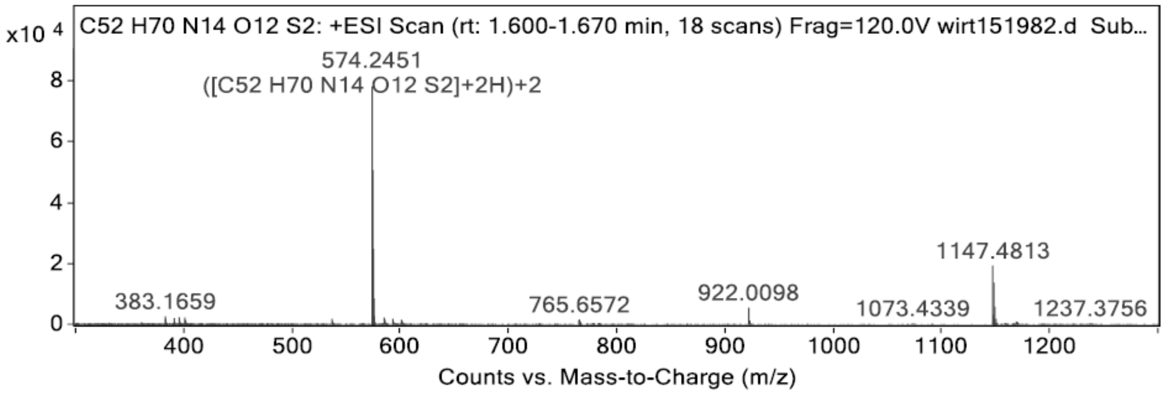

Figure S4. Analytical C_{18}-RP-HPLC trace and high-resolution mass spectrum of **11**.

Purity: **99%** (*Z*-Isomer: 26% + *E*-isomer: 74%)

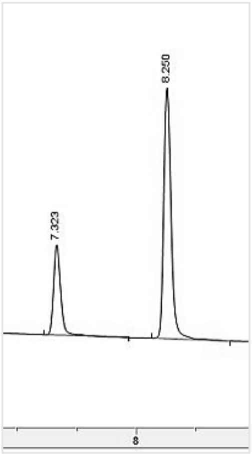

Signal 2: DAD1 B, Sig=220,4 Ref=off

Peak #	RetTime [min]	Type	Width [min]	Area [mAU*s]	Height [mAU]	Area %
1	7.323	BB	0.0645	664.36975	159.92587	25.8232
2	8.250	BB	0.0659	1898.35449	444.14838	73.7867
3	13.180	BB	0.0810	10.03463	1.92021	0.3900

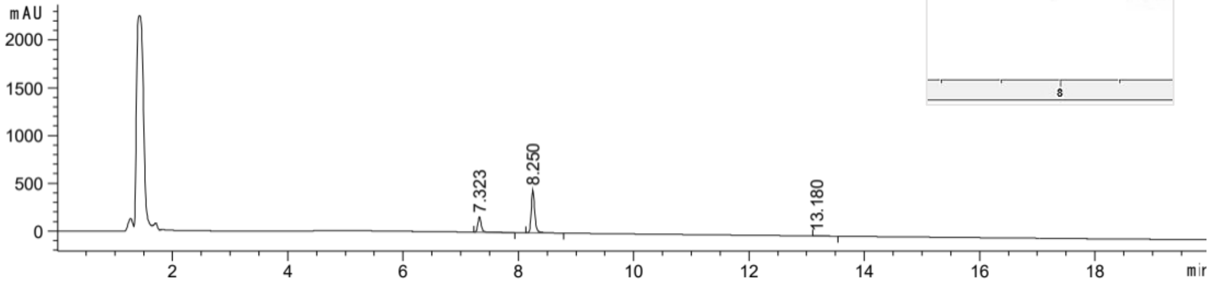

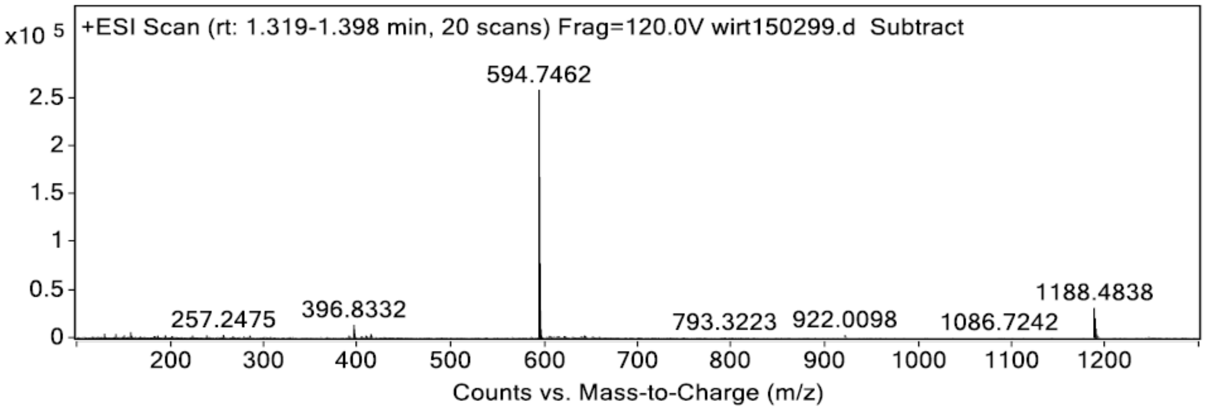

Figure S5. Analytical C_{18}-RP-HPLC trace and high-resolution mass spectrum of **12**.

Purity: **98%** (*Z*-Isomer: 18% + *E*-isomer: 81%)

Signal 2: DAD1 B, Sig=220,4 Ref=off

Peak #	RetTime [min]	Type	Width [min]	Area [mAU*s]	Height [mAU]	Area %
1	6.827	VB	0.0654	19.16032	4.35307	0.9534
2	7.855	BV	0.0616	360.17862	92.14953	17.9224
3	8.027	VB	0.0599	1622.89905	412.86786	80.7549
4	13.198	BB	0.0954	7.42210	1.11909	0.3693

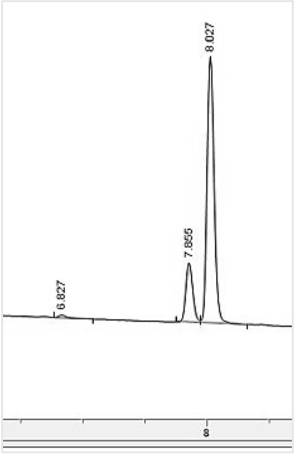

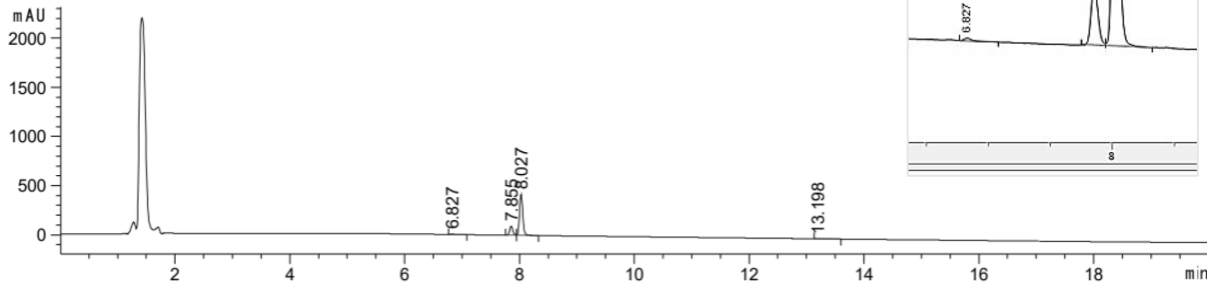

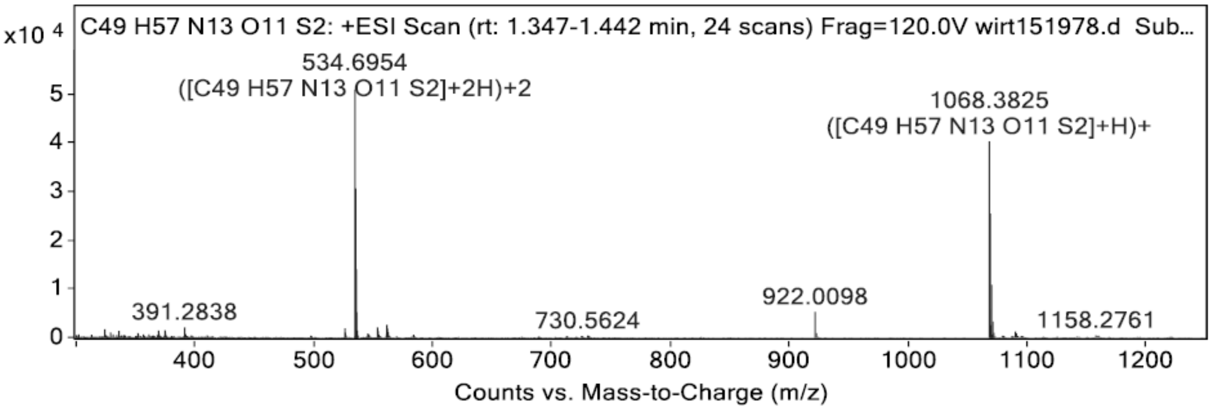

Figure S6. Analytical C_{18}-RP-HPLC trace and high-resolution mass spectrum of **13**.

Purity: **99%** (*Z*-Isomer: 13% + *E*-isomer: 87%)

Signal 2: DAD1 B, Sig=220,4 Ref=off

Peak #	RetTime [min]	Type	Width [min]	Area [mAU*s]	Height [mAU]	Area %
1	7.368	BV E	0.0602	298.77222	75.39442	13.1674
2	7.504	VB R	0.0645	1963.04138	472.67993	86.5145
3	13.198	BB	0.0905	7.21912	1.16171	0.3182

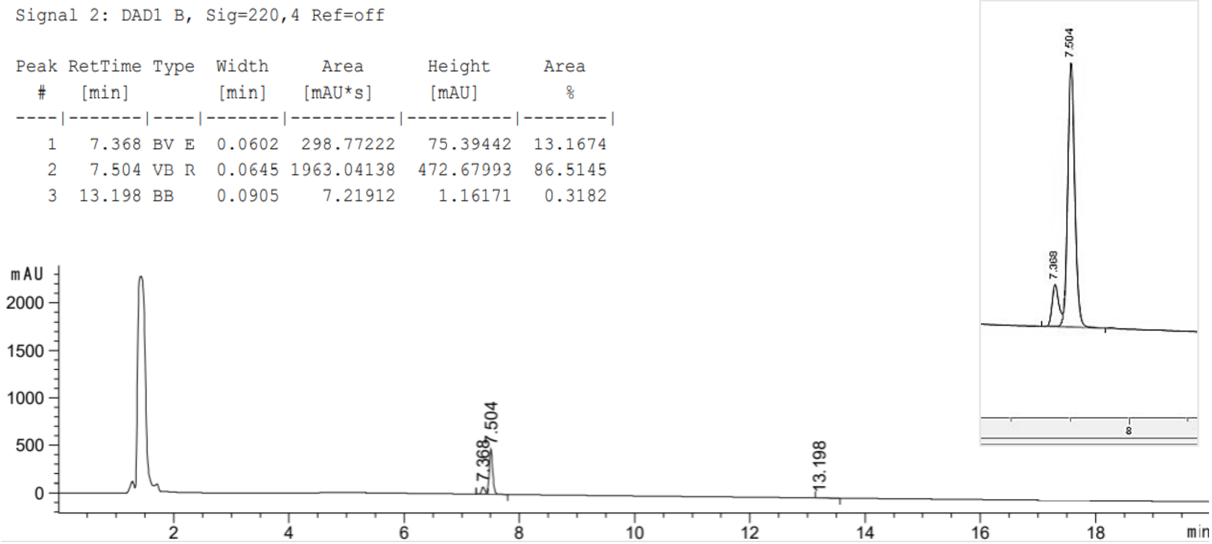

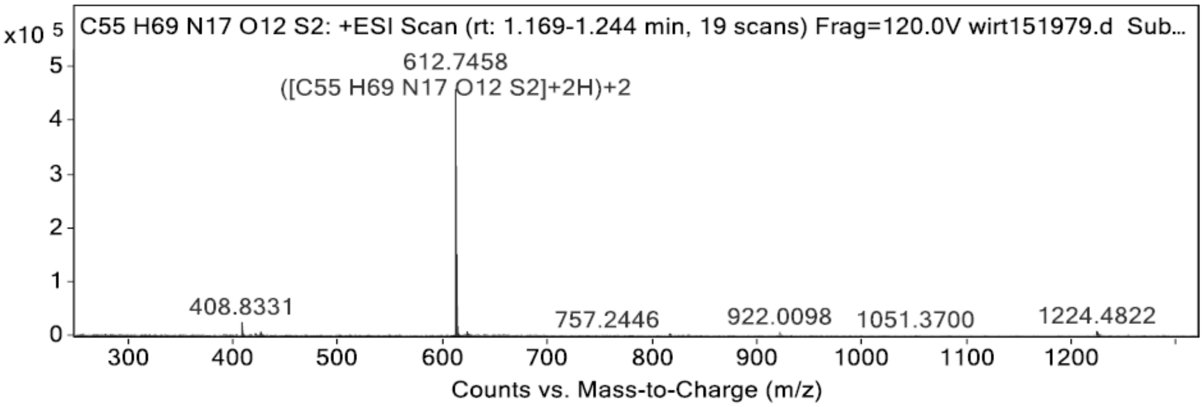

Figure S7. Analytical C_{18}-RP-HPLC trace and high-resolution mass spectrum of **14**.

Purity: **99%** (*Z*-Isomer: 29% + *E*-isomer: 70%)

Signal 2: DAD1 B, Sig=220,4 Ref=off

Peak #	RetTime [min]	Type	Width [min]	Area [mAU*s]	Height [mAU]	Area %
1	3.957	BB	0.0885	27.36912	4.80377	1.2536
2	8.267	BV	0.0645	1525.45618	367.16348	69.8716
3	8.470	VB	0.0610	630.40137	156.56445	28.8748

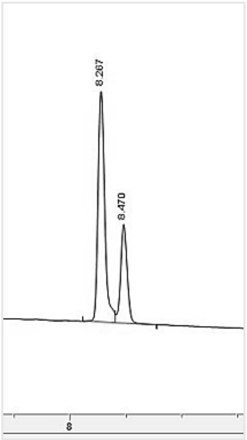

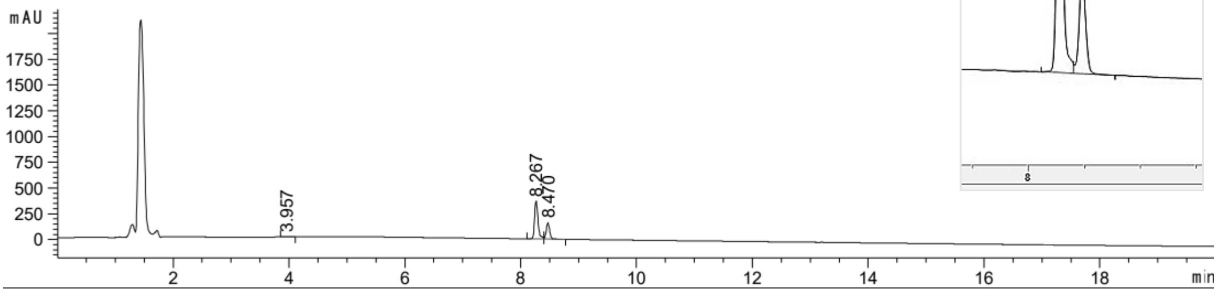

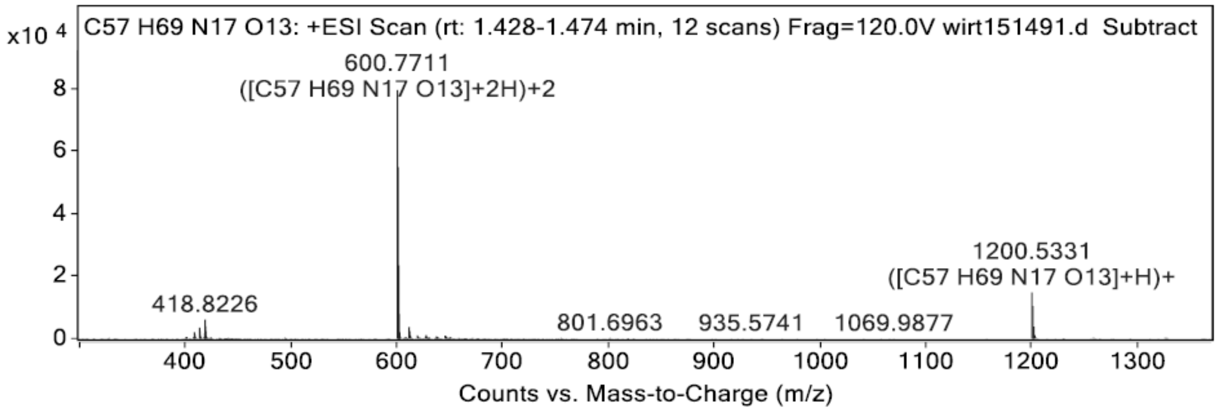

Figure S8. Analytical C_{18}-RP-HPLC trace and high-resolution mass spectrum of **15**.

Purity: **99%** (*Z*-Isomer: 18% + *E*-isomer: 81%)

Signal 2: DAD1 B, Sig=220,4 Ref=off

Peak #	RetTime [min]	Type	Width [min]	Area [mAU*s]	Height [mAU]	Area %
1	8.082	BV	0.0732	21.57994	4.55996	0.6797
2	8.297	VV	0.0876	566.69836	97.78909	17.8485
3	8.655	VB	0.1871	2586.77246	194.57198	81.4718

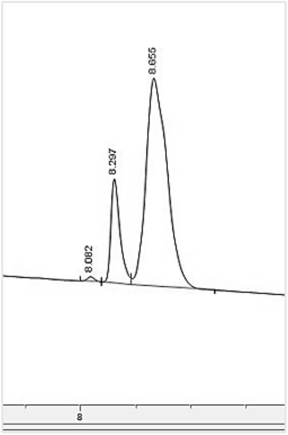

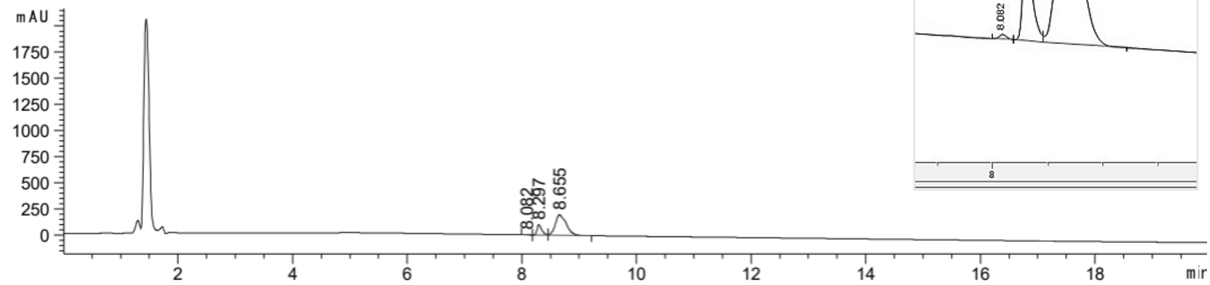

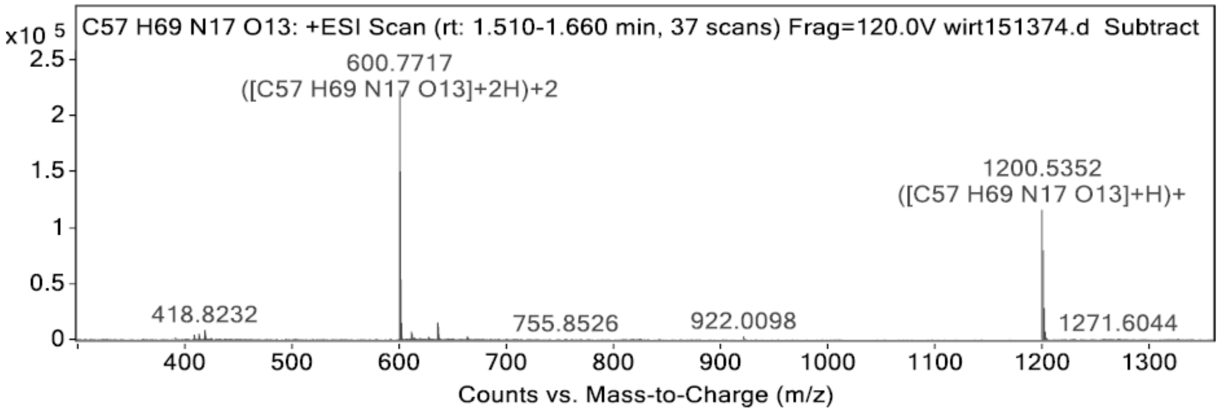

Figure S9. Analytical C_{18}-RP-HPLC trace and high-resolution mass spectrum of **16**.

Purity: **99%** (*Z*-Isomer: 13% + *E*-isomer: 87%)

Signal 2: DAD1 B, Sig=220,4 Ref=off

Peak #	RetTime [min]	Type	Width [min]	Area [mAU*s]	Height [mAU]	Area %
1	8.050	BV	0.0916	297.20914	47.13602	13.4929
2	8.641	VB	0.0657	1905.49341	447.25916	86.5071

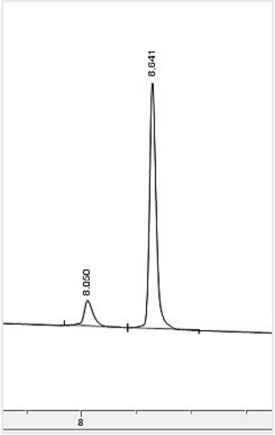

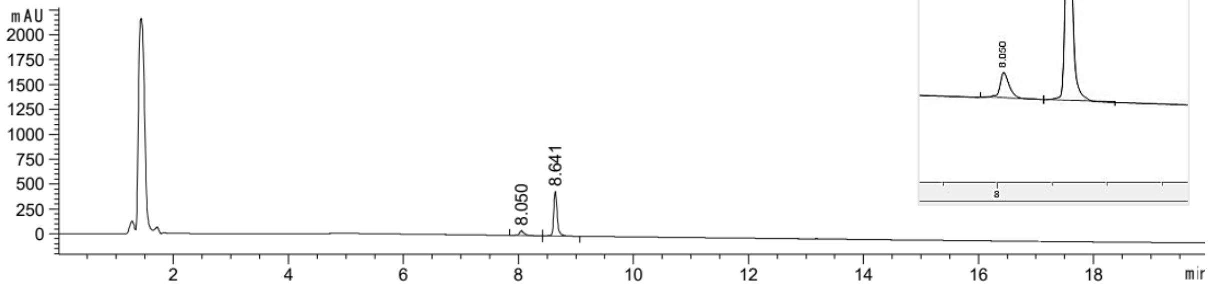

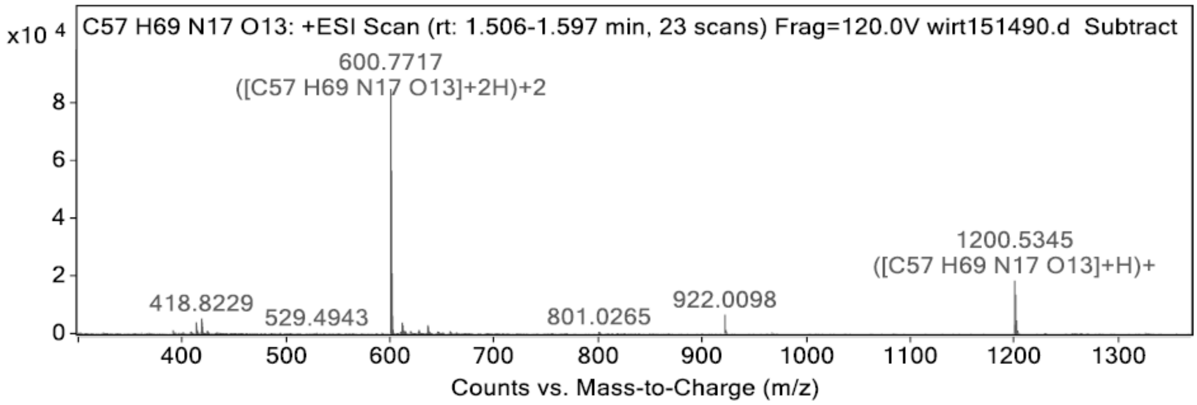

Figure S10. Analytical C_{18}-RP-HPLC trace and high-resolution mass spectrum of **17**.

Purity: **99%** (*Z*-Isomer: 17% + *E*-isomer: 83%)

Signal 2: DAD1 B, Sig=220,4 Ref=off

Peak #	RetTime [min]	Type	Width [min]	Area [mAU*s]	Height [mAU]	Area %
1	7.703	BB	0.0724	685.64569	146.96362	17.4853
2	8.207	BB	0.1037	3235.62939	450.92896	82.5147

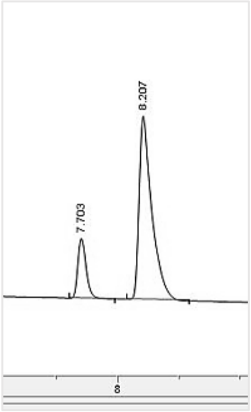

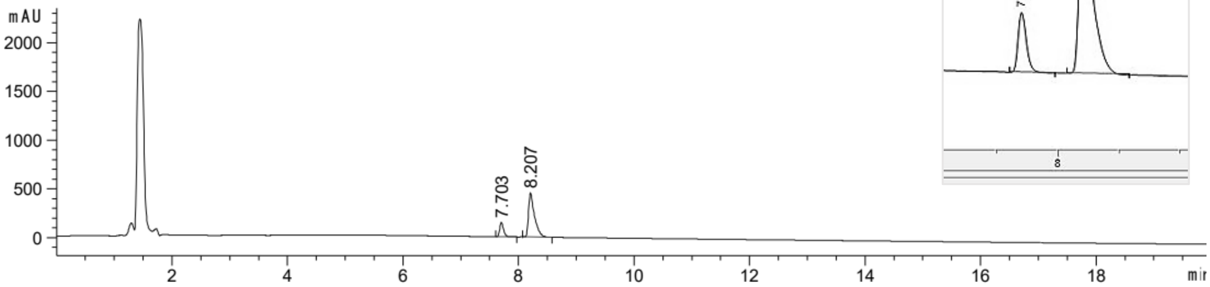

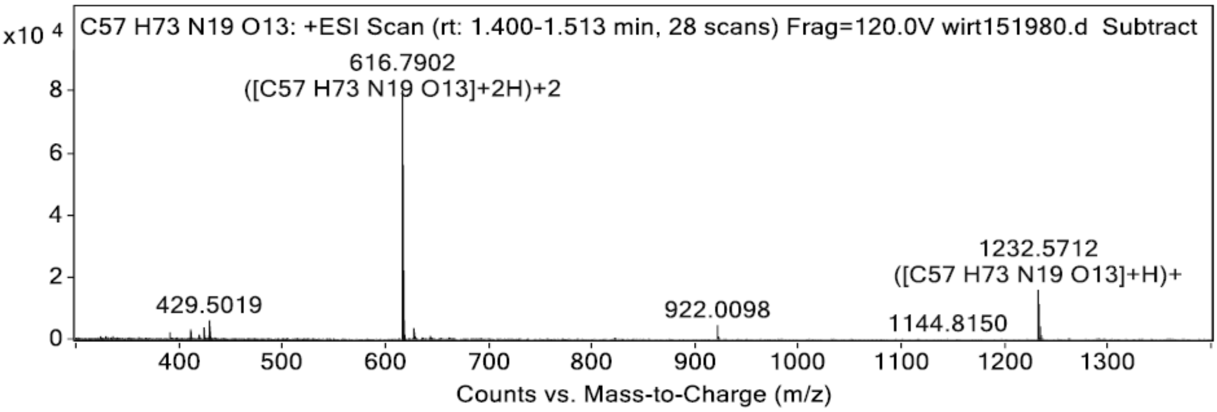

Figure S11. Analytical C_{18}-RP-HPLC trace and high-resolution mass spectrum of **18**.

Purity: **99%** (*Z*-Isomer: 25% + *E*-isomer: 75%)

Signal 2: DAD1 B, Sig=220,4 Ref=off

Peak #	RetTime [min]	Type	Width [min]	Area [mAU*s]	Height [mAU]	Area %
1	7.632	BB	0.0874	873.05621	146.69434	24.6998
2	8.231	BB	0.0637	2661.60620	650.82031	75.3002

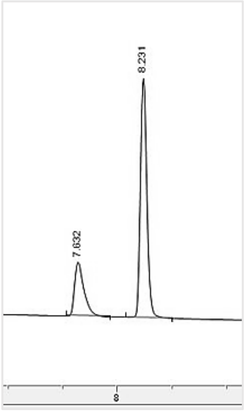

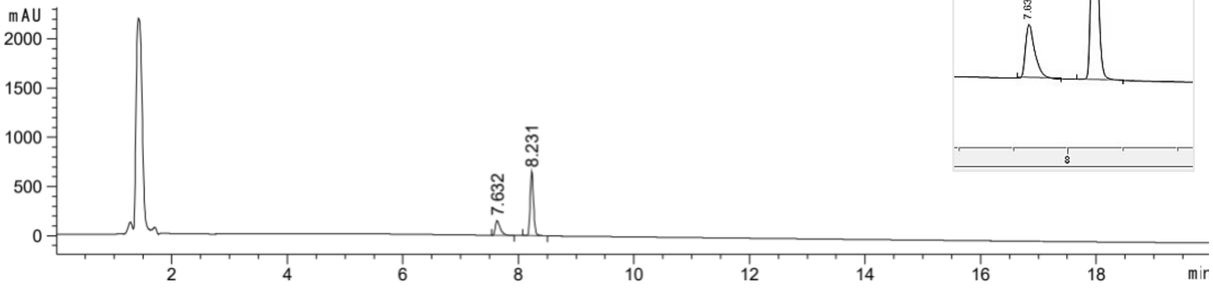

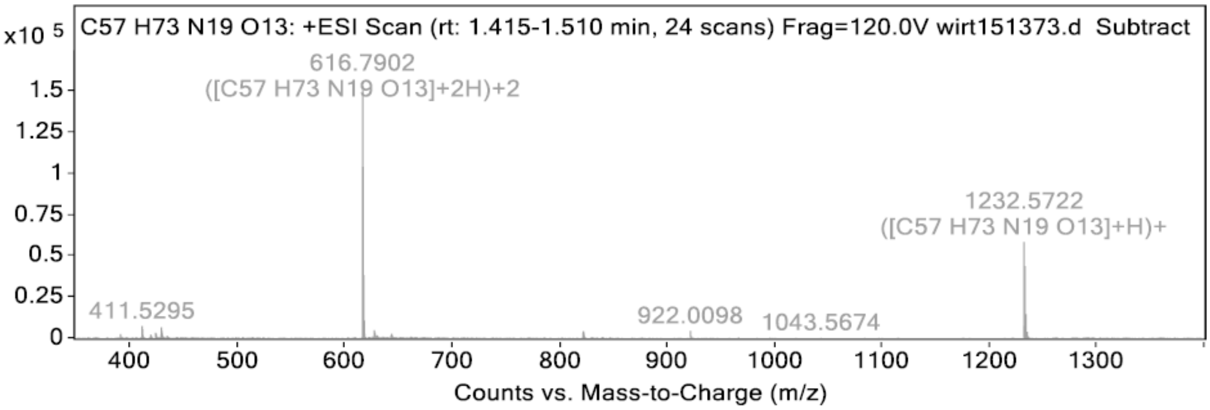

Figure S1. Analytical C_{18}-RP-HPLC trace and high-resolution mass spectrum of **19**.

1.3. Photophysical Properties

1.3.1. UV/Vis Spectra and Cycle Performance

UV/vis spectra were measured in HEPES buffer (20 µM + 0.1% DMSO, pH 7.5). The spectra were measured in quartz glass cuvettes. First, the solution was illuminated with 340 nm for 10 s to switch to the *Z*-isomer. After measuring the UV/vis spectra of the *Z*-isomers, the solution was illuminated at 420 nm (**8**-**17**) or 528 nm (**18** and **19**) for 60 s, respectively, and the spectra for the *E*-isomer were measured.

To assess the cycle performance, the solutions were irradiated alternating with 340 nm and 420 nm (**8**-**17**) or 528 nm (**18** and **19**). A UV/vis spectrum was measured after each switching step. This was repeated ten times to demonstrate the stability of the compounds. The absorption of the maximum of the *E*-isomer was plotted against the cycle number.

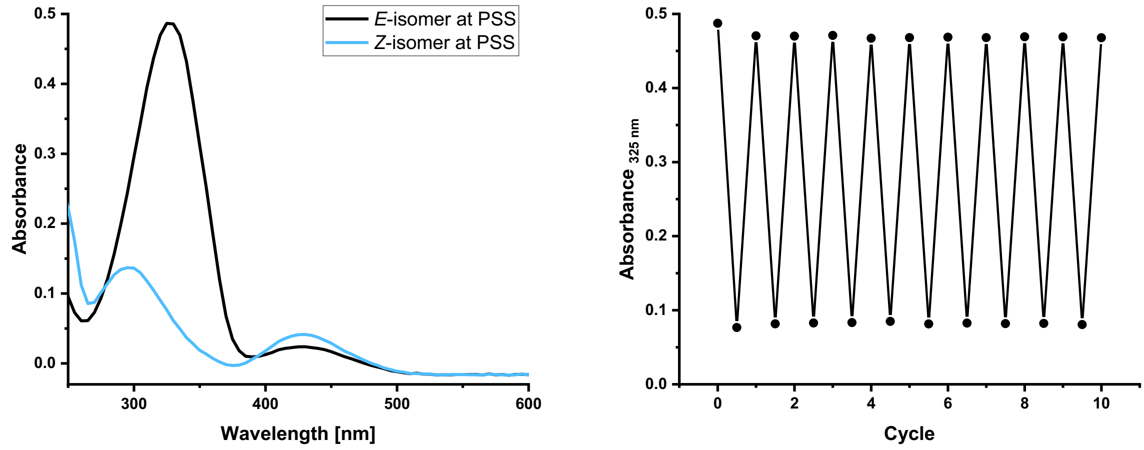

Figure S2. UV/vis spectra and cycle performance of **8**.

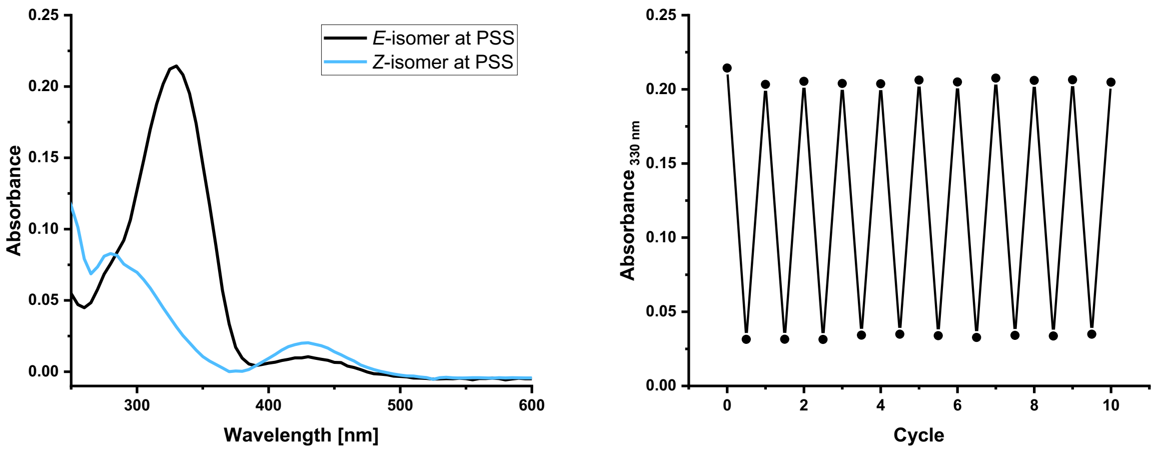

Figure S3. UV/vis spectra and cycle performance of **9**.

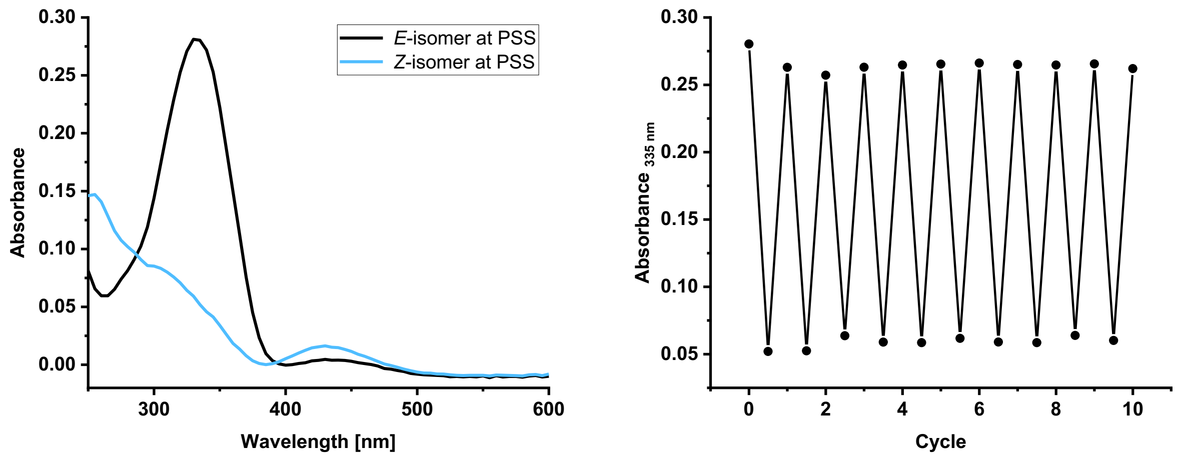

Figure S4. UV/vis spectra and cycle performance of **10**.

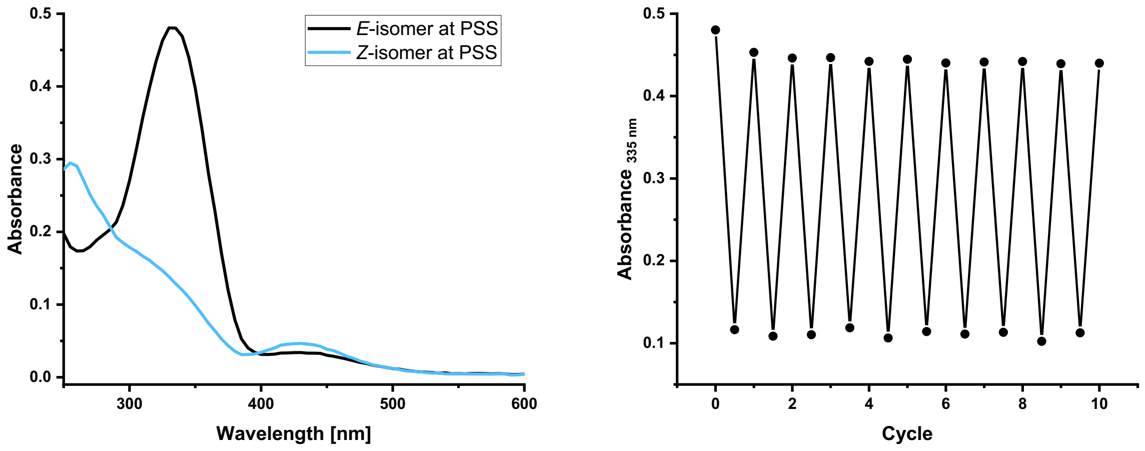

Figure S5. UV/vis spectra and cycle performance of **11**.

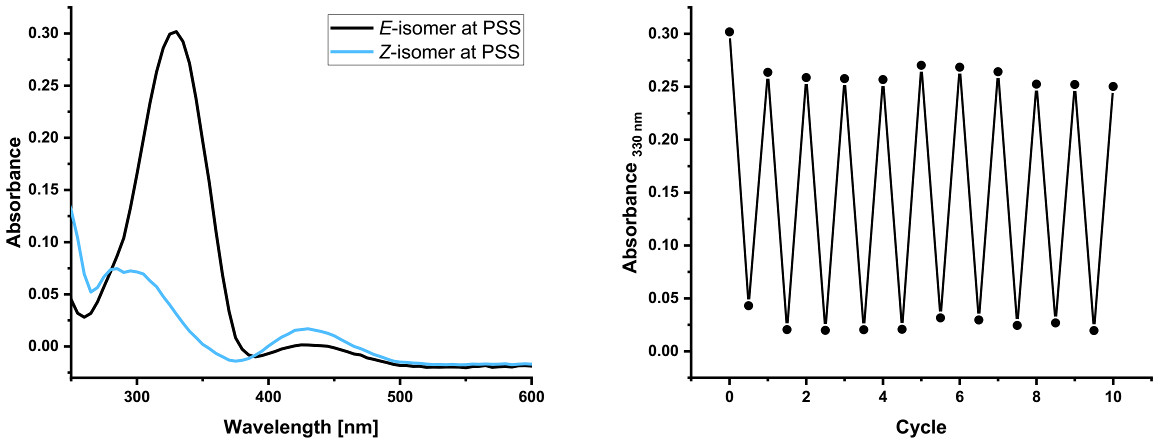

Figure S6. UV/vis spectra and cycle performance of **12**.

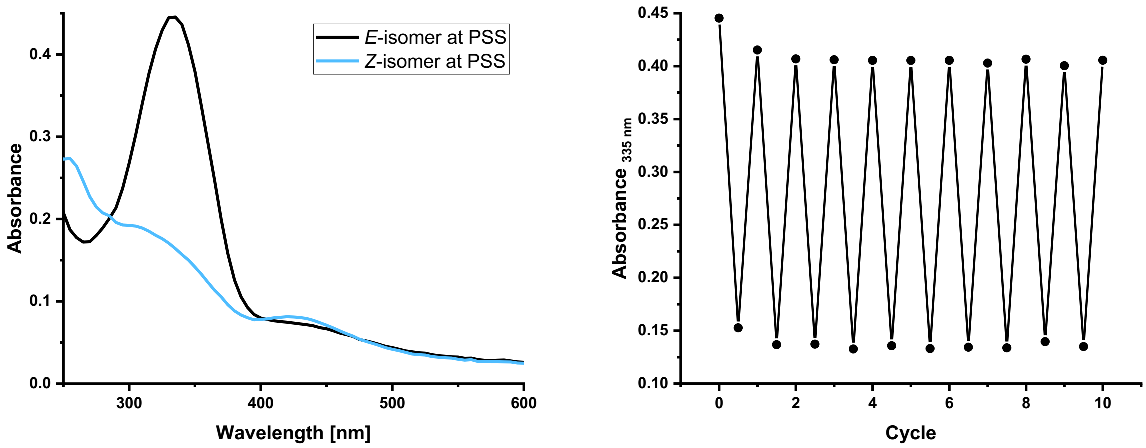

Figure S7. UV/vis spectra and cycle performance of **13**.

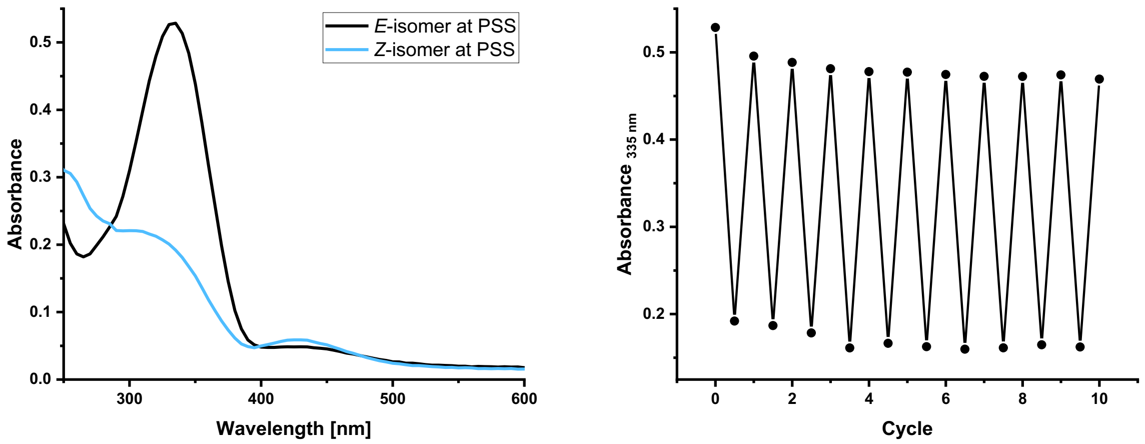

Figure S8. UV/vis spectra and cycle performance of **14**.

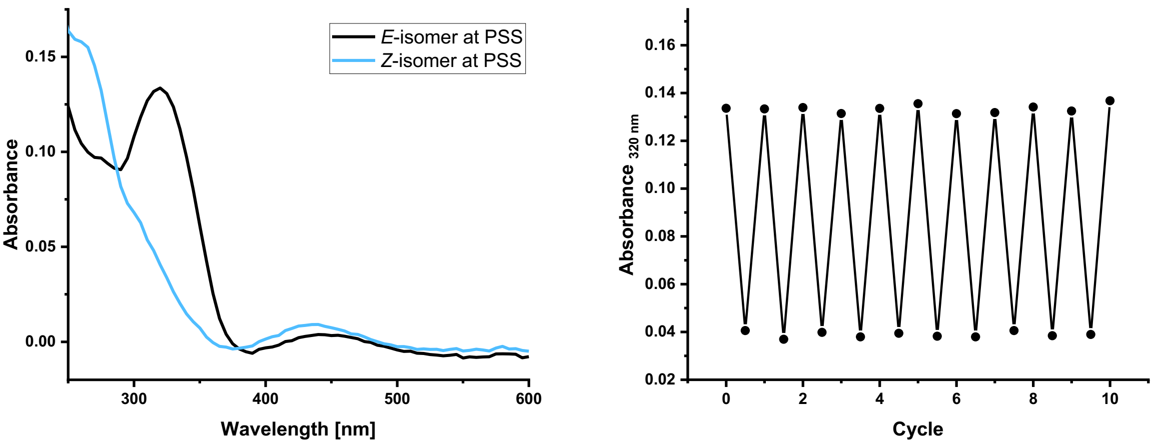

Figure S20. UV/vis spectra and cycle performance of **15**.

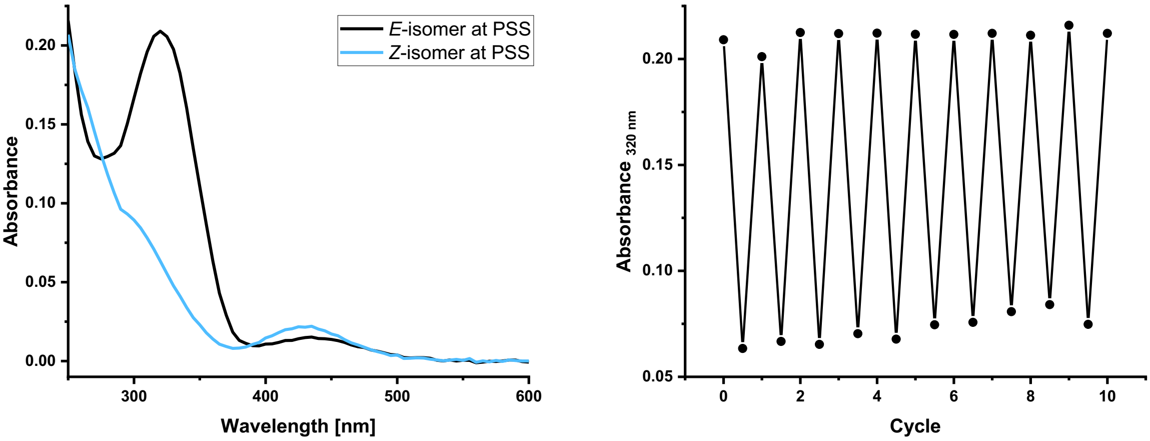

Figure S21. UV/vis spectra and cycle performance of **16**.

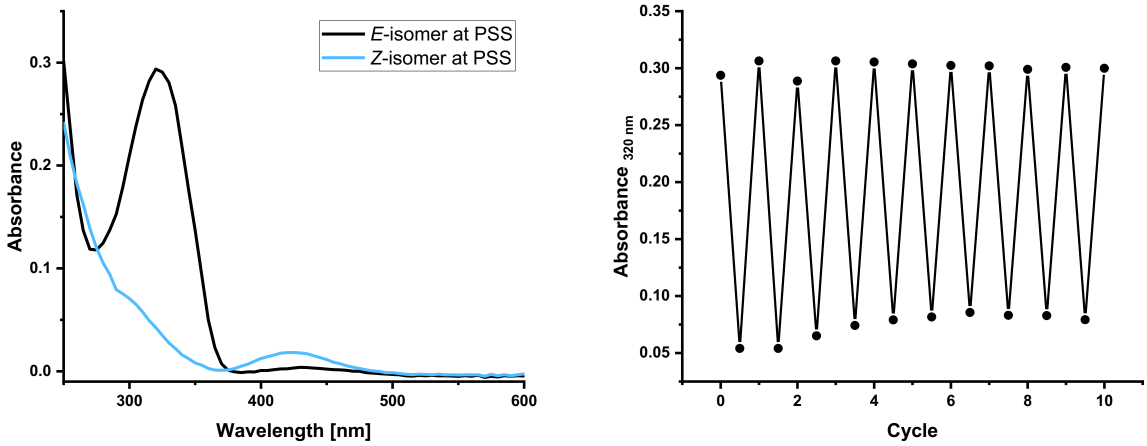

Figure S9. UV/vis spectra and cycle performance of **17**.

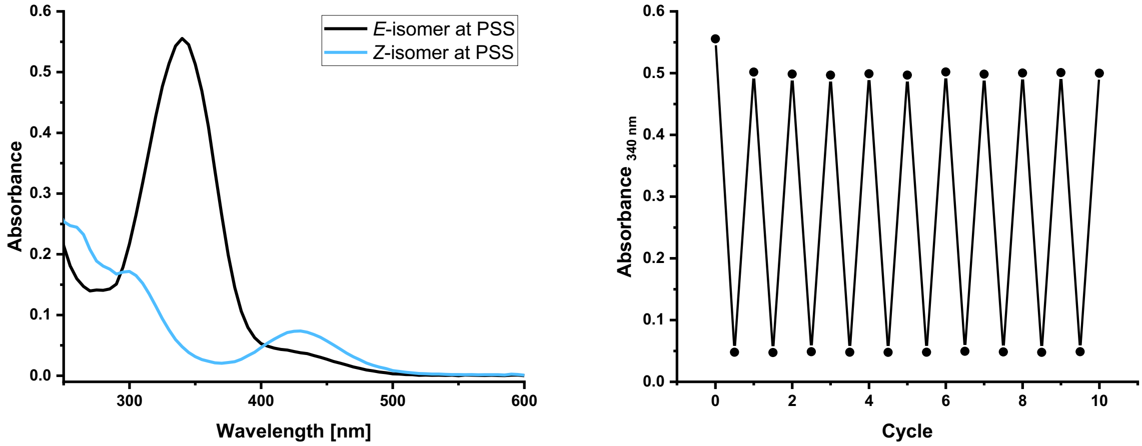

Figure S10. UV/vis spectra and cycle performance of **18**.

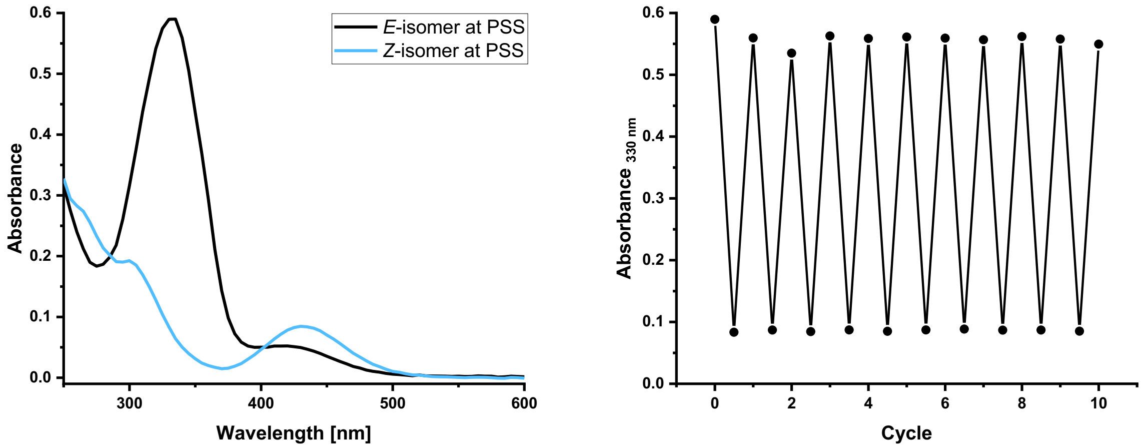

Figure S11. UV/vis spectra and cycle performance of **19**.

1.3.2. Photostationary States

Photostationary states (PSS) were measured on analytical C_{18}-RP-HPLC (flow: 0.3 mL/min, solvent A: H_2O (0.05% TFA), solvent B: MeCN). To determine the PSS of the photoswitches, the samples (in 0.1 mM in HEPES buffer + 1% DMSO, pH 7.5) were irradiated first with 340 nm to get the *Z*-isomer. Afterwards, the sample was irradiated with 420 nm (**8**-**17**) or 528 nm (**18** and **19**), respectively, to get back to the *E*-isomer. The samples were measured at the isosbestic points.

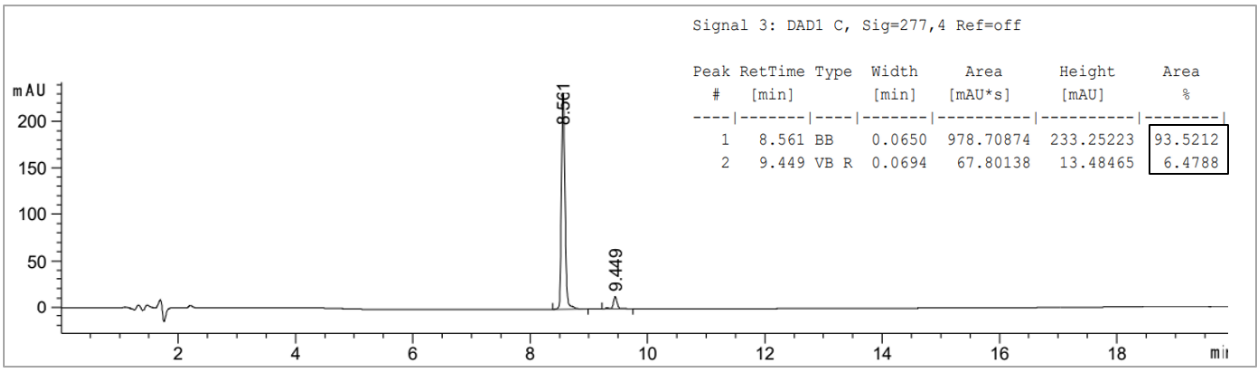

Figure S12. PSS of compound **8** after irradiation with 340 nm.

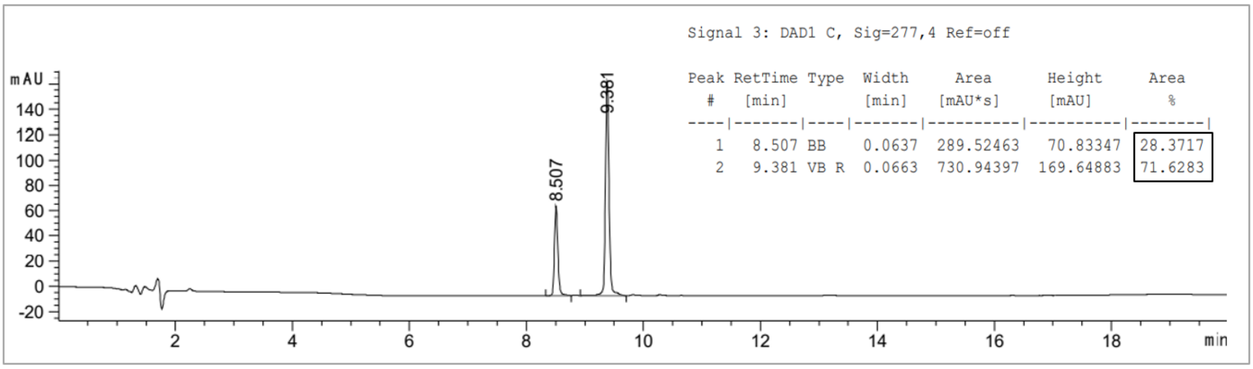

Figure S13. PSS of compound **8** after irradiation with 420 nm.

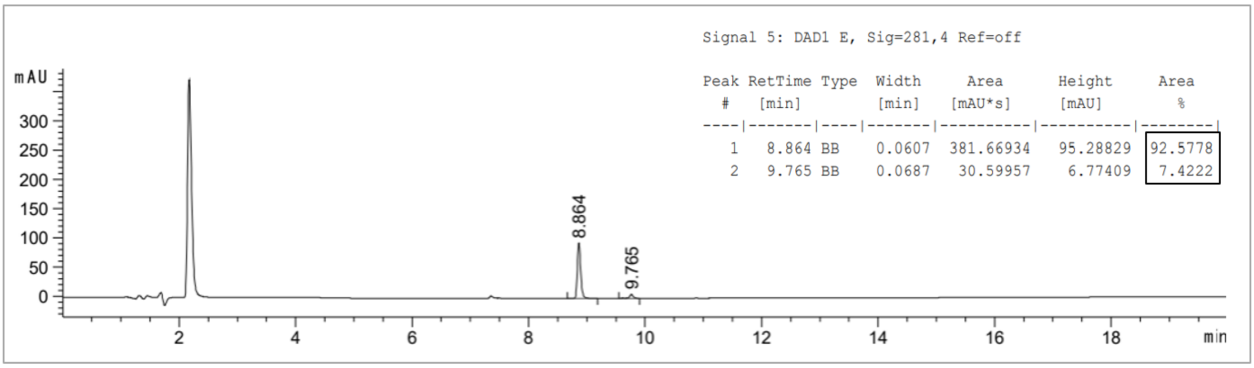

Figure S14. PSS of compound ***9*** after irradiation with 340 nm.

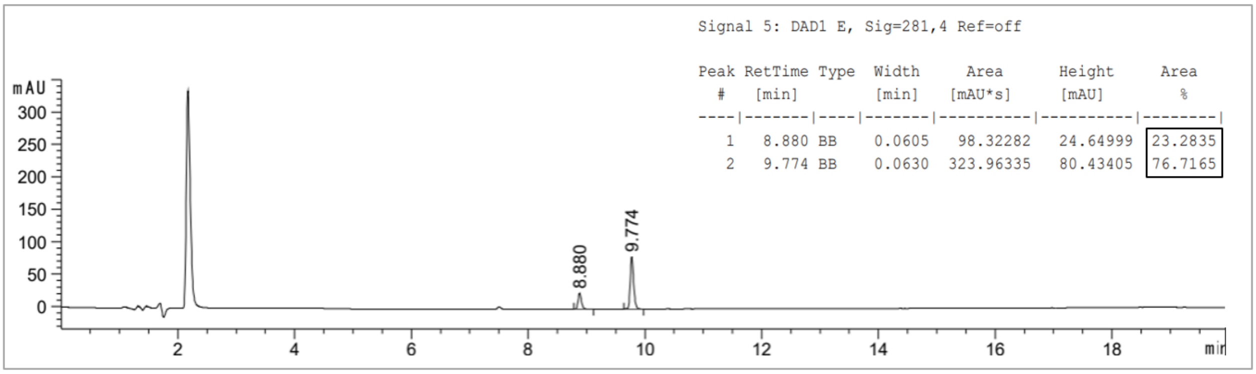

Peak #	RetTime [min]	Type	Width [min]	Area [mAU*s]	Height [mAU]	Area %
1	8.880	BB	0.0605	98.32282	24.64999	23.2835
2	9.774	BB	0.0630	323.96335	80.43405	76.7165

Figure S15. PSS of compound **9** after irradiation with 420 nm.

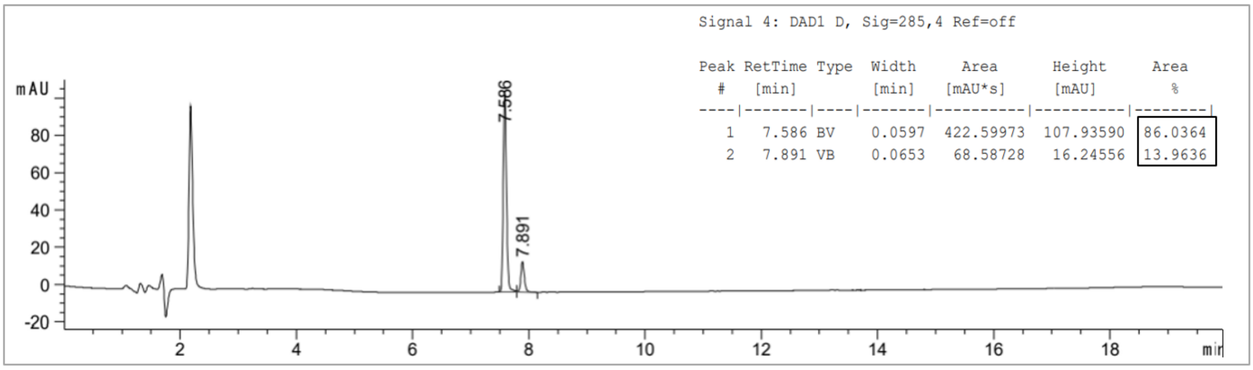

Peak #	RetTime [min]	Type	Width [min]	Area [mAU*s]	Height [mAU]	Area %
1	7.586	BV	0.0597	422.59973	107.93590	86.0364
2	7.891	VB	0.0653	68.58728	16.24556	13.9636

Figure S16. PSS of compound **10** after irradiation with 340 nm.

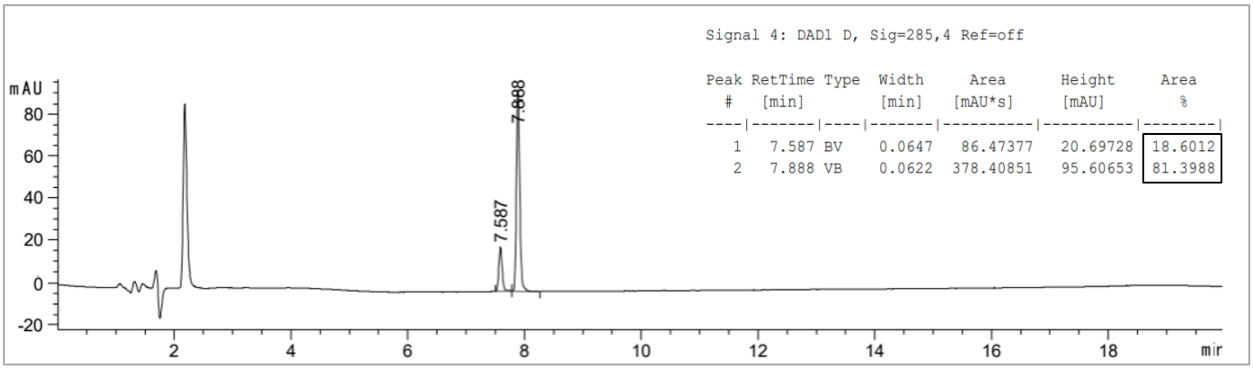

Peak #	RetTime [min]	Type	Width [min]	Area [mAU*s]	Height [mAU]	Area %
1	7.587	BV	0.0647	86.47377	20.69728	18.6012
2	7.888	VB	0.0622	378.40851	95.60653	81.3988

Figure S30. PSS of compound **10** after irradiation with 420 nm.

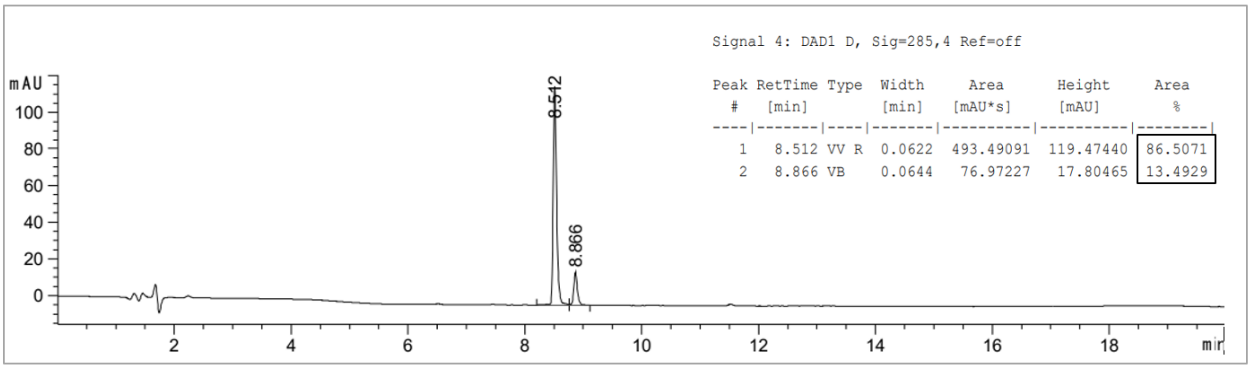

Peak #	RetTime [min]	Type	Width [min]	Area [mAU*s]	Height [mAU]	Area %
1	8.512	VV R	0.0622	493.49091	119.47440	86.5071
2	8.866	VB	0.0644	76.97227	17.80465	13.4929

Figure S31. PSS of compound **11** after irradiation with 340 nm.

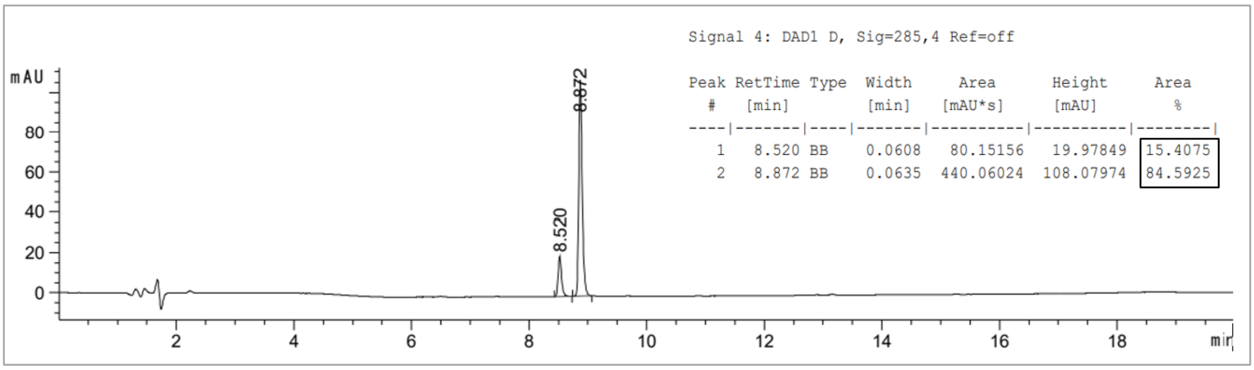

Figure S17. PSS of compound **11** after irradiation with 420 nm.

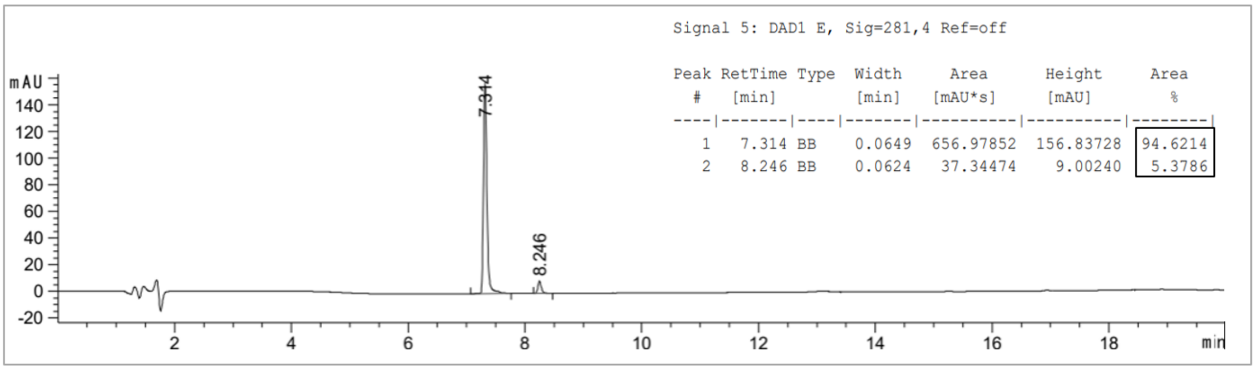

Figure S18. PSS of compound **12** after irradiation with 340 nm.

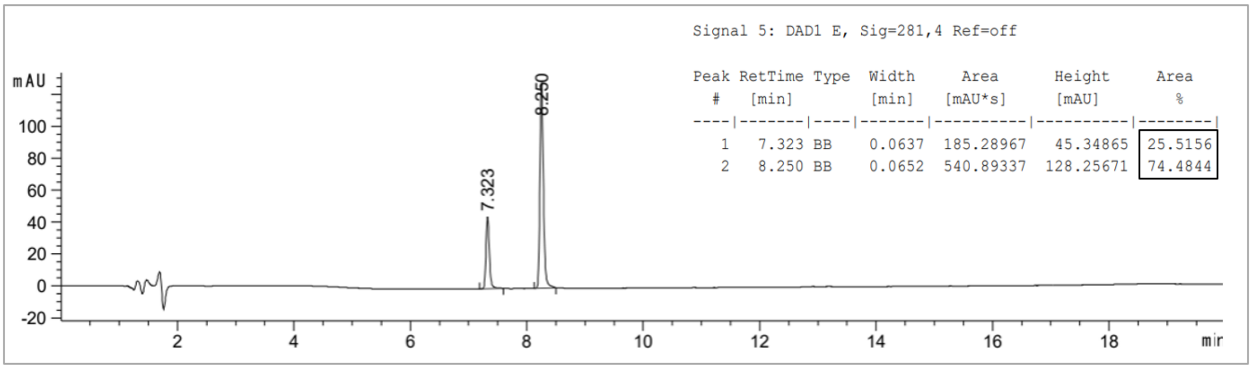

Figure S19. PSS of compound **12** after irradiation with 420 nm.

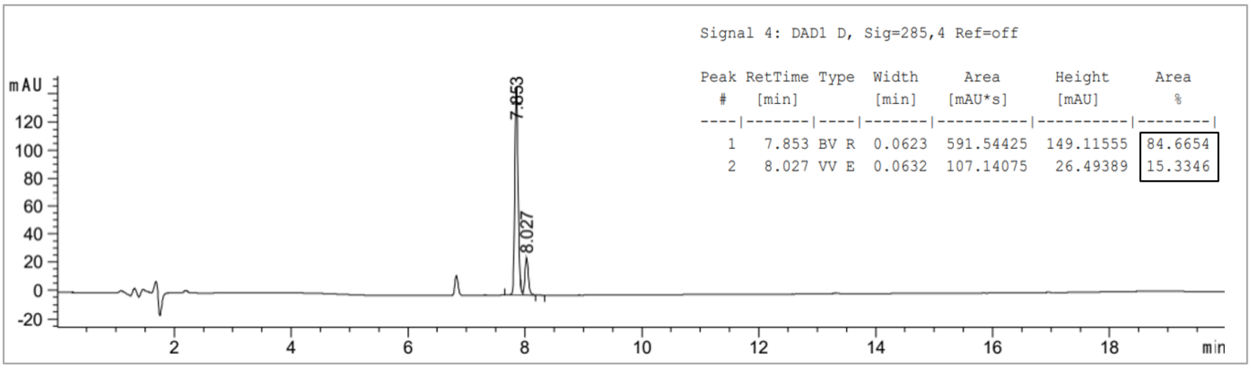

Figure S20. PSS of compound **13** after irradiation with 340 nm.

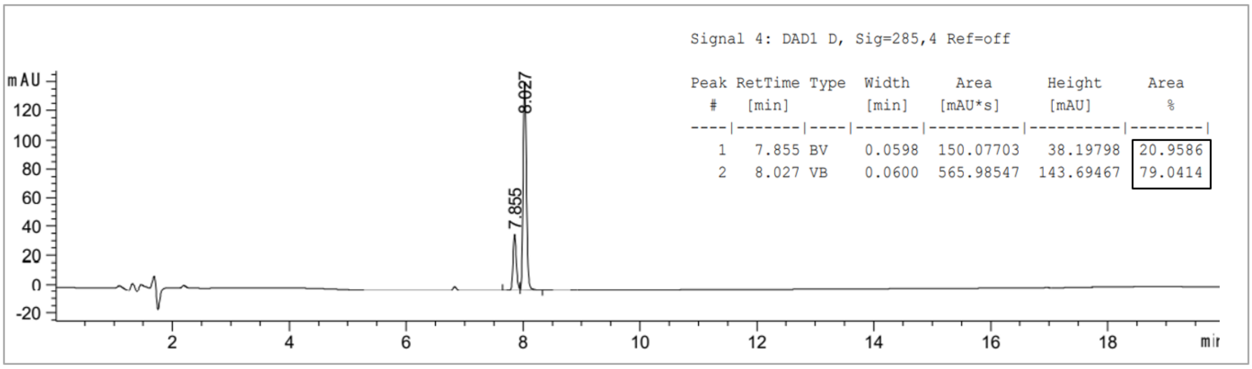

Figure S21. PSS of compound **13** after irradiation with 420 nm.

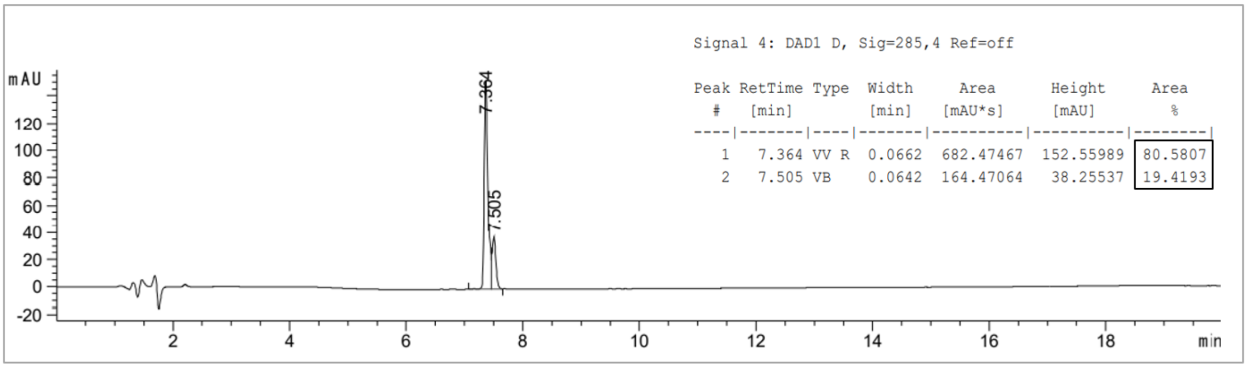

Figure S22. PSS of compound **14** after irradiation with 340 nm.

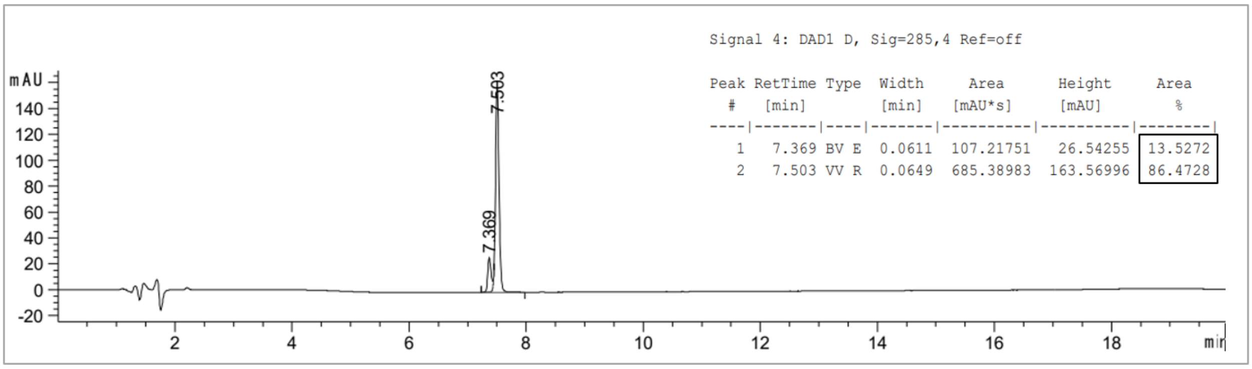

Figure S23. PSS of compound **14** after irradiation with 420 nm.

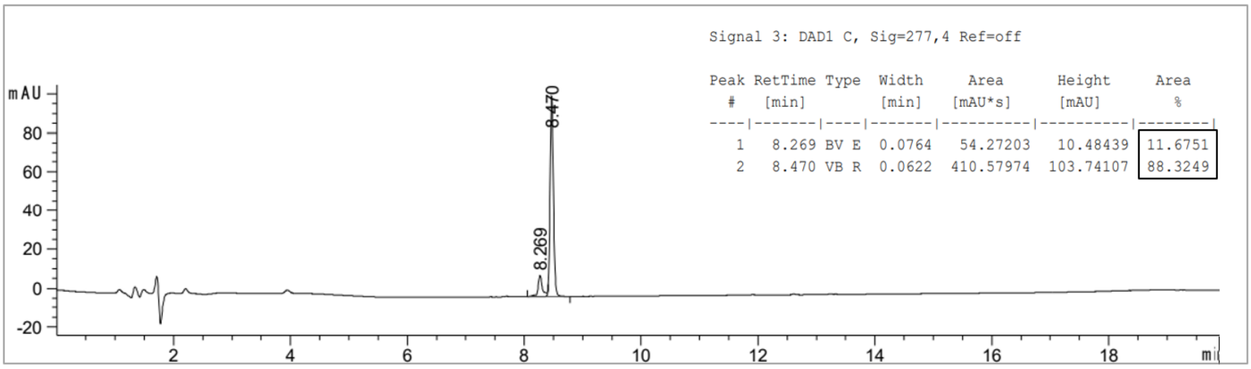

Figure S24. PSS of compound **15** after irradiation with 340 nm (here: *Z*-isomer has longer retention times than *E*-isomer).

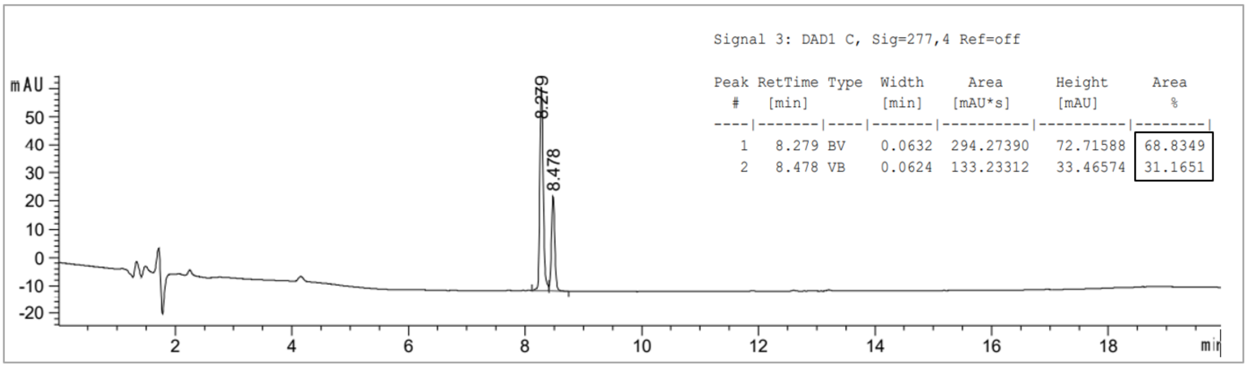

Figure S40. PSS of compound **15** after irradiation with 420 nm (here: *Z*-isomer has longer retention times than *E*-isomer).

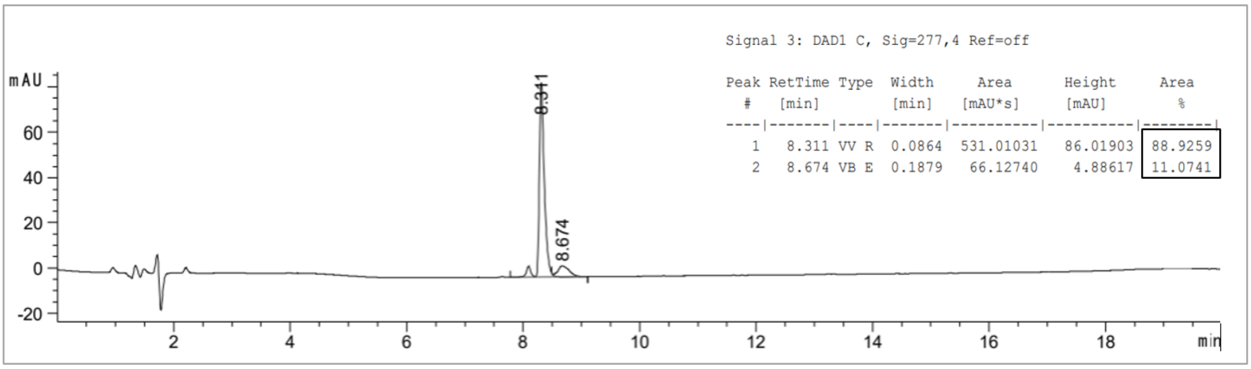

Figure S41. PSS of compound **16** after irradiation with 340 nm.

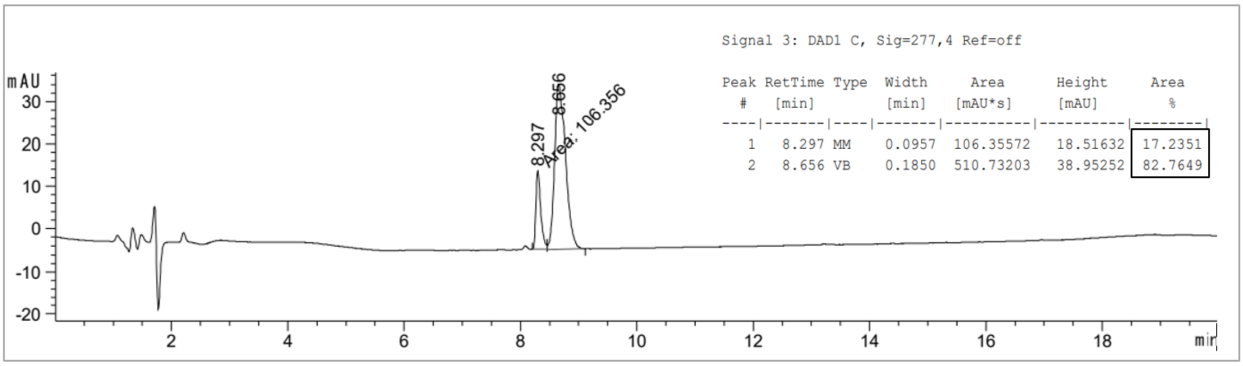

Figure S25. PSS of compound **16** after irradiation with 420 nm.

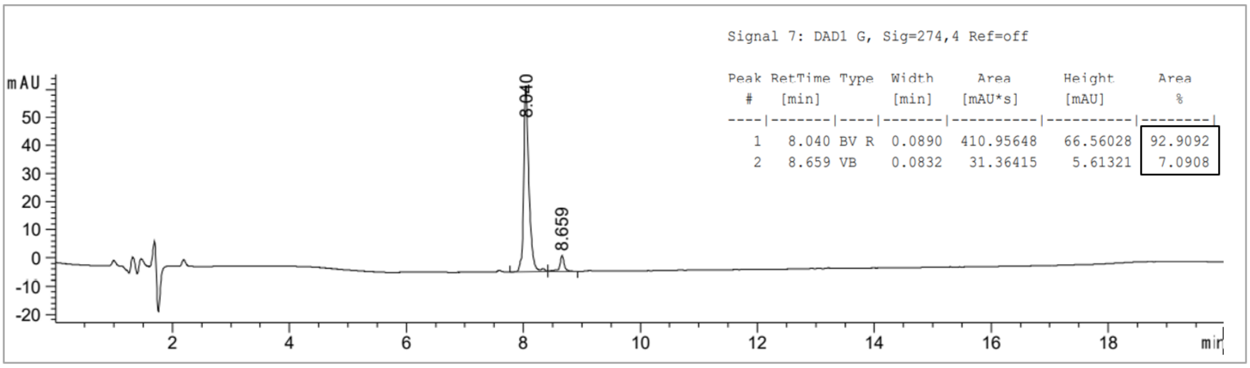

Figure S26. PSS of compound **17** after irradiation with 340 nm.

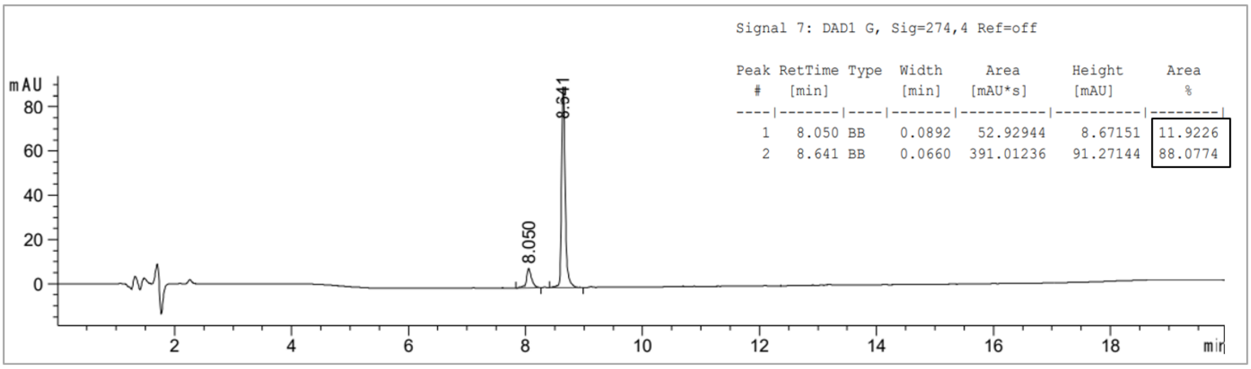

Figure S27. PSS of compound **17** after irradiation with 420 nm.

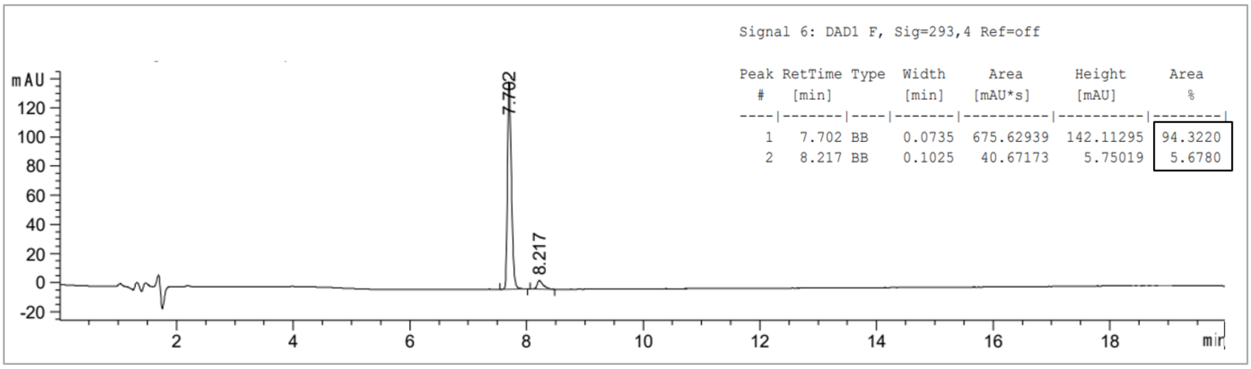

Figure S28. PSS of compound **18** after irradiation with 340 nm.

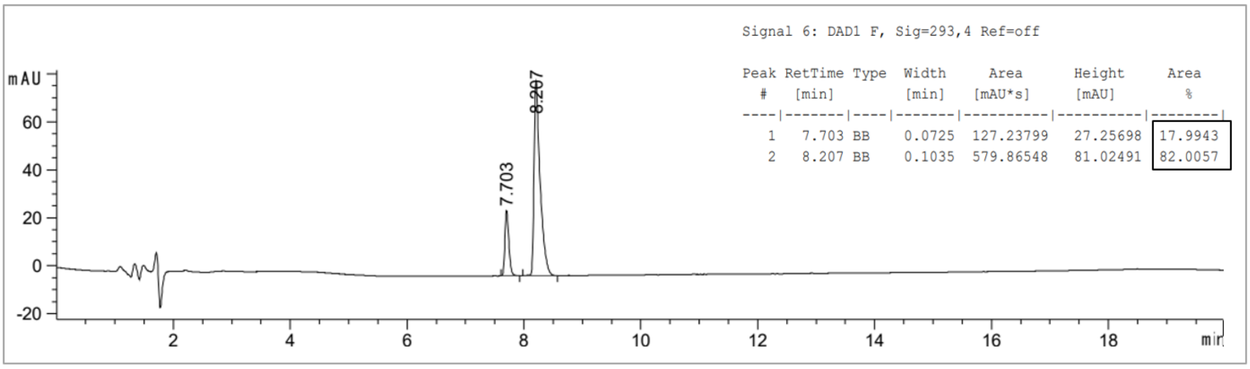

Figure S29. PSS of compound **18** after irradiation with 420 nm.

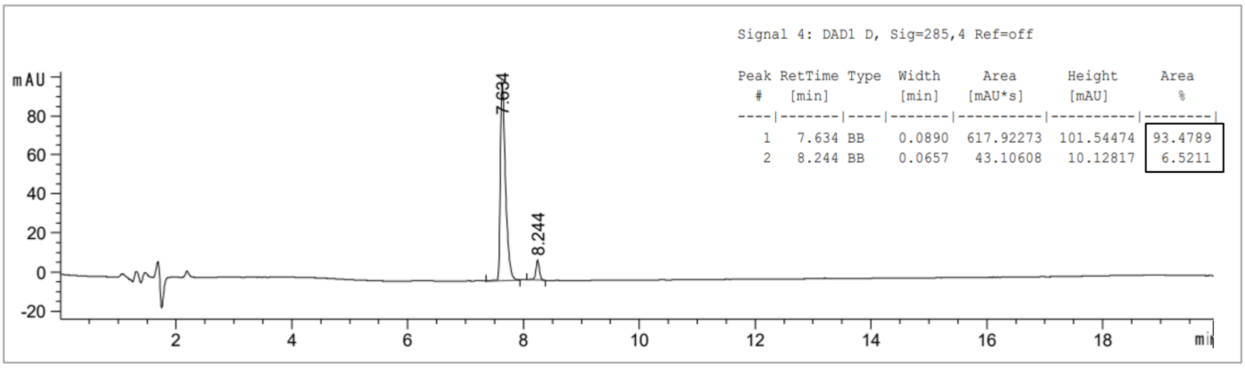

Figure S30. PSS of compound **19** after irradiation with 340 nm.

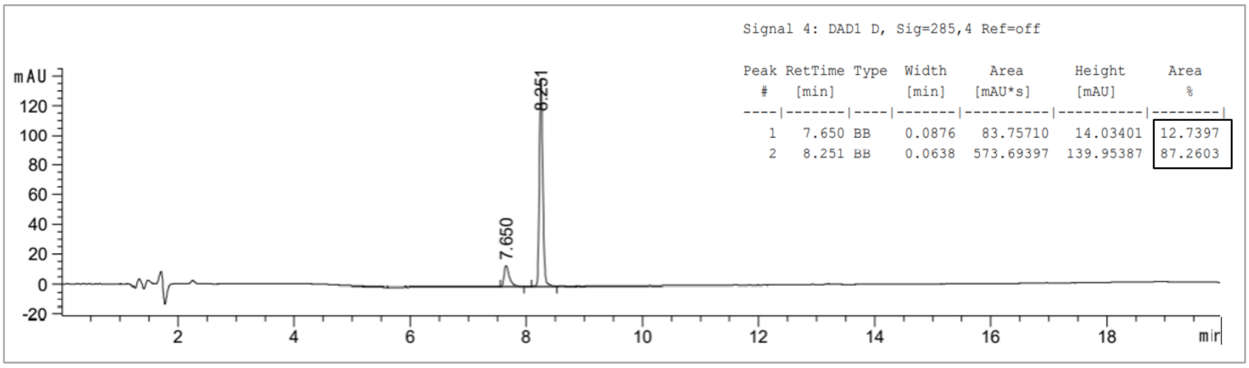

Peak #	RetTime [min]	Type	Width [min]	Area [mAU*s]	Height [mAU]	Area %
1	7.650	BB	0.0876	83.75710	14.03401	12.7397
2	8.251	BB	0.0638	573.69397	139.95387	87.2603

Figure S31. PSS of compound **19** after irradiation with 420 nm.

1.3.3. Circular dichroism (CD) Spectroscopy

CD spectra were measured on a CSP20003 Chirascan™-Plus CD spectrometer using a quartz glass cuvette with a 1 mm path length. 300 µM solutions of the samples were prepared in degassed PBS, 25% TFE/PBS and 50% TFE/PBS at physiological pH 7.4. The pH of the TFE-containing solvents was adjusted to 7.4 with 0.1 M NaOH. Spectra were recorded at 21.5 °C with n = 5 per sample in the range of 280-190 nm with 0.5 s per point and averaged. Obtained datapoints were transformed from mDeg into molar ellipticity per residue.

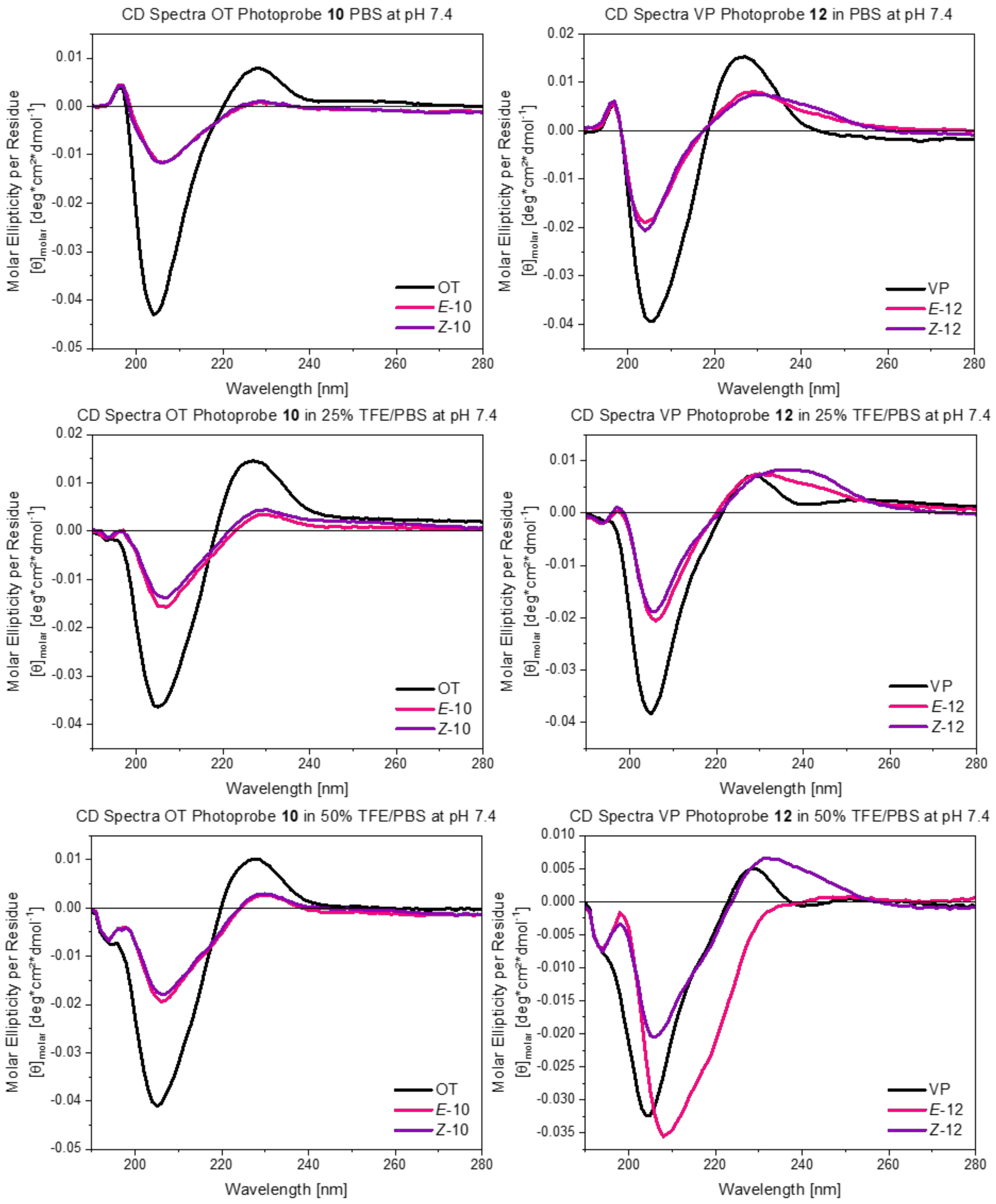

Figure S32. Circular dichroism (CD) spectra of the photoisomers of compounds **10** and **12** and their respective parent peptides OT and VP (300 µM in PBS,25% and 50% TFE/PBS, pH = 7.4).

1.3.4. Thermal Half-lives

Thermal half-lives were measured in a 96-well plate in a Thermo Scientific Multiskan® Spectrum at 25 °C (and at 37 °C for compounds **10** and **12**). The solutions (50 µM in HEPES buffer + 0.25% DMSO) were pre-irradiated with 340 nm. The absorption at 335 nm was measured every 3 h. The data was analyzed using Origin 2021.

8

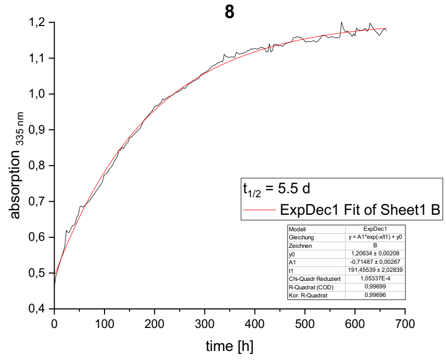

9

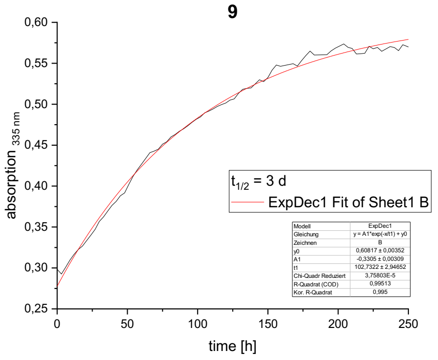

10

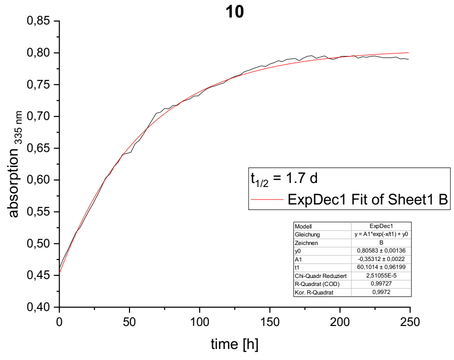

11

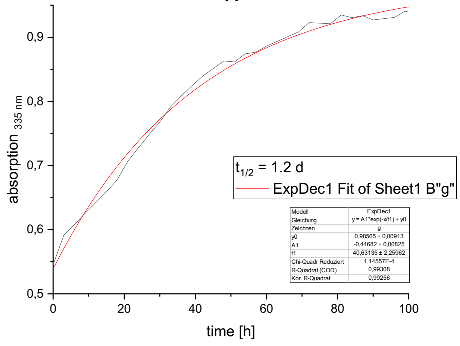

12

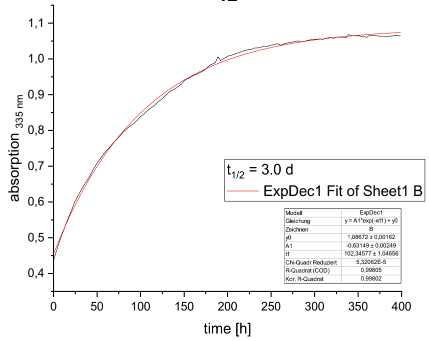

13

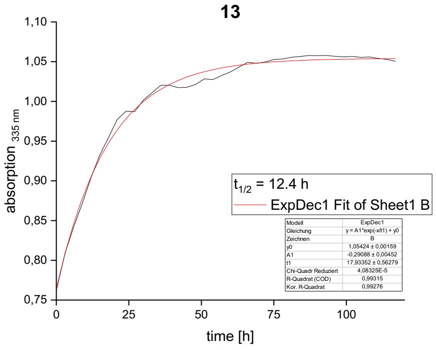

14

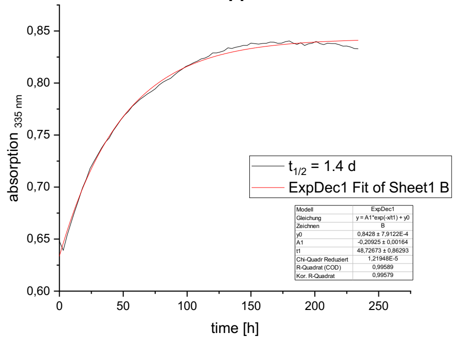

15

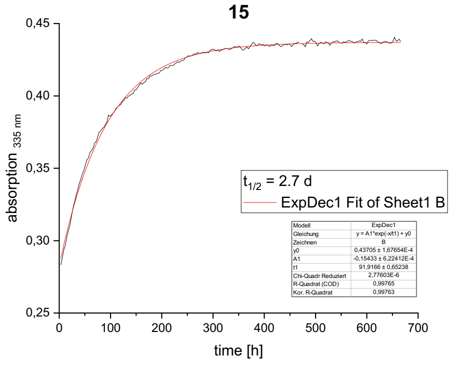

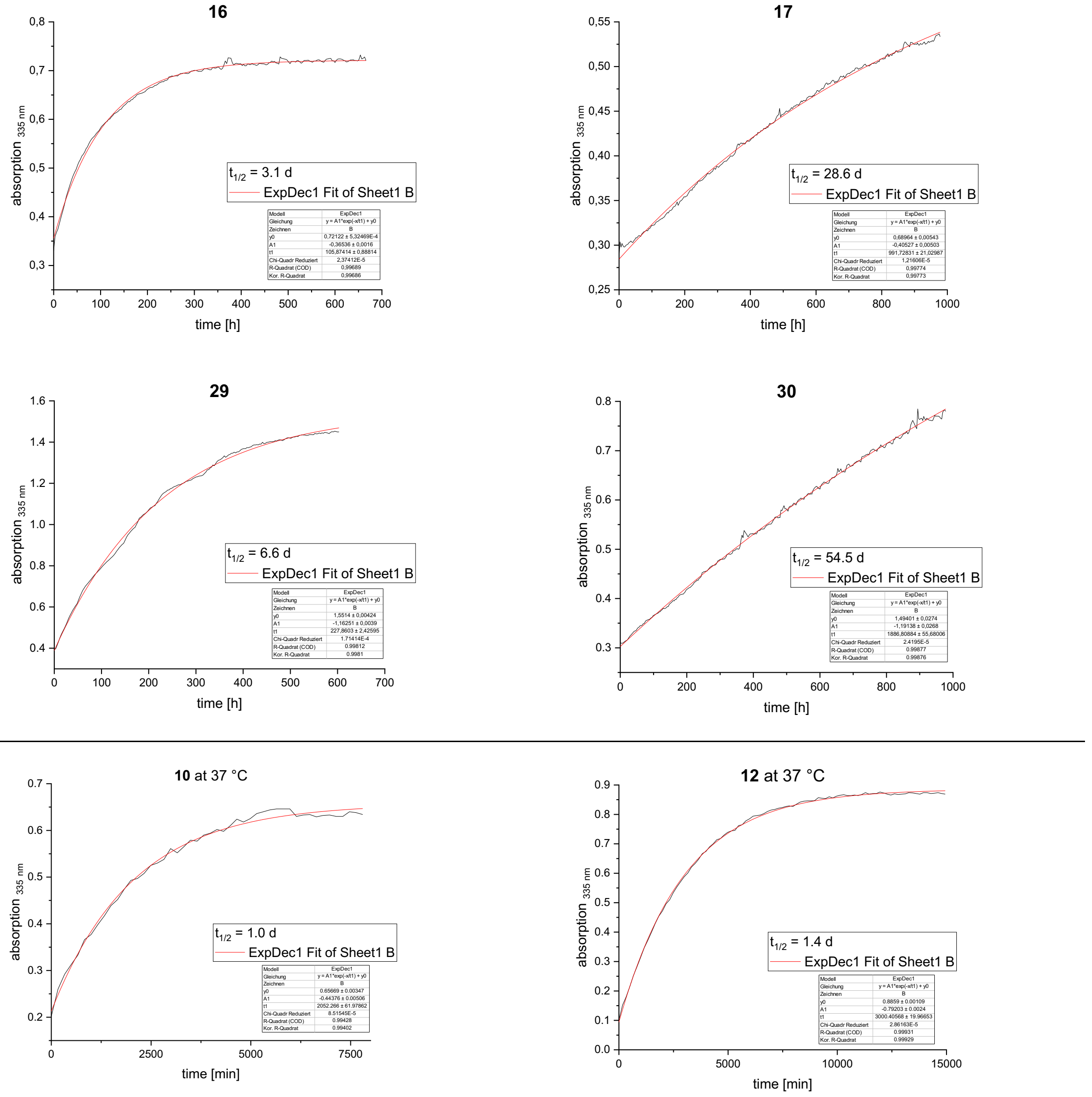

Figure S50. Thermal half-lives of compounds **8**-**19** 25 °C and of **10** and **12** at 37 °C.

2. Pharmacology

2.1. Supplementary Figures

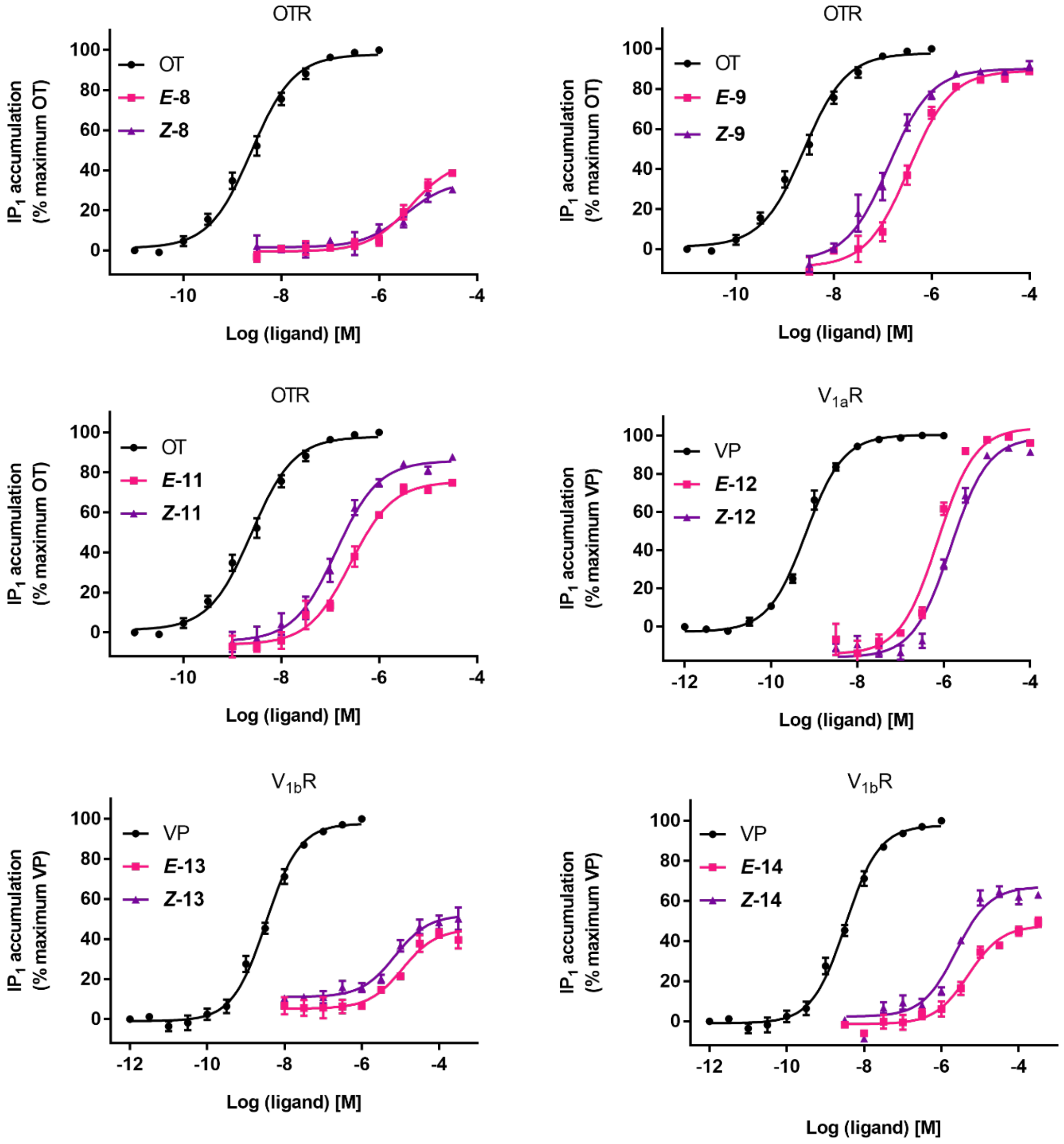

Figure S51. Full-dose-response curves of **19**, **20**, **22-25** at the respective receptor. Data represent mean values ± SEM from at least three independent experiments performed in triplicate.

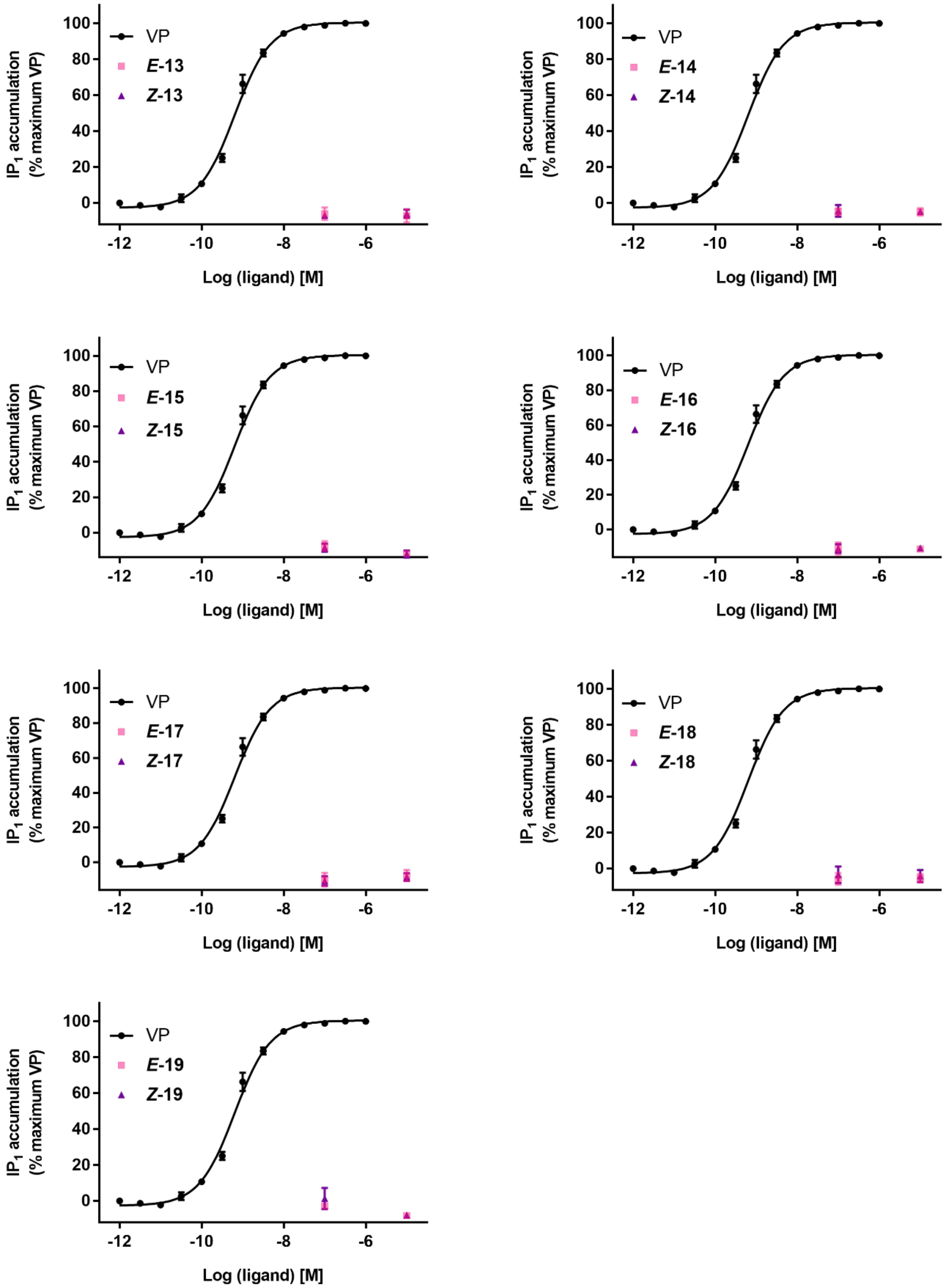

Figure S33. Two-point evaluation (100 nM and 10 µM) of compounds **13**-**19** at $V_{1a}R$. Data represent mean values ± SEM from at least three independent experiments performed in triplicate.

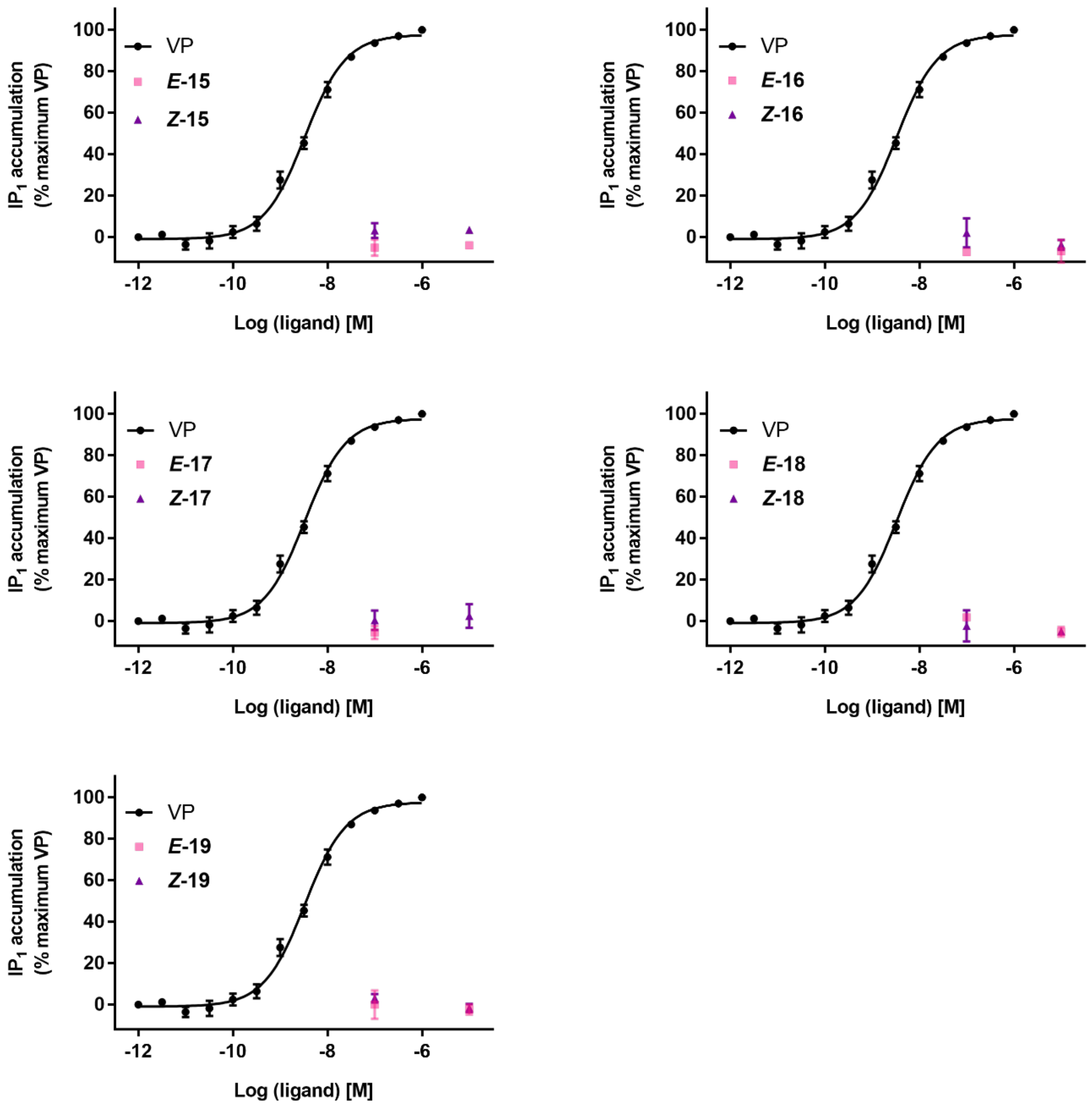

Figure S34. Two-point evaluation (100 nM and 10 µM) of compounds **15**-**19** at $V_{1b}R$. Data represent mean values ± SEM from at least three independent experiments performed in triplicate.

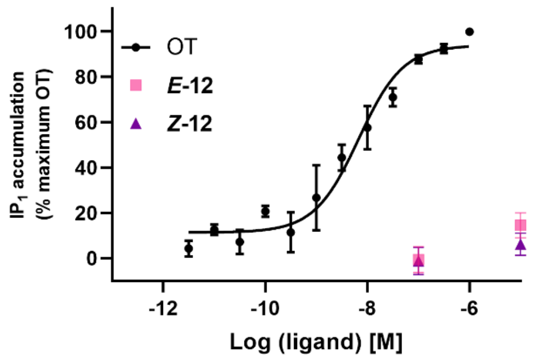

Figure S35. Two-point evaluation (100 nM and 10 µM) of compound **12** at OTR. Data represent mean values ± SEM from at least three independent experiments performed in triplicate.

3. NMR Spectra

Compound **3**

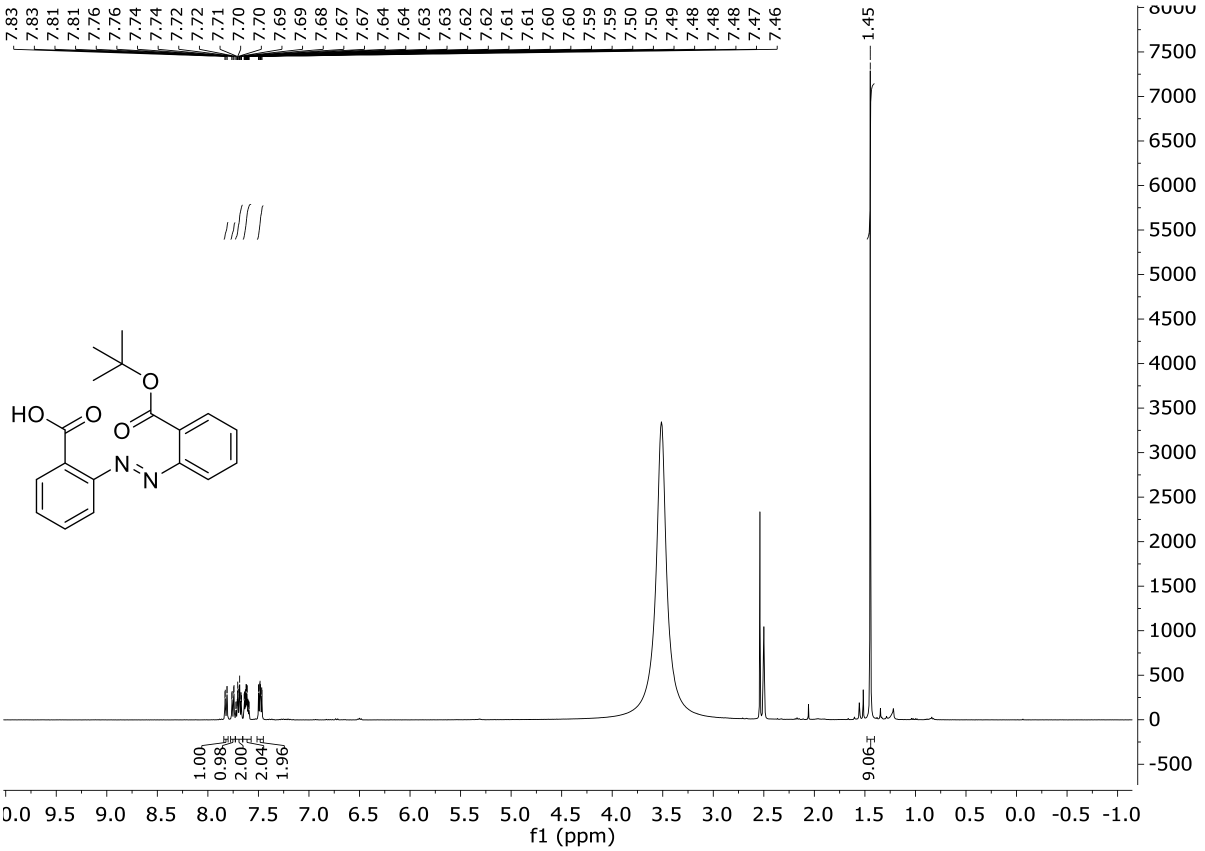

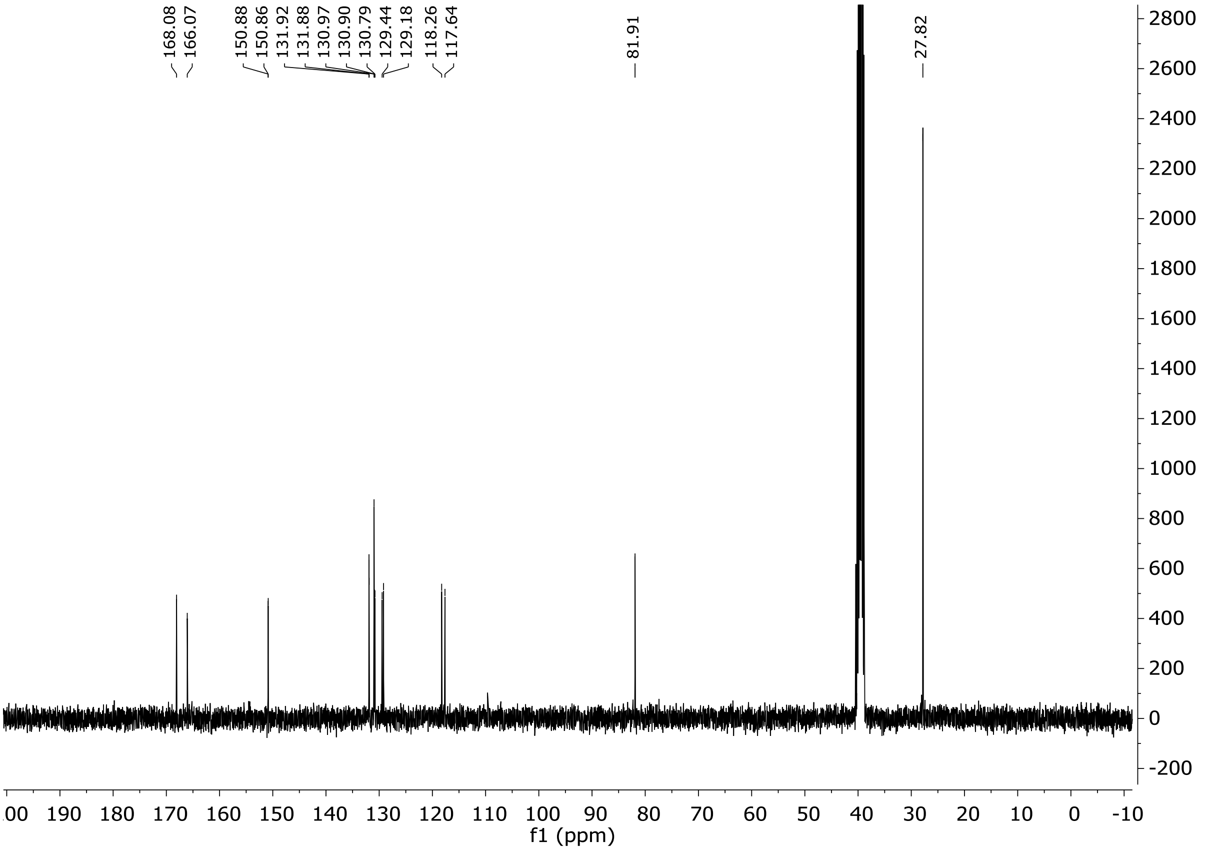

Compound **4**

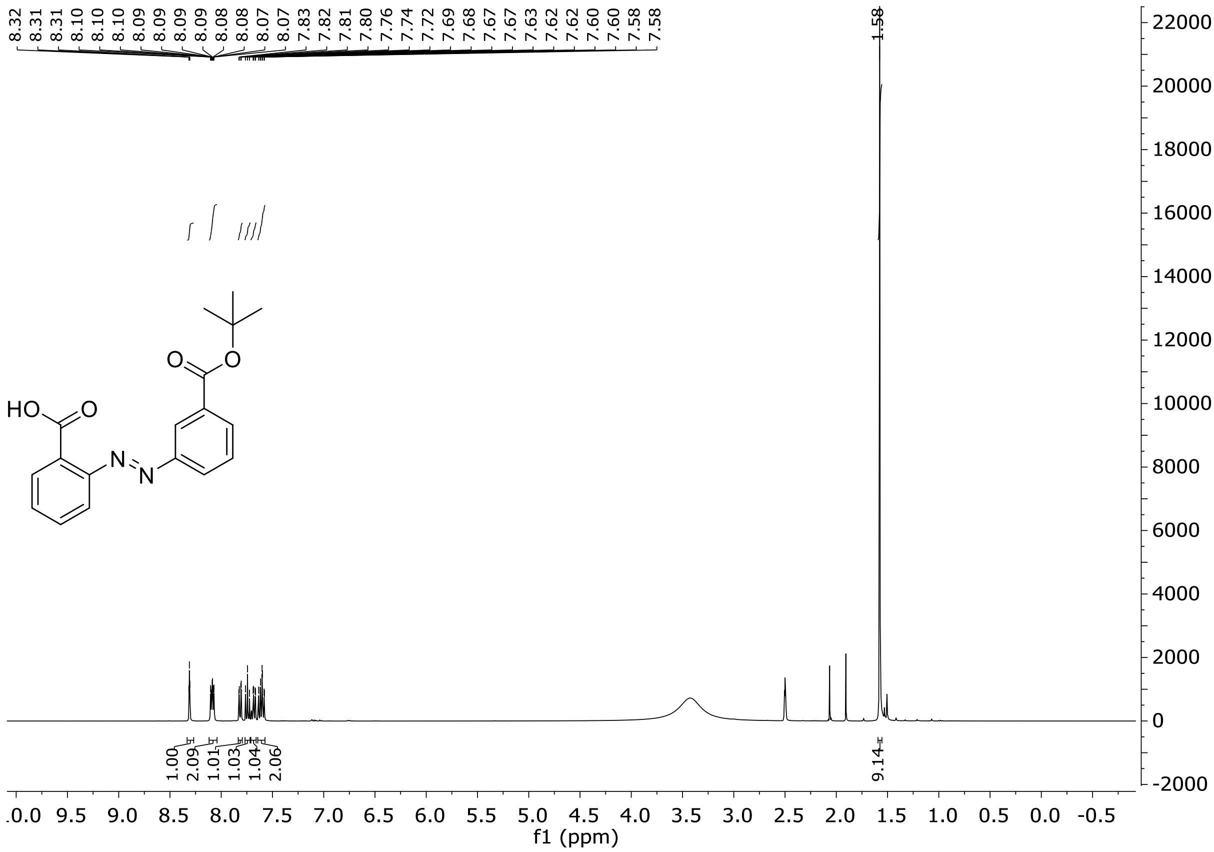

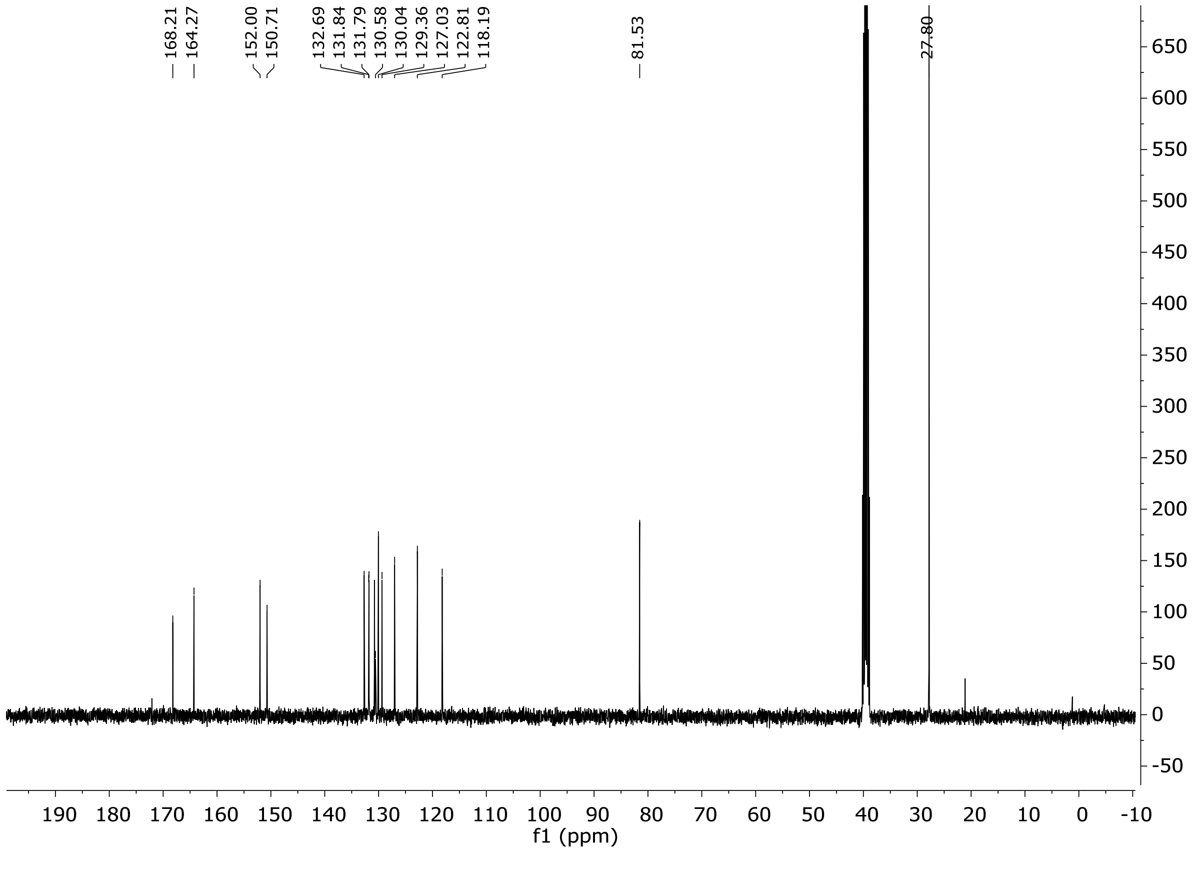

Compound **5**

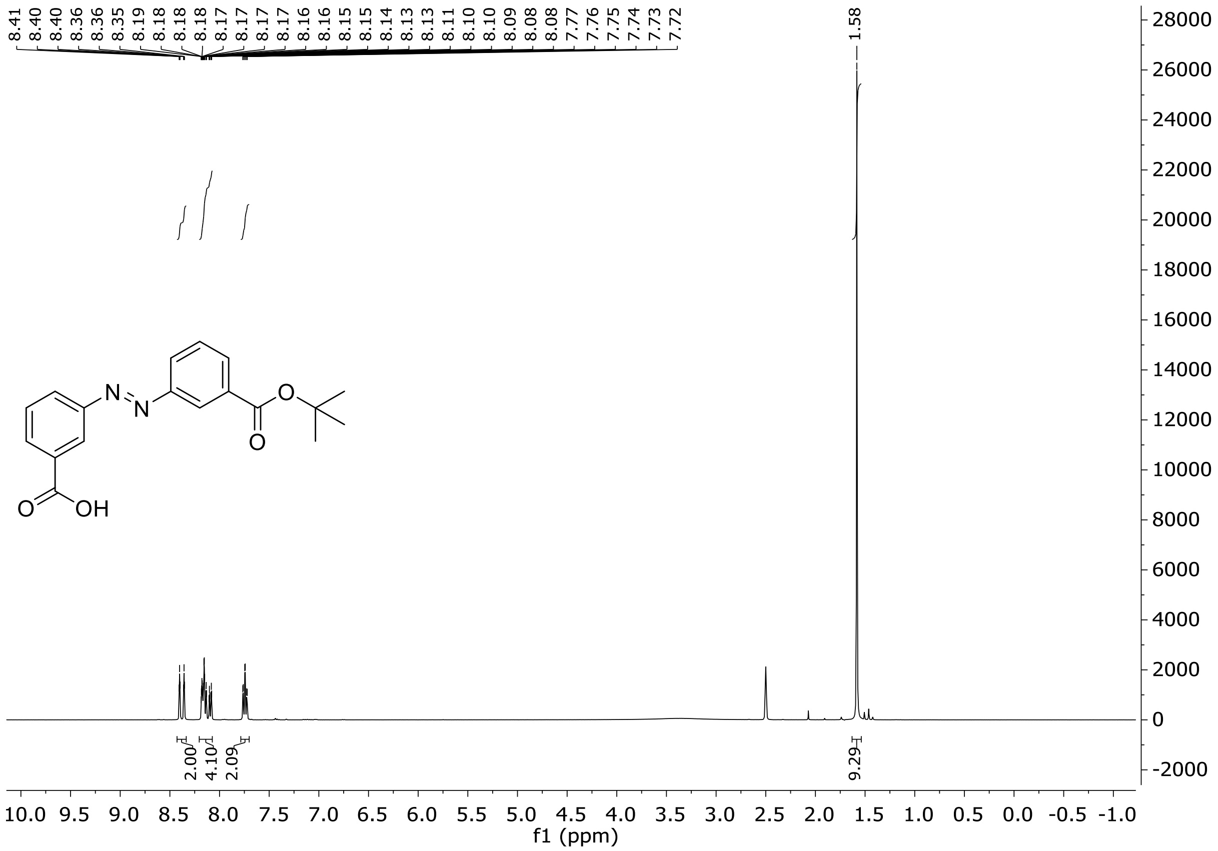
8.41 8.40 8.40 8.36 8.36 8.35 8.19 8.18 8.18 8.18 8.17 8.17 8.17 8.16 8.16 8.15 8.15 8.14 8.13 8.13 8.11 8.10 8.10 8.09 8.08 8.08 7.77 7.76 7.75 7.74 7.73 7.72
1.58
N
N
O
O
O
OH
2.00
4.10
2.09
9.29
f1 (ppm)

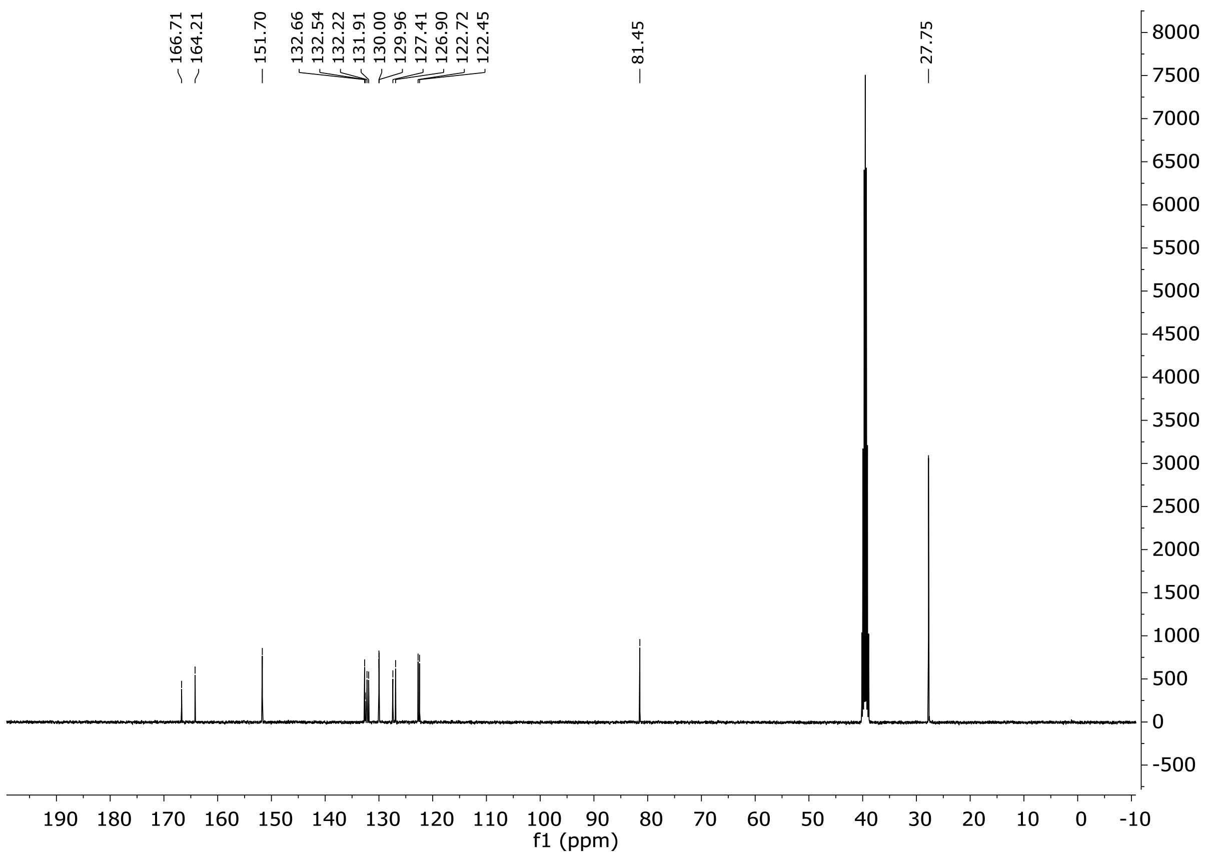
166.71 164.21
151.70
132.66 132.54 132.22 131.91 130.00 129.96 127.41 126.90 122.72 122.45
81.45
27.75
f1 (ppm)

Compound **6**

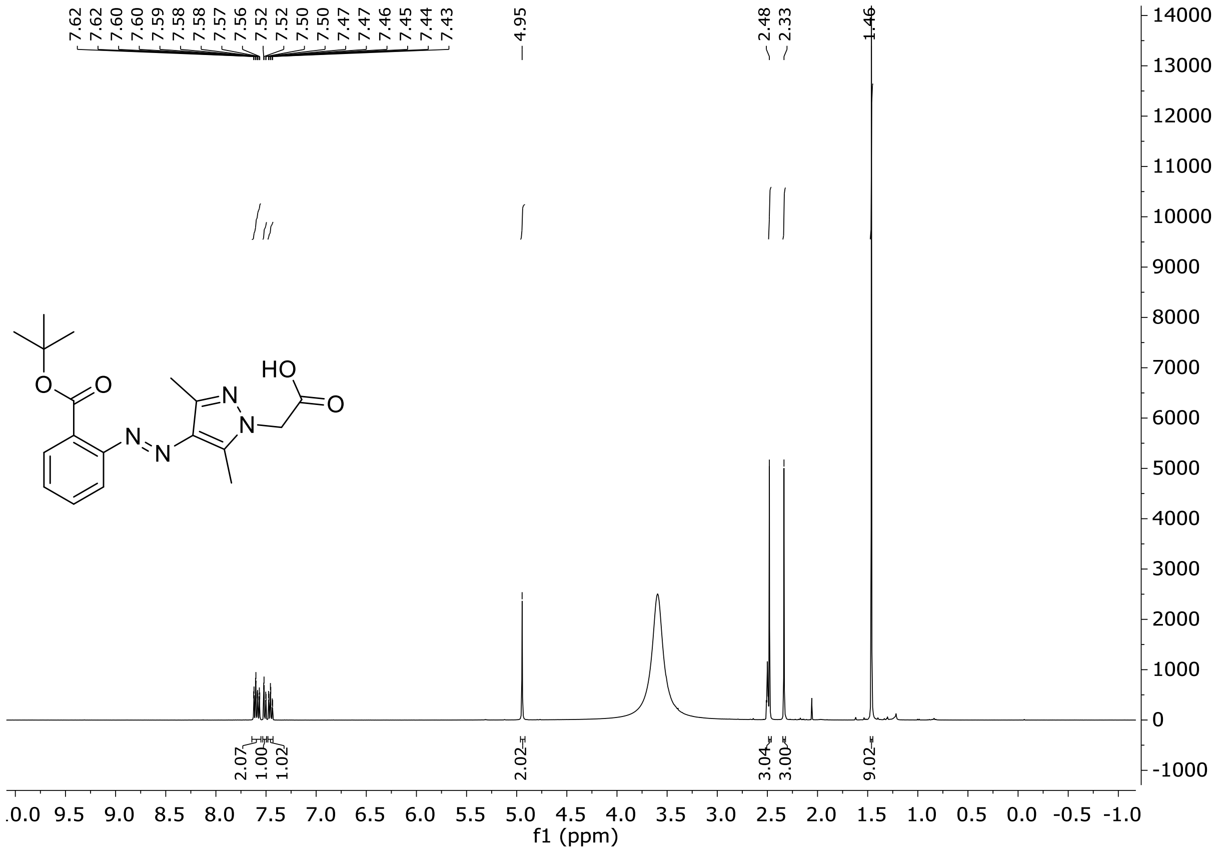

7.62 7.62 7.60 7.60 7.59 7.58 7.58 7.57 7.56 7.52 7.52 7.50 7.50 7.47 7.47 7.46 7.45 7.44 7.43
4.95
2.48
2.33
1.46
HO
O
N
N
N
N
O
O
2.07 1.00 1.02
2.02
3.04 3.00
9.02
f1 (ppm)

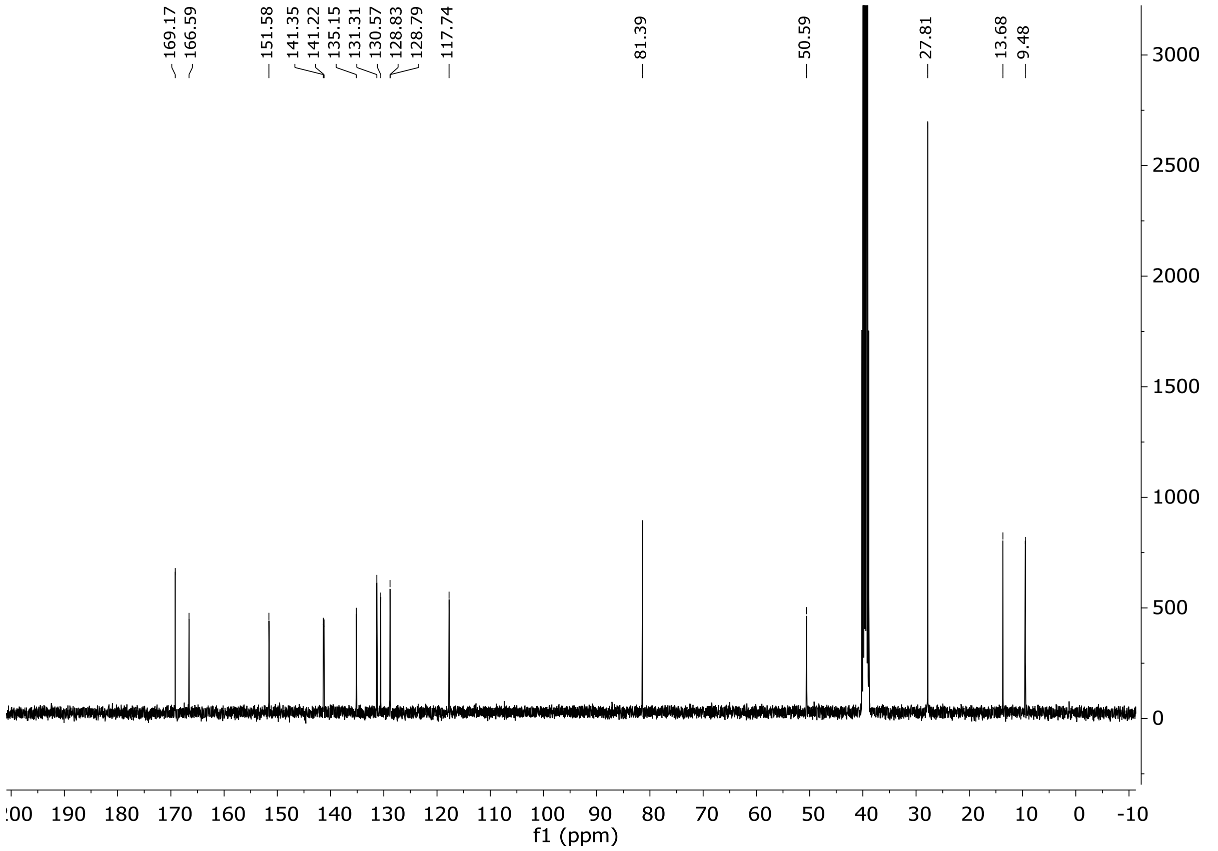

169.17 166.59
151.58 141.35 141.22 135.15 131.31 130.57 128.83 128.79 117.74
81.39
50.59
27.81
13.68 9.48
f1 (ppm)

Compound **7**

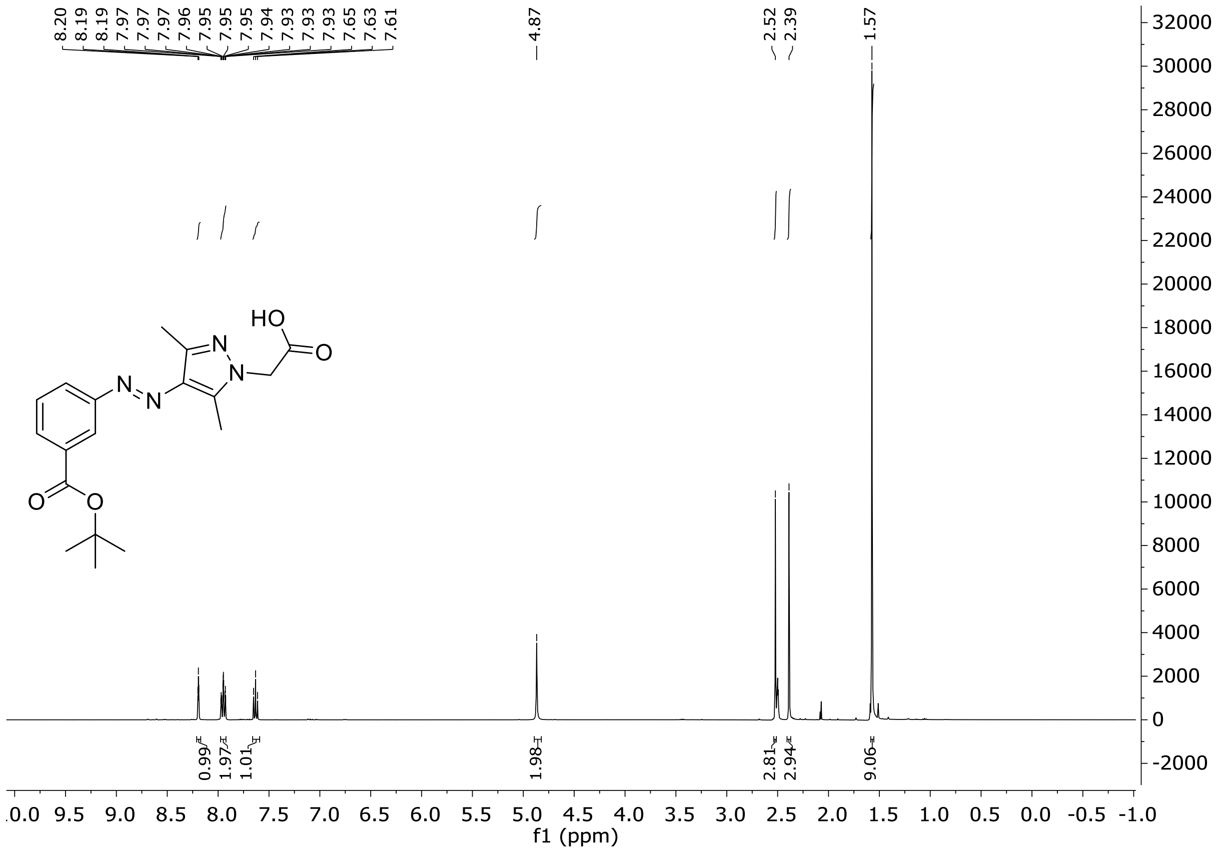
8.20 8.19 8.19 7.97 7.97 7.97 7.96 7.95 7.95 7.95 7.94 7.93 7.93 7.93 7.65 7.63 7.61
4.87
2.52 2.39
1.57
0.99 1.97 1.01 1.98 2.81 2.94 9.06
HO
O
N
N
N
N
O
O
f1 (ppm)

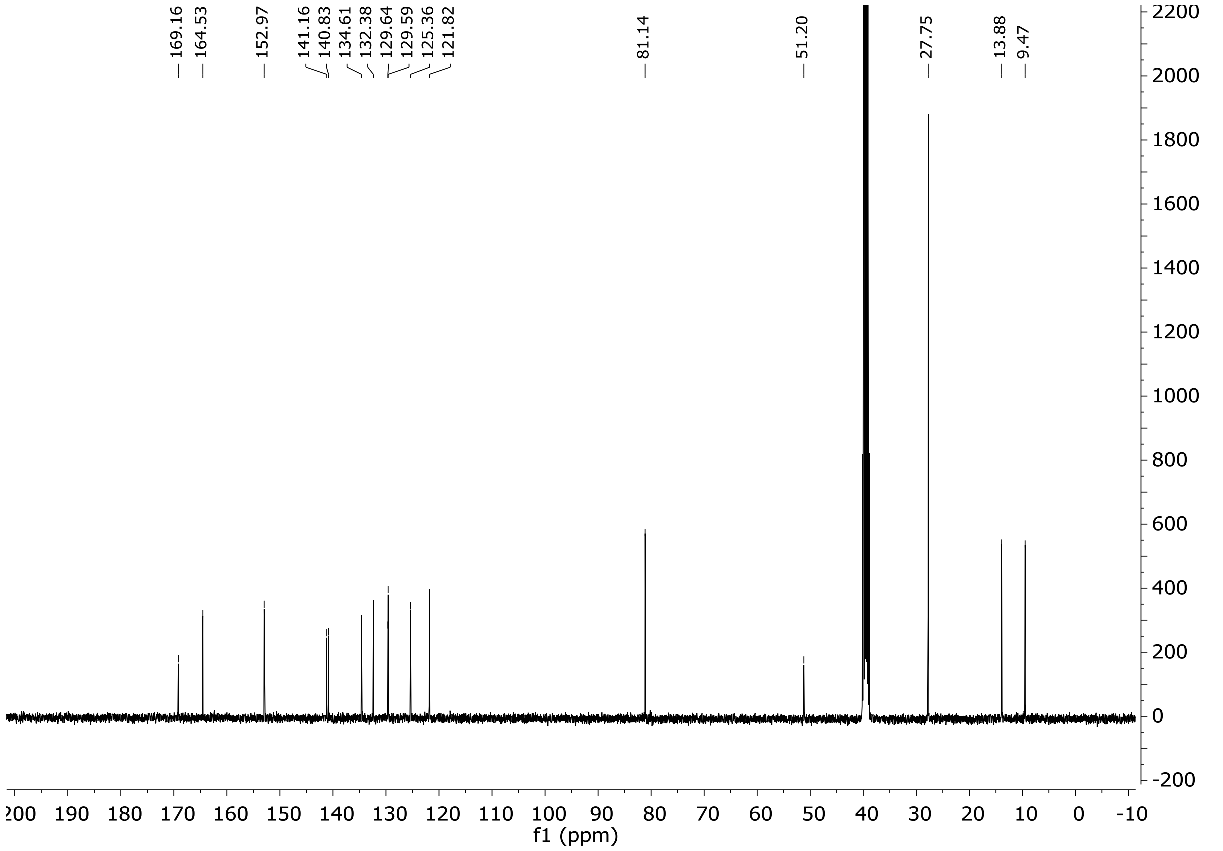
169.16 164.53 152.97 141.16 140.83 134.61 132.38 129.64 129.59 125.36 121.82
81.14
51.20
27.75
13.88 9.47
f1 (ppm)

4. References

(1) Patent: Paternostre, Marie-Therese; Cintrat, Jean-Christophe; Valery, Celine; Roux, Stephane; Rousseau, Bernard; Ijsselstijn, Maarten; Cherif-Cheikh, Roland; Artzner, F. New Octapeptide Compounds, Their Preparation, Self-Assembly Properties and Use as Ligands of Somatostatin Receptor Subtypes 2 and/or 5. WO2010037930, 2009.

(2) Albert, L.; Xu, J.; Wan, R.; Srinivasan, V.; Dou, Y.; Vázquez, O. Controlled Inhibition of Methyltransferases Using Photoswitchable Peptidomimetics: Towards an Epigenetic Regulation of Leukemia. *Chem. Sci.* **2017**, *8* (6), 4612–4618.

(3) Priewisch, B.; Rück-Braun, K. Efficient Preparation of Nitrosoarenes for the Synthesis of Azobenzenes. *J. Org. Chem.* **2005**, *70* (6), 2350–2352.
